# Supplementary material for: S3-CIMA: Supervised spatial single-cell image analysis for identifying disease-associated cell-type compositions in tissue
Source: Patterns (N Y). 2023 Aug 17;4(9):100829. doi: 10.1016/j.patter.2023.100829 (PMC10500029; doi:10.1016/j.patter.2023.100829)
Supplement: Document S1. Figures S1–S27 [file mmc1.pdf]

Patterns, Volume *4*

Supplemental information

S^3-CIMA: Supervised spatial single-cell image analysis for identifying disease-associated cell-type compositions in tissue

Sepideh Babaei, Jonathan Christ, Vivek Sehra, Ahmad Makky, Mohammed Zidane, Kilian Wistuba-Hamprecht, Christian Schürch, and Manfred Claassen

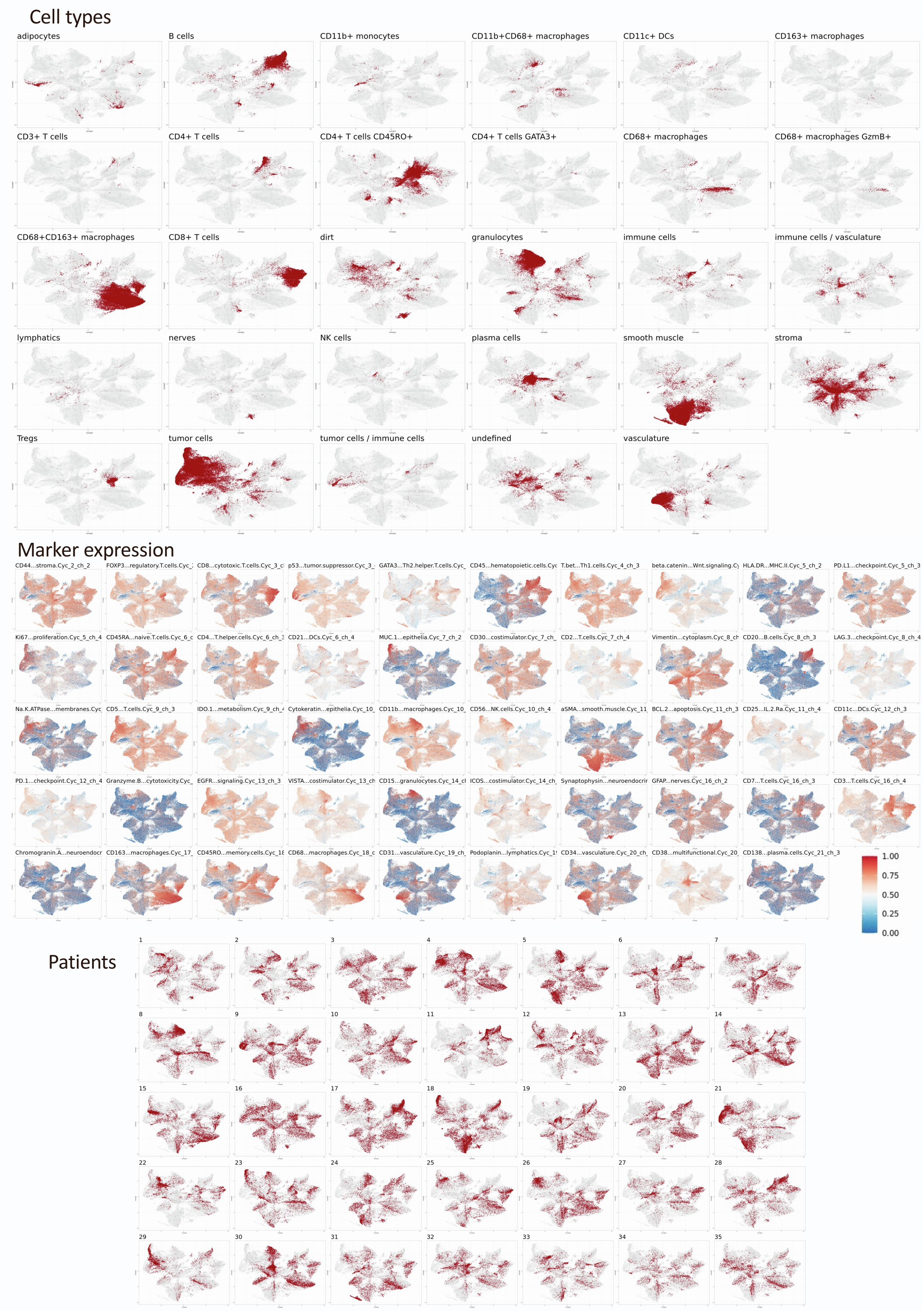

Fig. S1.

CRC data

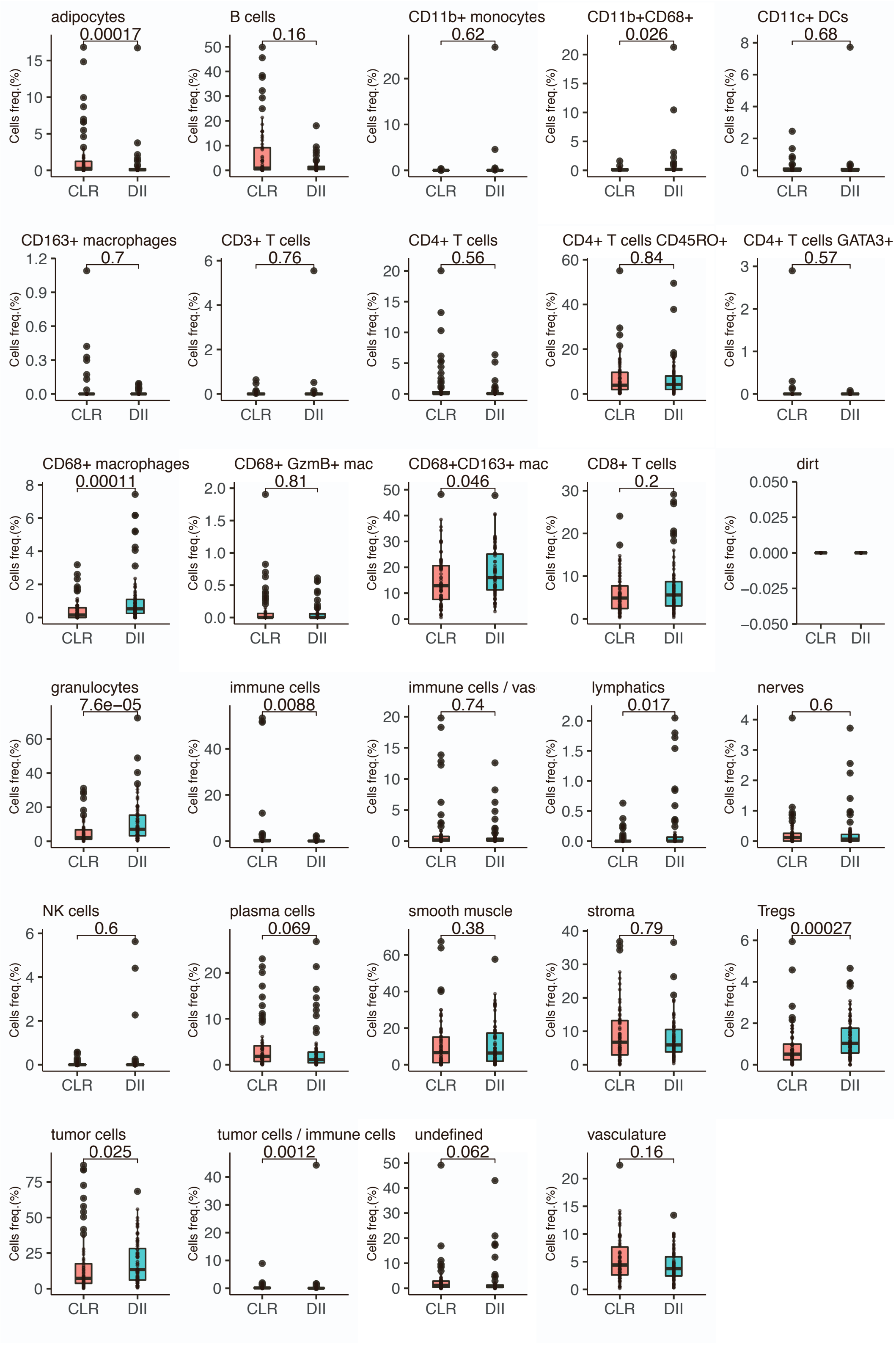

Fig. S2.

Box plot of frequency of each cell type in each CRC group (CLR vs DII)

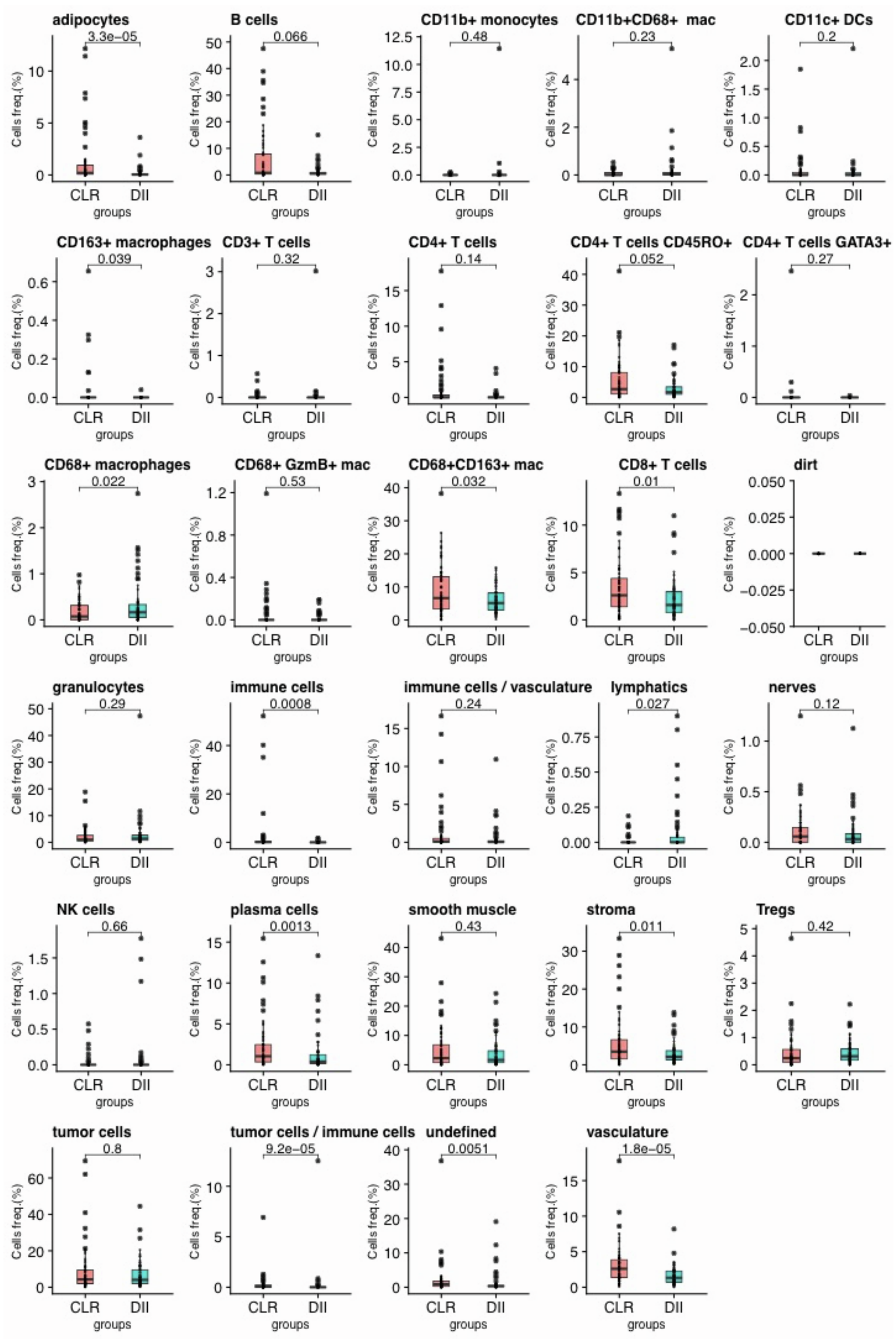

Fig. S3.

Box plot of frequency of selected cell obtained from S[3]-CIMA global enrichment analysis at k = 30 in each CRC group per cell type.

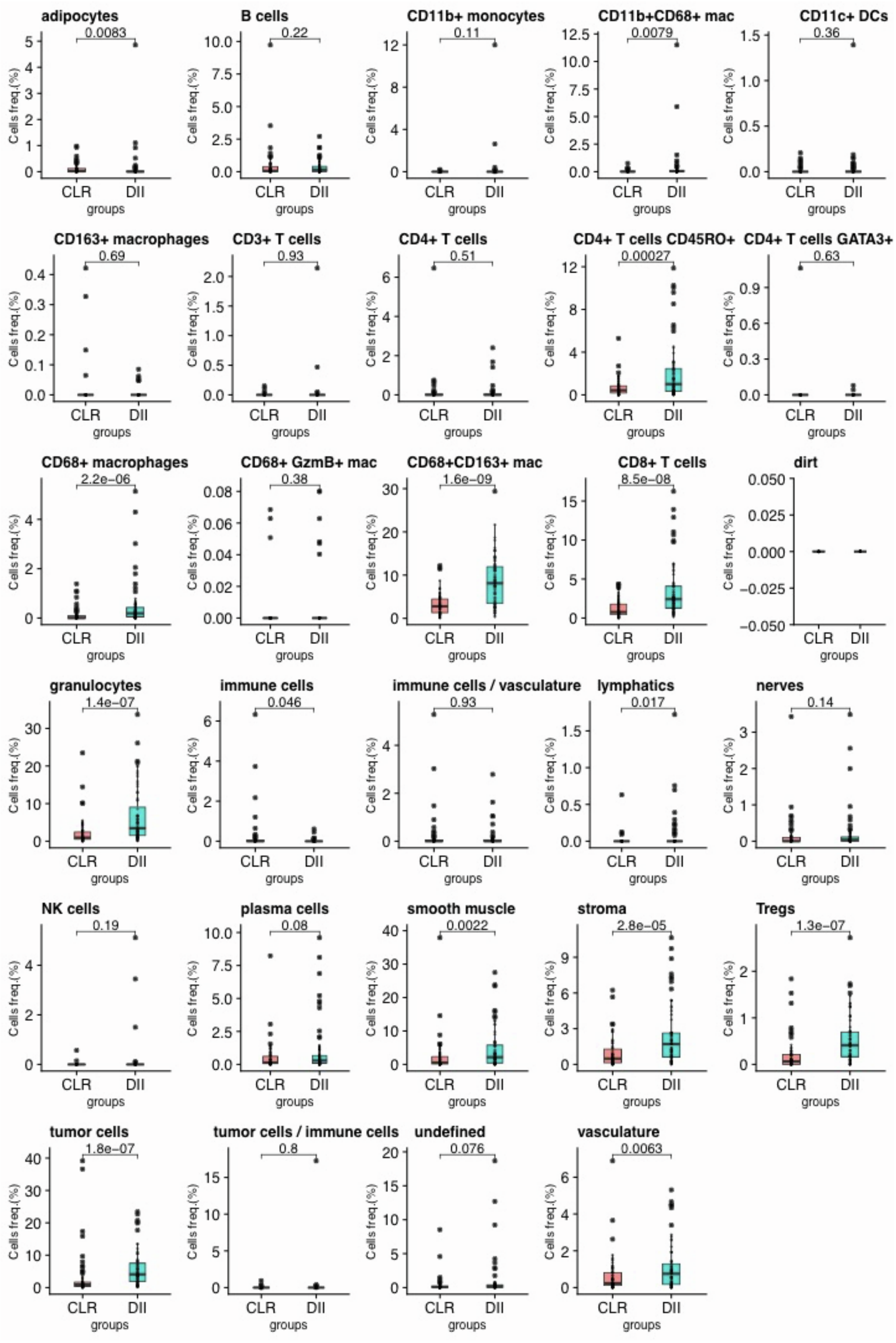

Fig. S4.

Box plot of frequency of selected cell obtained from S^3-CIMA global enrichment analysis at k = 50 in each CRC group per cell type.

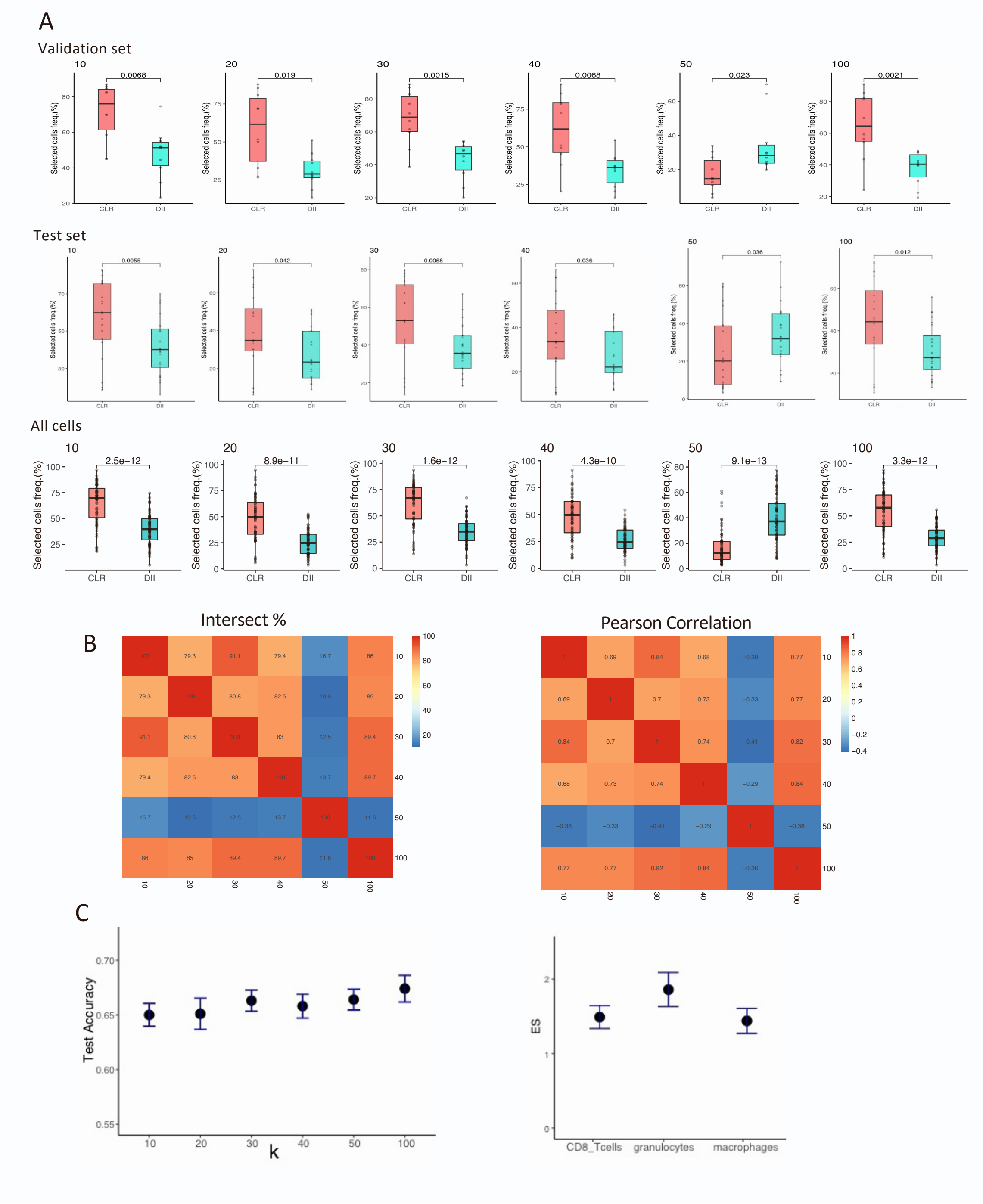

Fig. S5.

The frequency of selected cells between groups using S^3-CIMA global enrichment analysis across different cell neighborhood sizes (10 to 100) using different subset of cells. Heatmaps show the overlap (%) and correlation between the set of selected cells over the different neighborhood sizes.

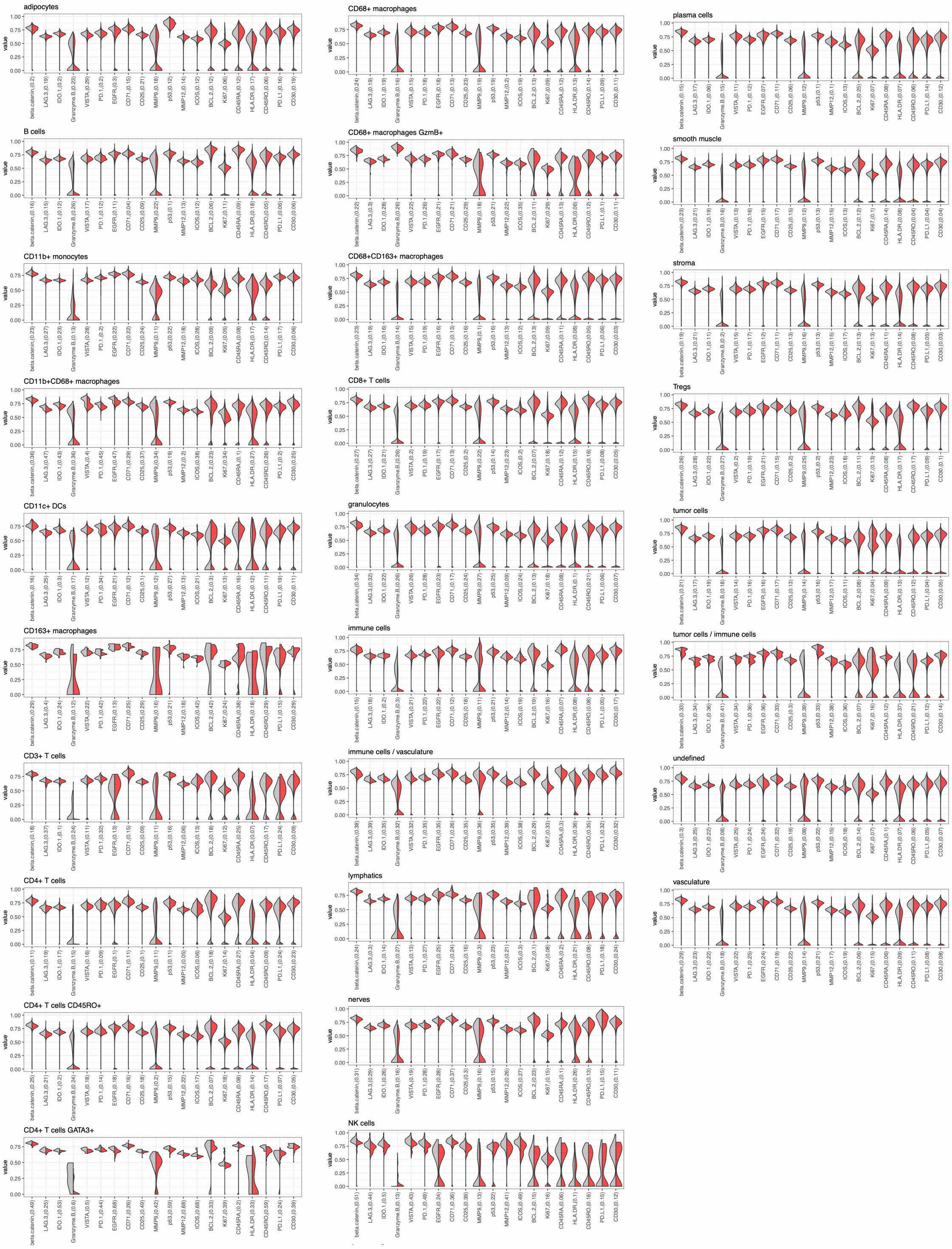

Fig. S6.

S^3-CIMA global enrichment analysis at k =30, Density of functional marker expression showing greatest differential abundance in terms of the Kolmogorov–Smirnov two-sample test between the selected and non-selected cell subsets per cell types.

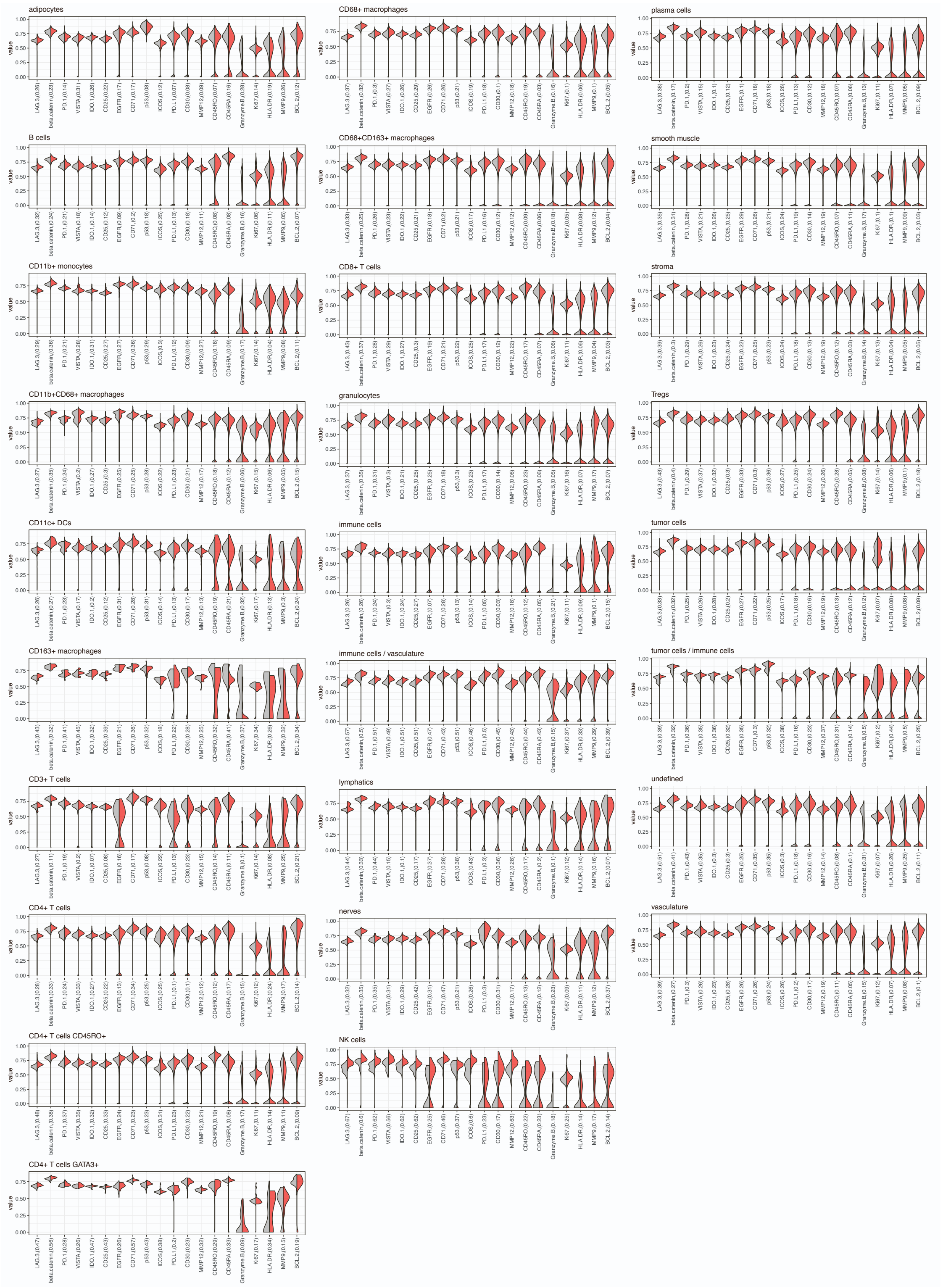

Fig. S7.

S3-CIMA global enrichment analysis at k =50, Density of functional marker expression showing greatest differential abundance in terms of the Kolmogorov–Smirnov two-sample test between the selected and non-selected cell subsets per cell types.

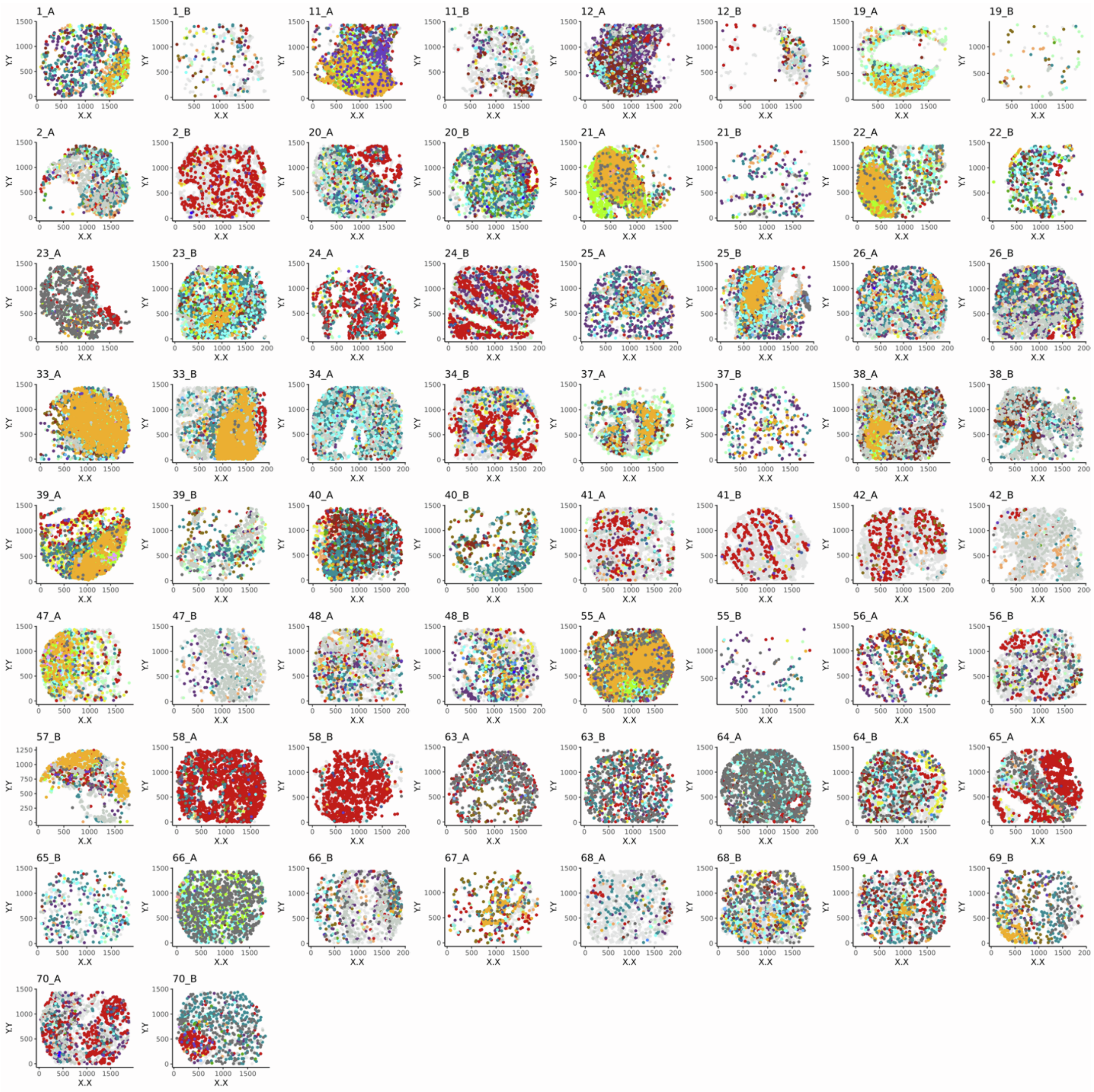

Fig. S8.

S^3-CIMA global enrichment analysis at k =30, Selected cells (colored by cell type) are mapped back to the corresponding patient CODEX images in both CLR group.

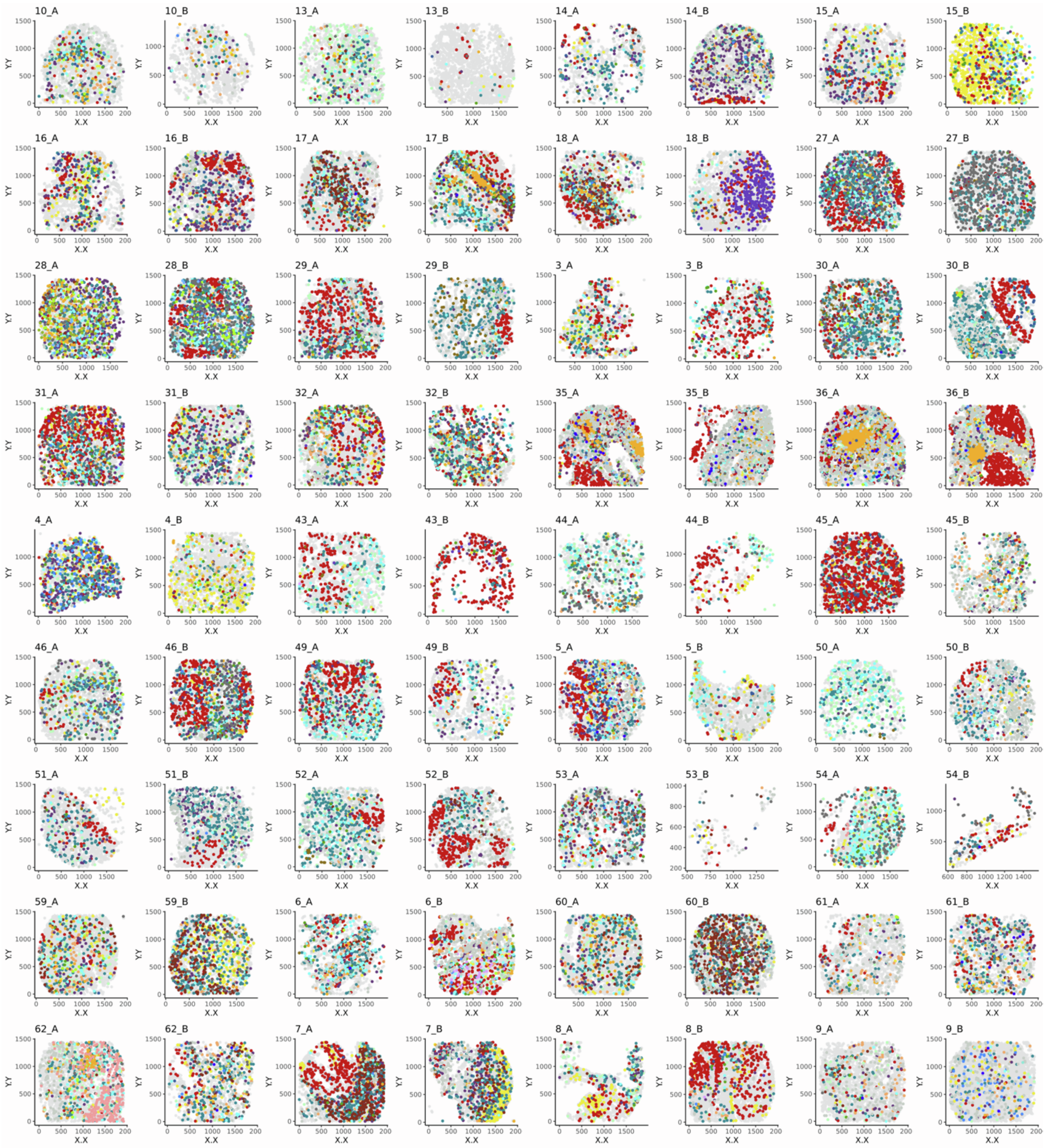

Fig. S9.

S^3-CIMA global enrichment analysis at k =30, Selected cells (colored by cell type) are mapped back to the corresponding patient CODEX images in both DII group.

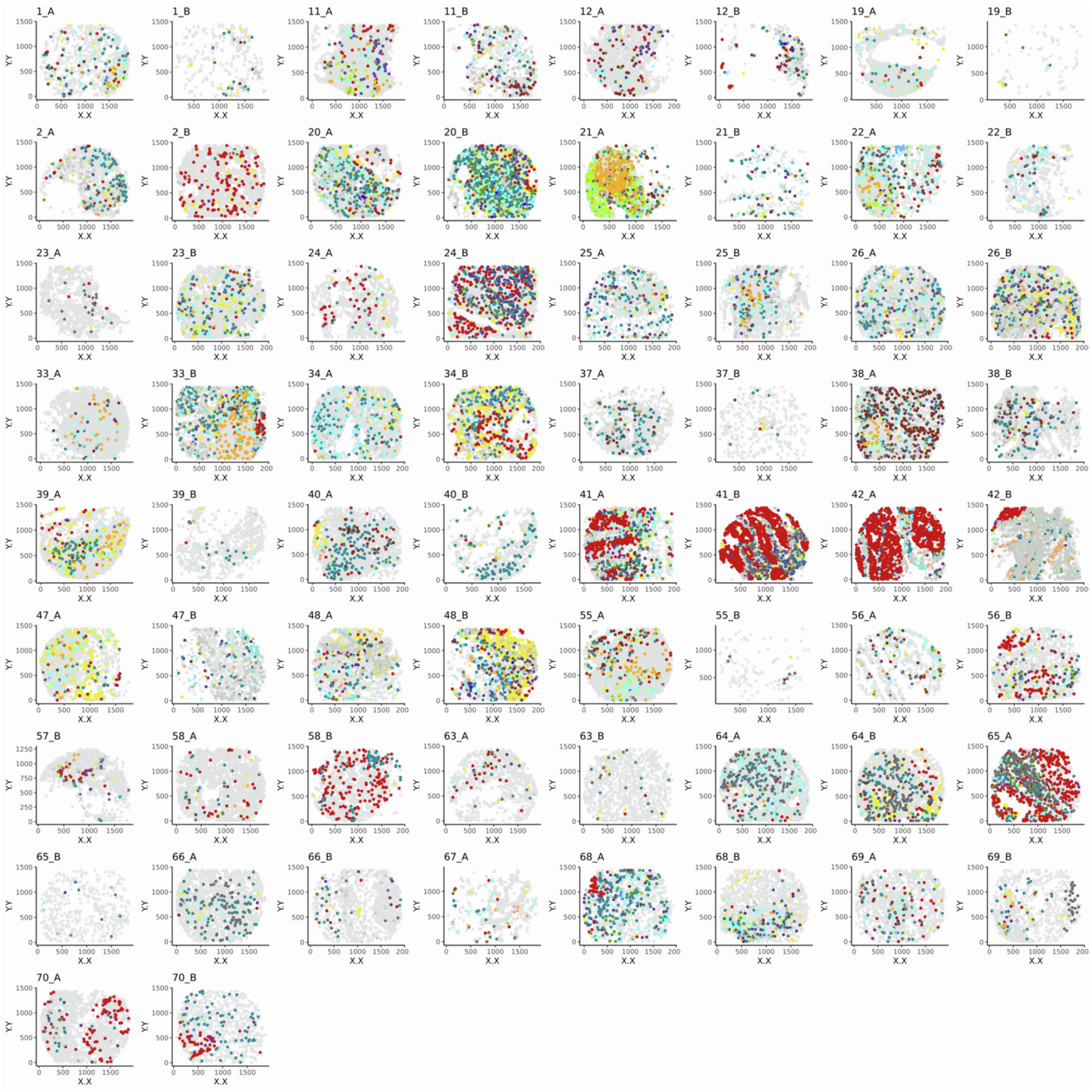

Fig. S10.

S^3-CIMA global enrichment analysis at k =50, Selected cells (colored by cell type) are mapped back to the corresponding patient CODEX images in both CLR group.

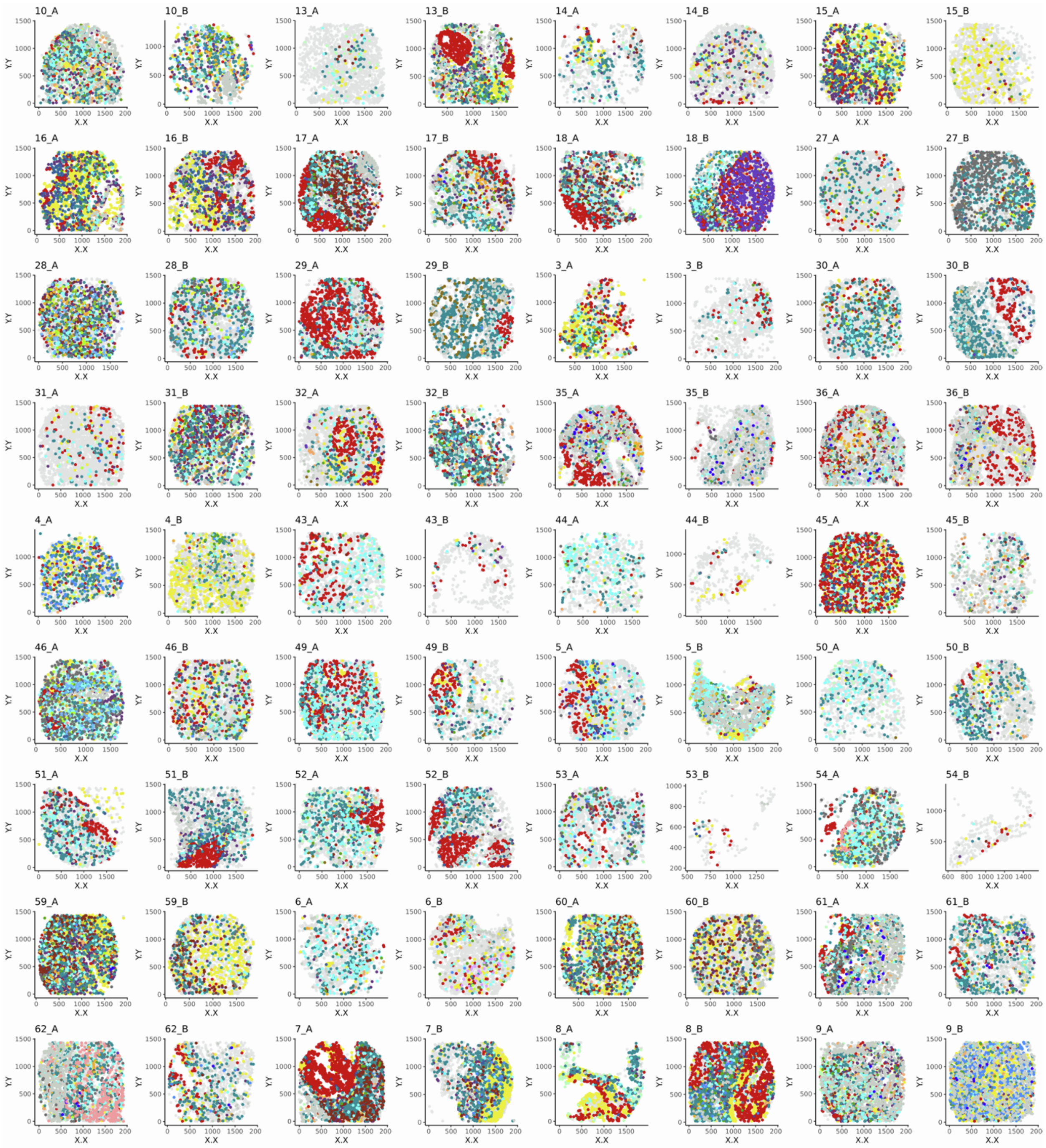

Fig. S11.

S^3-CIMA global enrichment analysis at k =50, Selected cells (colored by cell type) are mapped back to the corresponding patient CODEX images in both DII group.

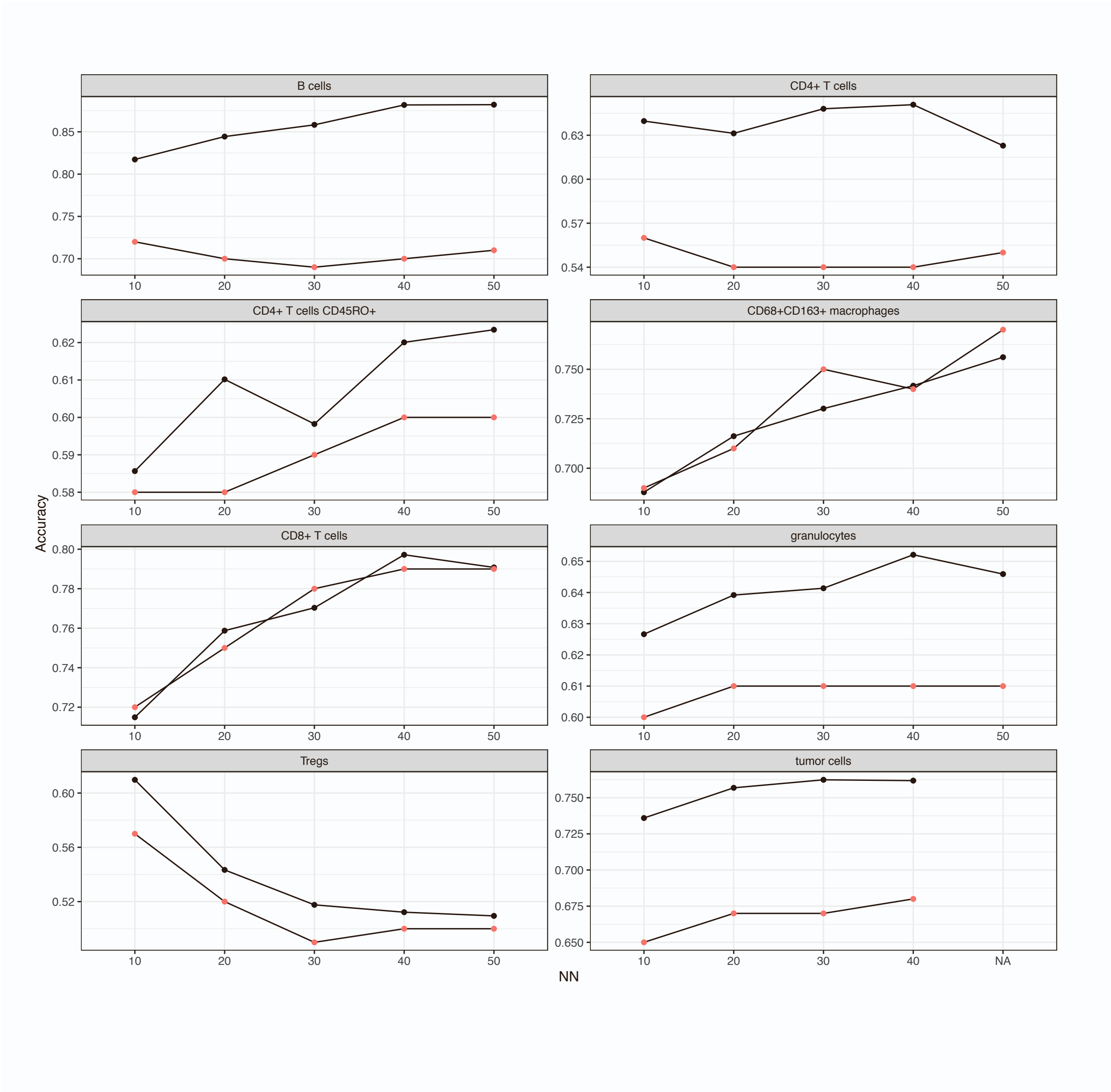

Fig. S12.

S^3-CIMA anchor based spatial enrichment analysis classification performance (test (red) and train (black) accuracy) across different cell neighborhood sizes (10 to 50).

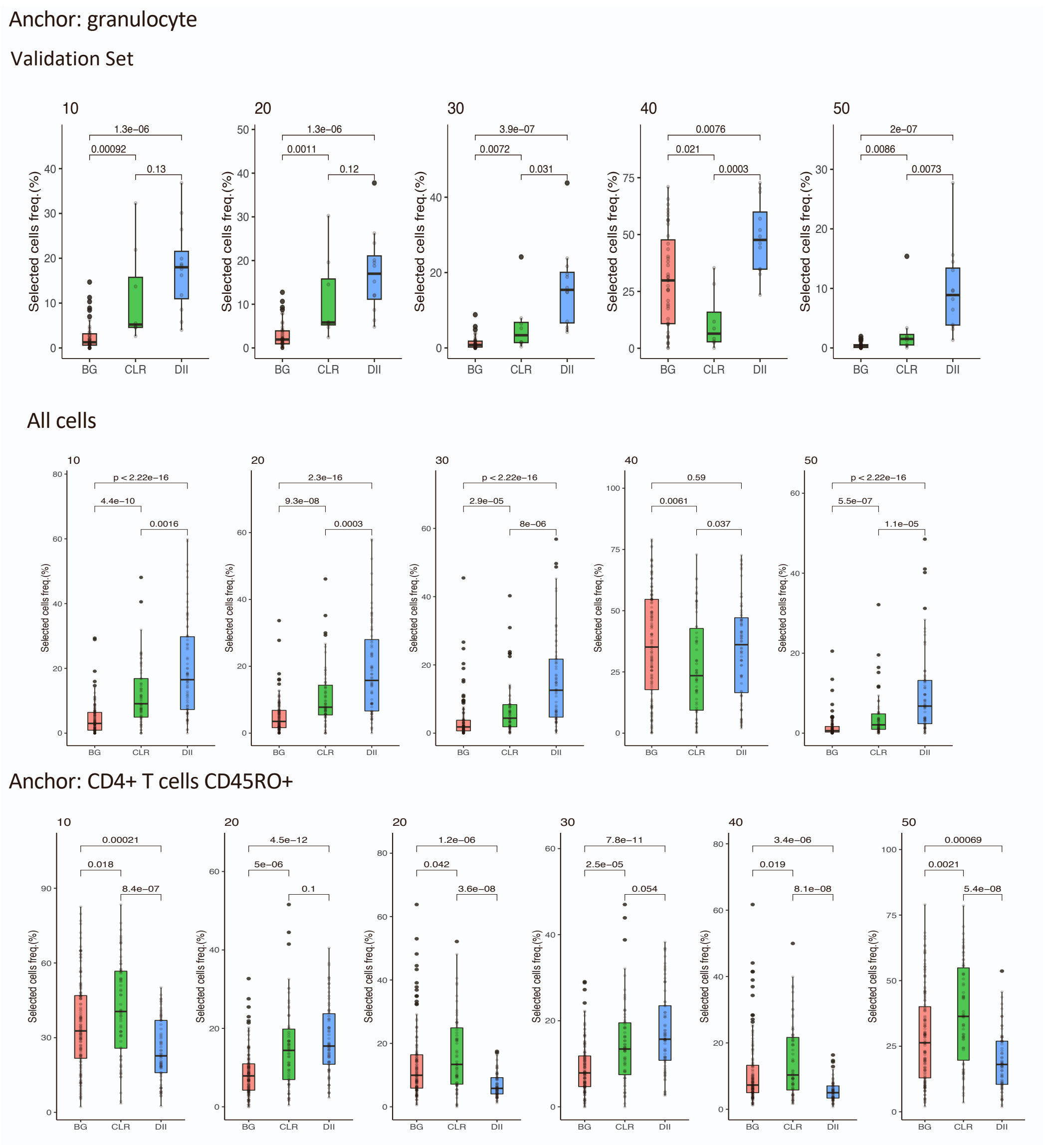

Fig. S13.

Boxplots of selected cell type frequency of two CRC groups using S^3-CIMA anchor based spatial enrichment analysis with granulocyte and CD4+ T cells CD45RO+ as the anchor.

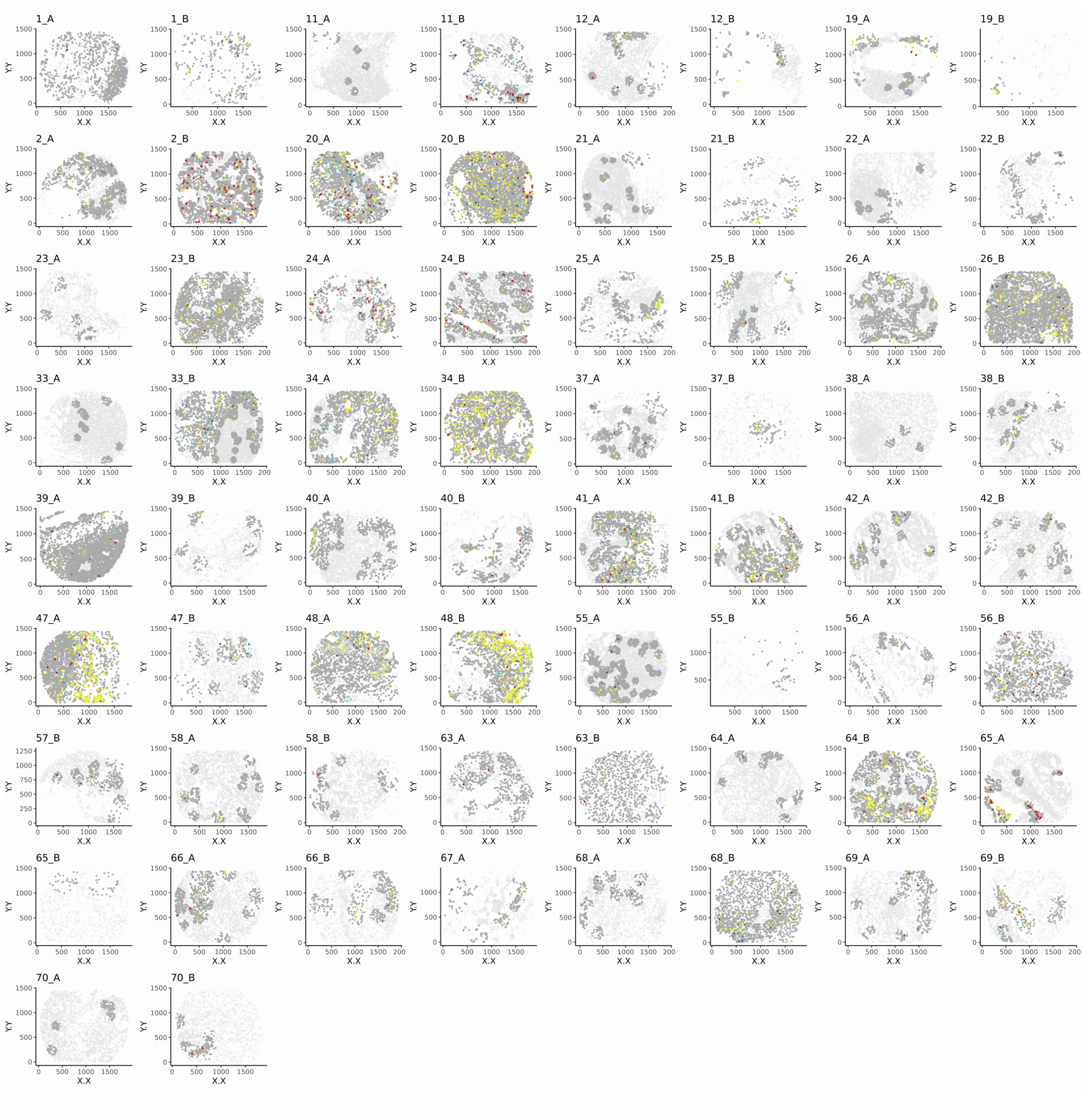

Fig. S14.

Selected cells (colored by cell type) using S^3-CIMA anchor based spatial enrichment analysis with granulocyte as the anchor mapped back to the corresponding patient CODEX images in the **CLR** group.

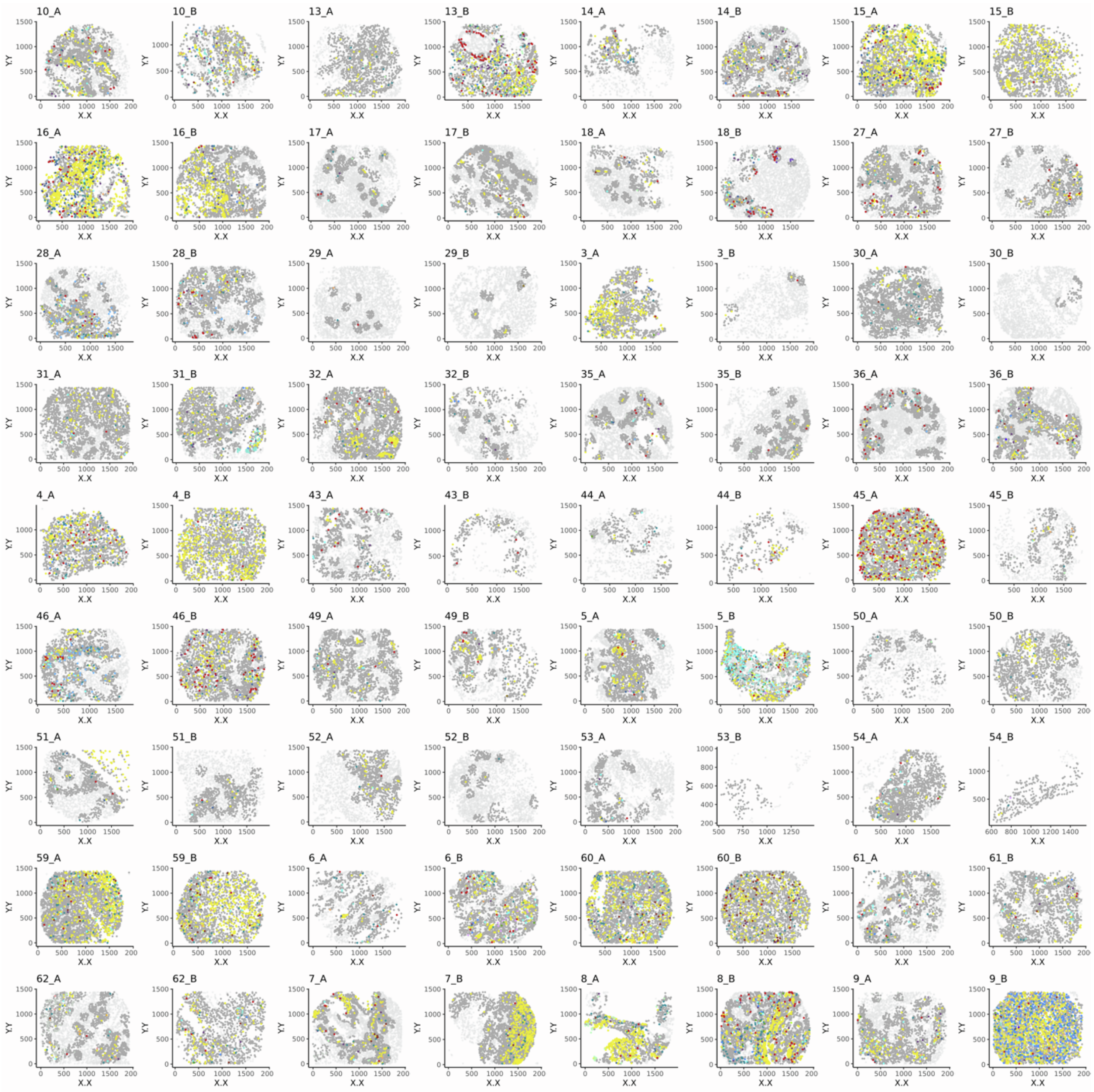

Fig. S15.

Selected cells (colored by cell type) using S[3]-CIMA anchor based spatial enrichment analysis with granulocyte as the anchor mapped back to the corresponding patient CODEX images in the **DII** group.

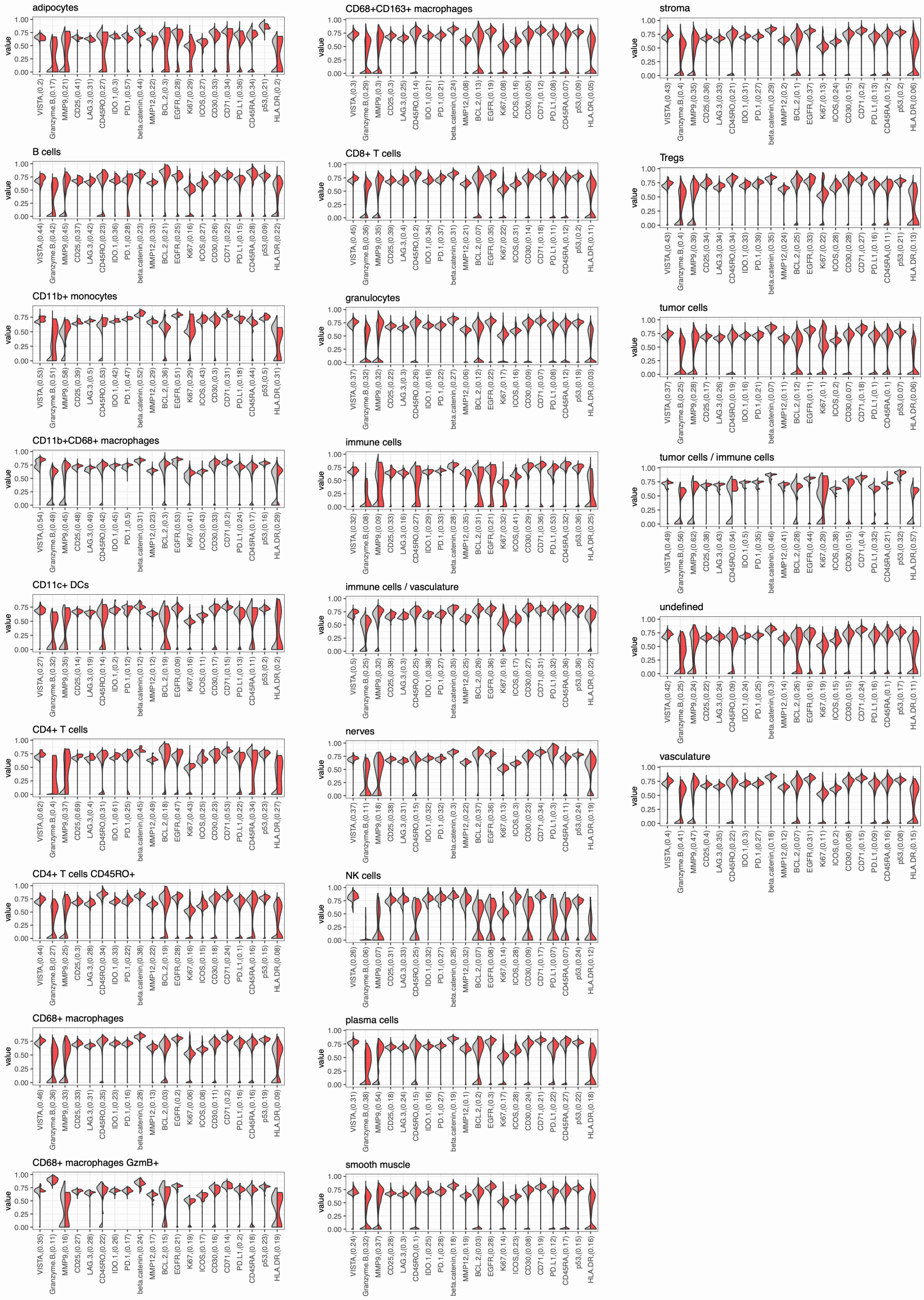

Fig. S16.

S^3-CIMA local enrichment analysis at k =30 granulocyte as the anchor, Density of functional marker expression showing differential abundance (KS two-sample test) between the selected and non-selected cells per cell types.

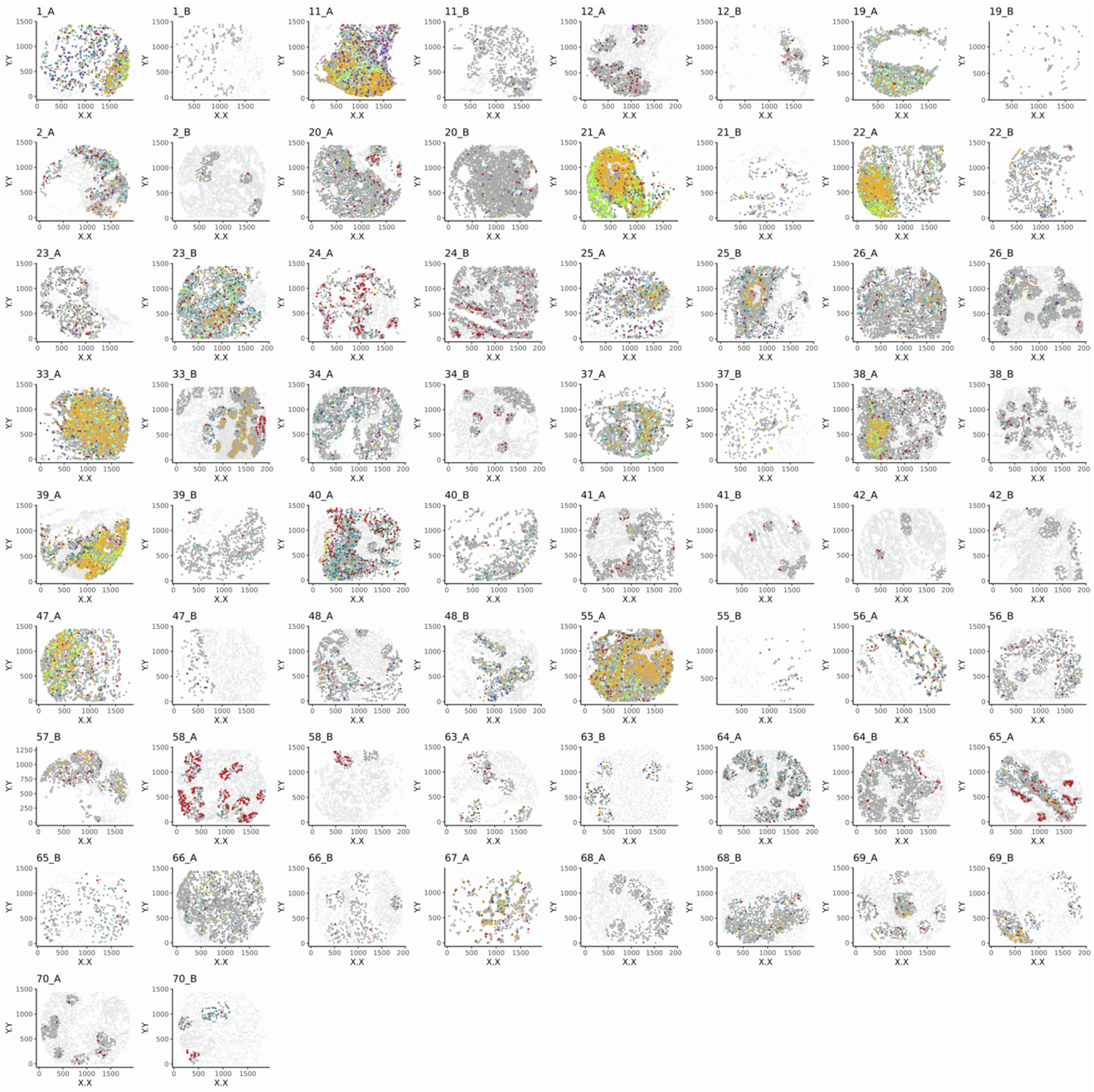

Fig. S17.

Selected cells (colored by cell type) using S^3-CIMA anchor based spatial enrichment analysis with CD4+ T cells CD45RO+ as the anchor mapped back to the corresponding patient CODEX images in the **CLR** group.

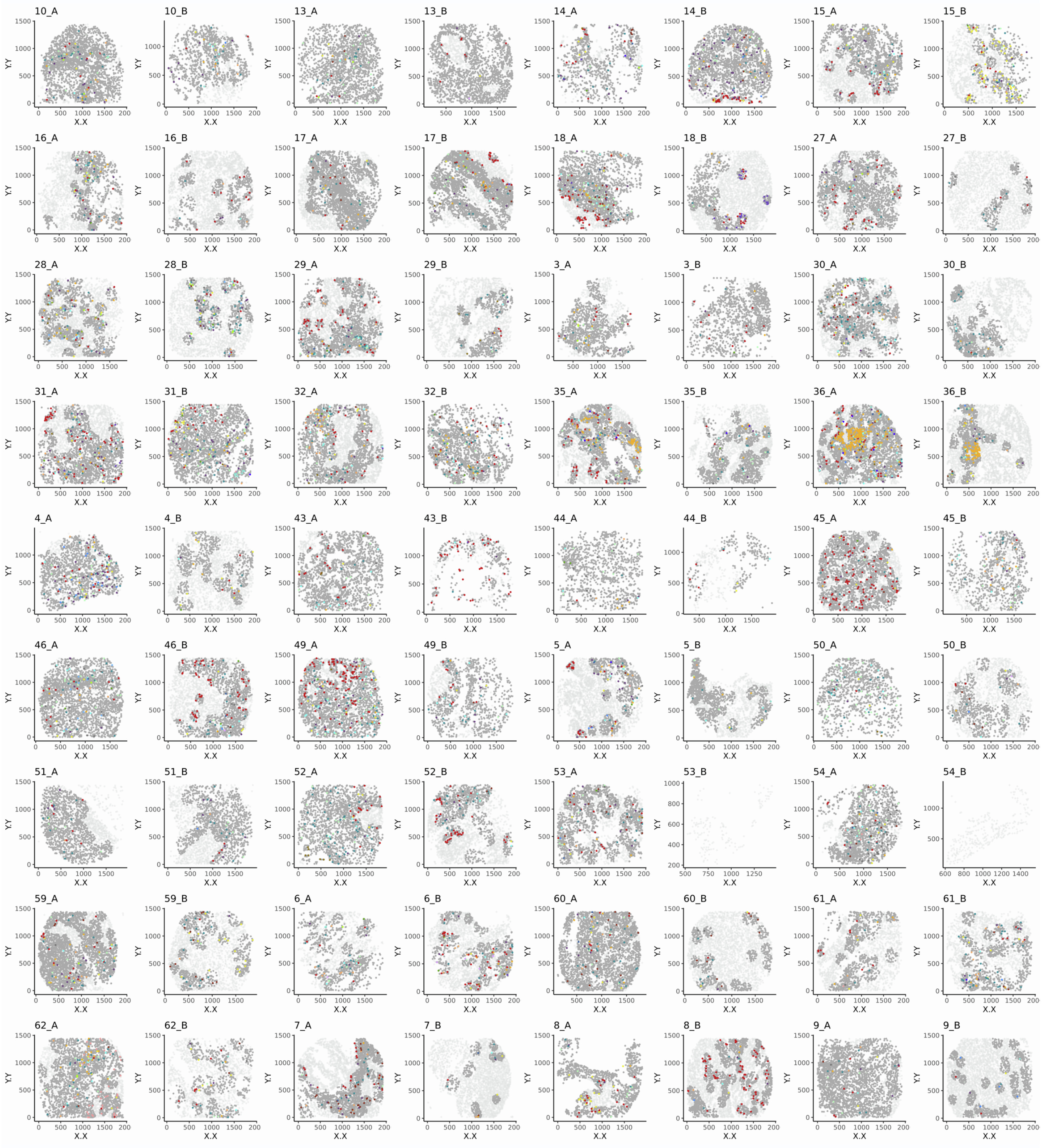

Fig. S18.

Selected cells (colored by cell type) using S^3-CIMA anchor based spatial enrichment analysis with CD4+ T cells CD45RO+ as the anchor mapped back to the corresponding patient CODEX images in the **DII** group.

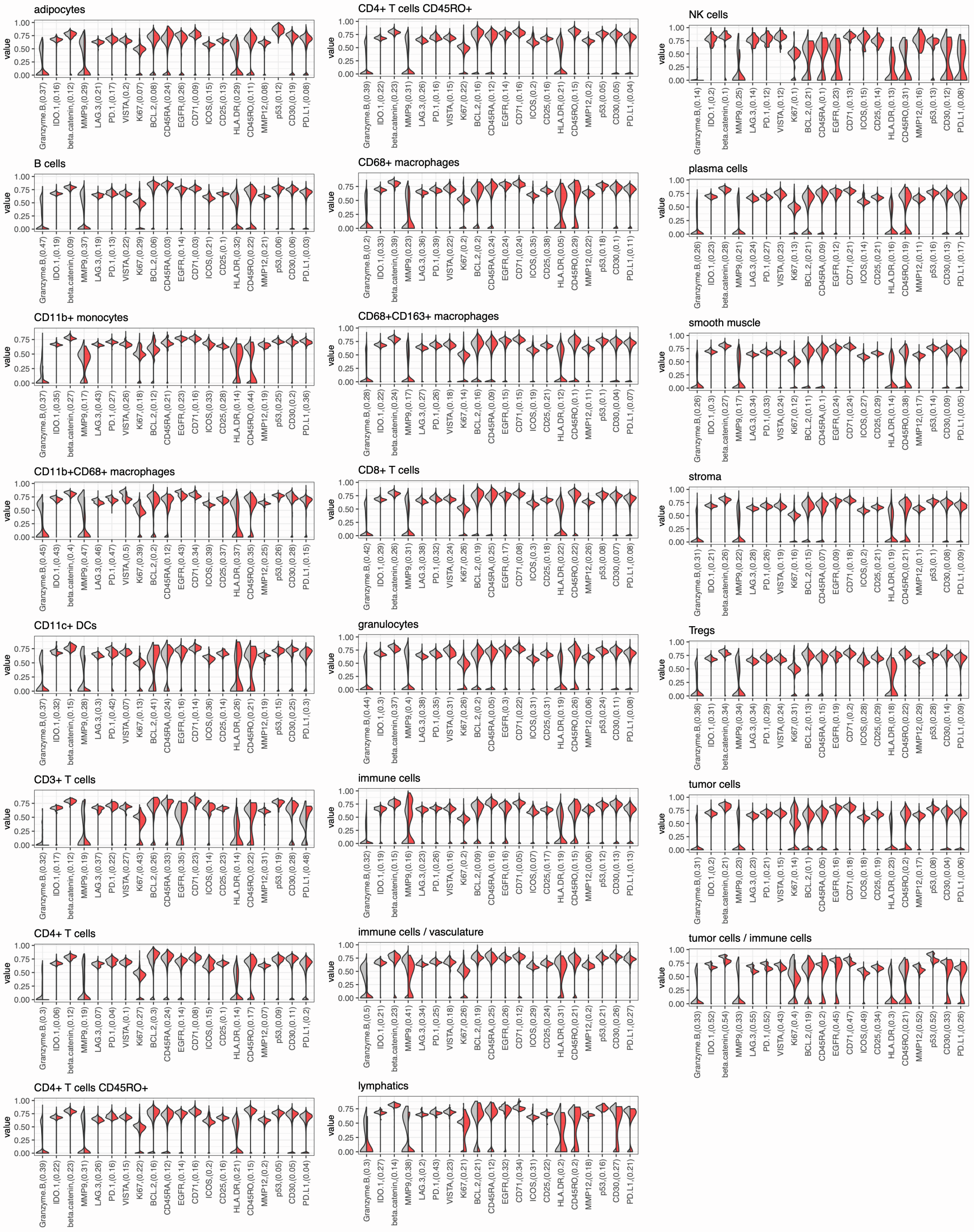

Fig. S19.

S^3-CIMA local enrichment analysis at k =40, CD4+ T cells CD45RO+ as the anchor, Density of functional marker expression showing differential abundance (KS two-sample test) between the selected and non-selected cells per cell types.

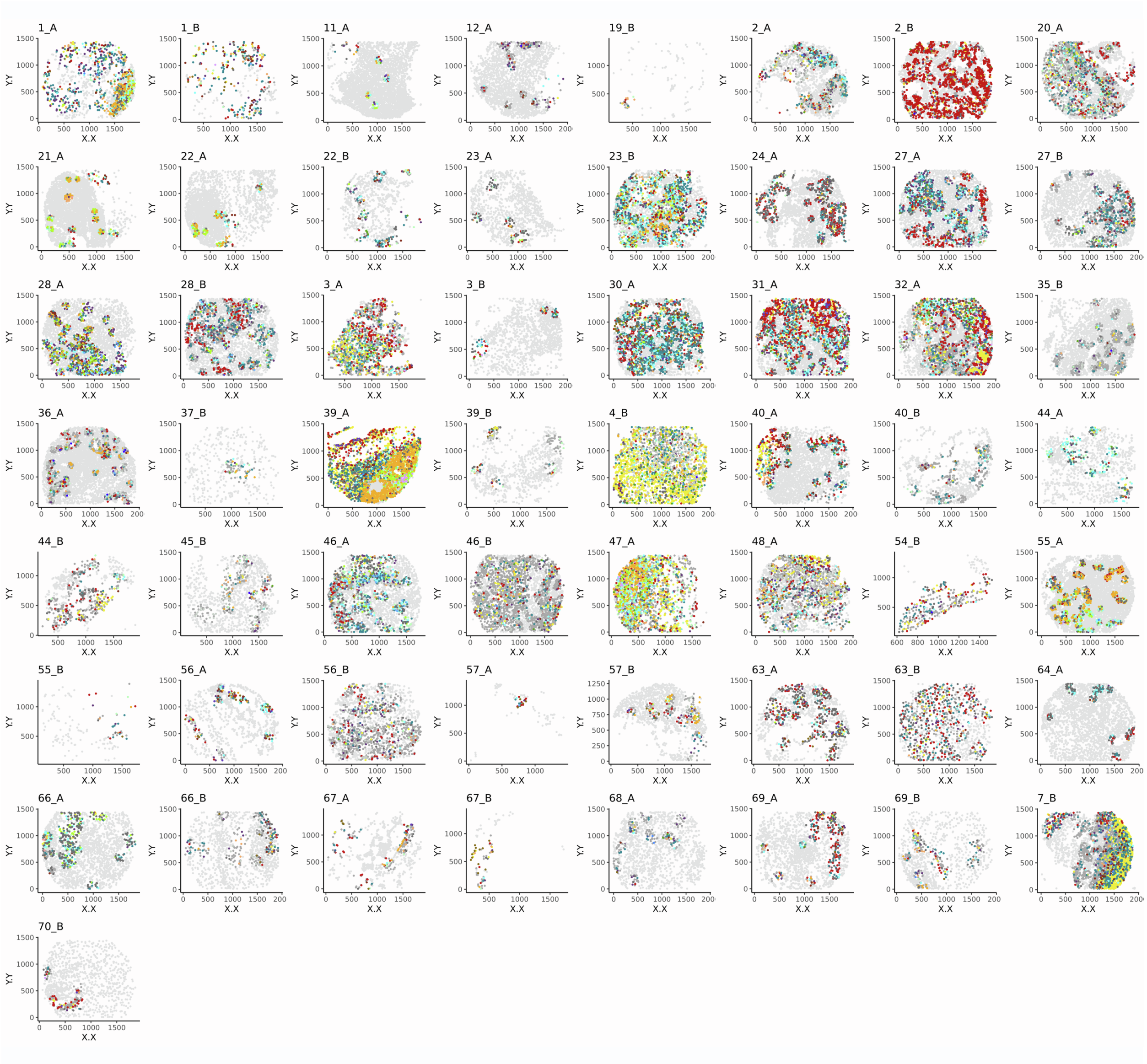

Fig. S20.

Selected cells (colored by cell type) using S[3]-CIMA functional spatial enrichment analysis with granulocytes as the anchor and EGFR marker expression as the phenotype mapped back to the corresponding patient CODEX images in the **EGFR low** group. (Filter 1).

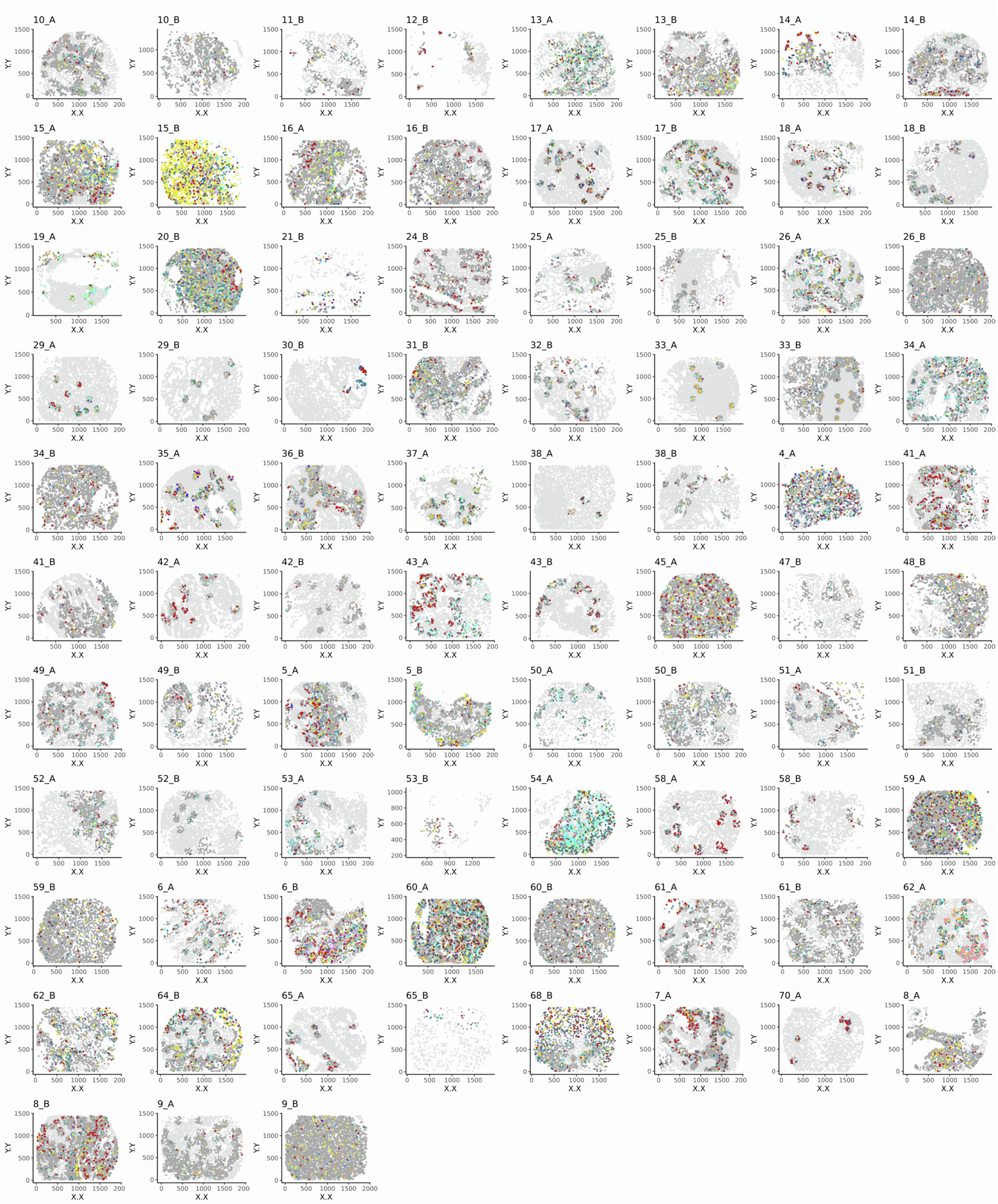

Fig. S21.

Selected cells (colored by cell type) using S[3]-CIMA functional spatial enrichment analysis with granulocytes as the anchor and EGFR marker expression as the phenotype mapped back to the corresponding patient CODEX images in the **EGFR high** group. (Filter 1).

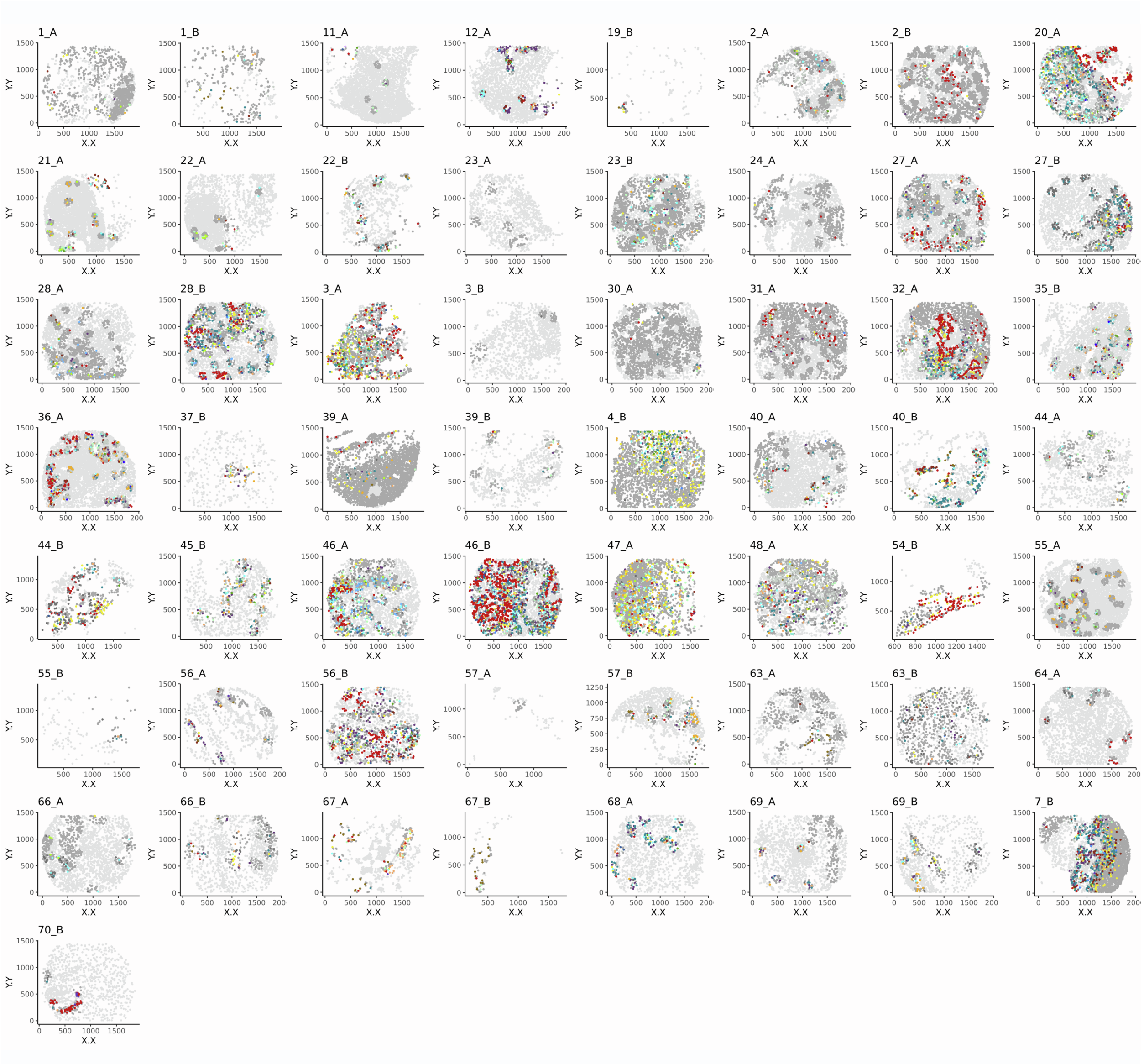

Fig. S22.

Selected cells (colored by cell type) using S^3-CIMA functional spatial enrichment analysis with granulocytes as the anchor and EGFR marker expression as the phenotype mapped back to the corresponding patient CODEX images in the **EGFR low** group. (Filter 2) .

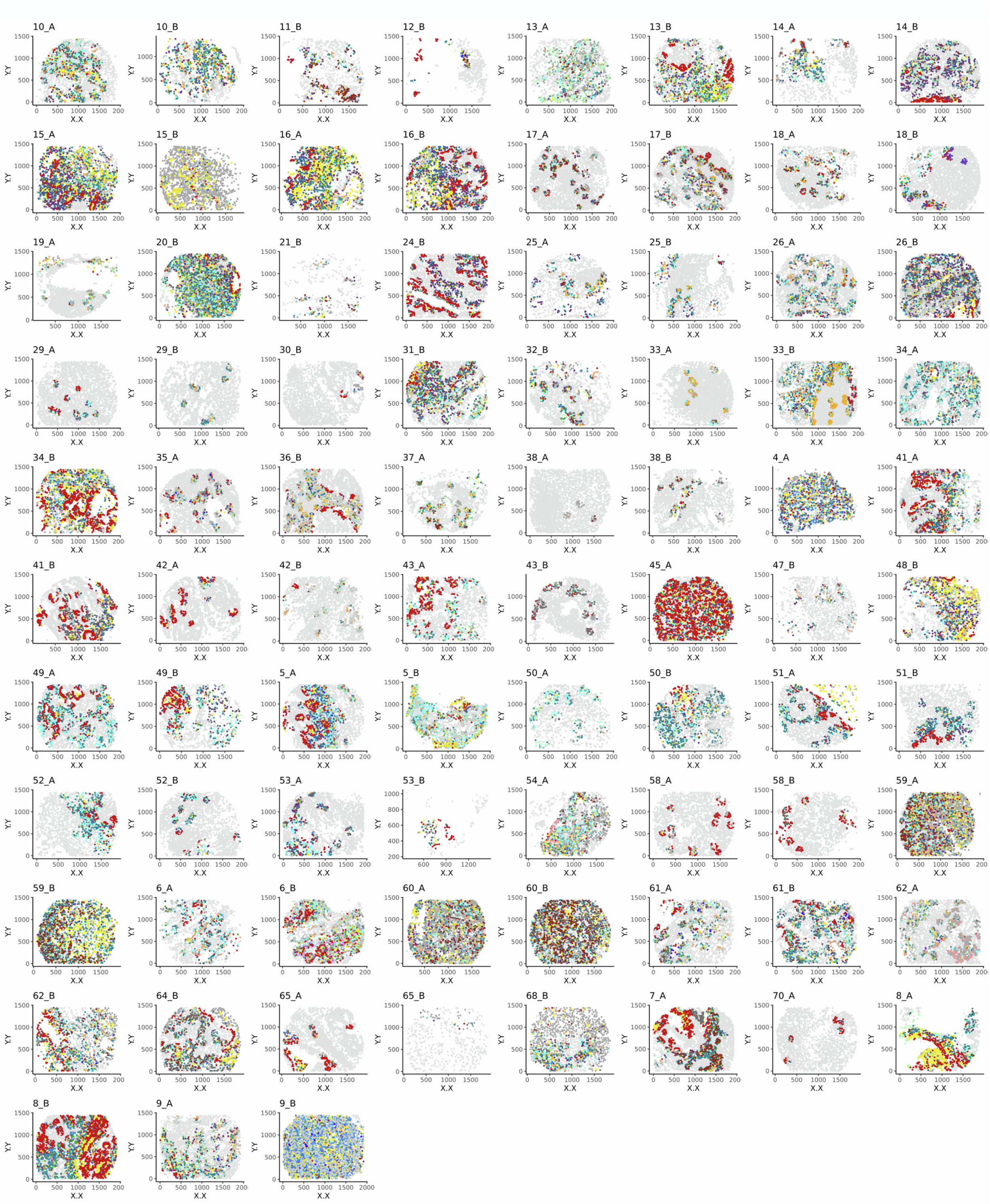

Fig. S23.

Selected cells (colored by cell type) using S^3-CIMA functional spatial enrichment analysis with granulocytes as the anchor and EGFR marker expression as the phenotype mapped back to the corresponding patient CODEX images in the **EGFR high** group. (Filter 2).

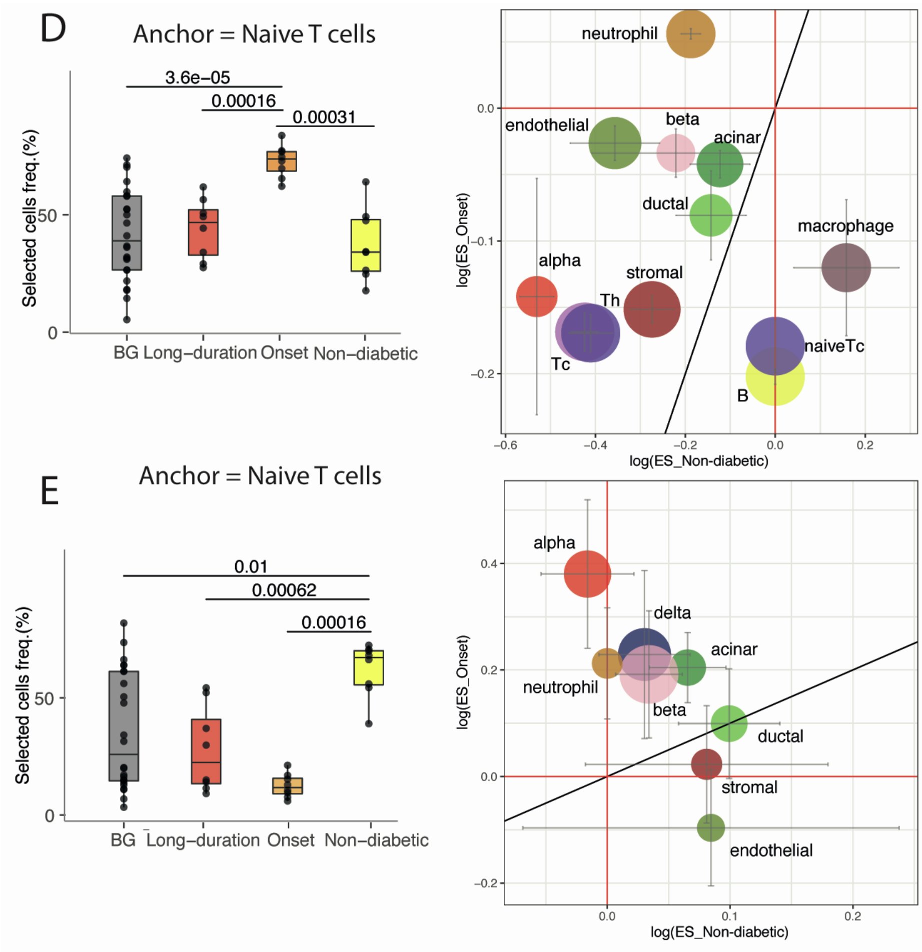

Fig. S24.

S^3-CIMA anchor based spatial enrichment analysis with Naïve T cells as the anchor.

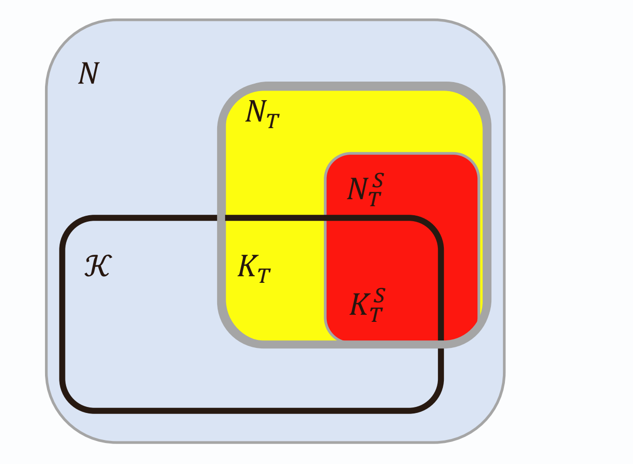

Fig. S25.
The enrichment score (ES) quantifies the association or exclusion of selected cells of specific cell type in the spatial proximity of the anchor cell. The variables N is the number of all cells in the image, K is the number of all cells in the nearest neighborhood of the anchor cell, N_T is the number of all cells of cell type T in the image, K_T is the number of cell type T in the nearest neighborhood of the anchor cell, N_T^S is the number of all selected cells of type T and K_T^S is the number of selected cell type T in the nearest neighborhood of the anchor cell.

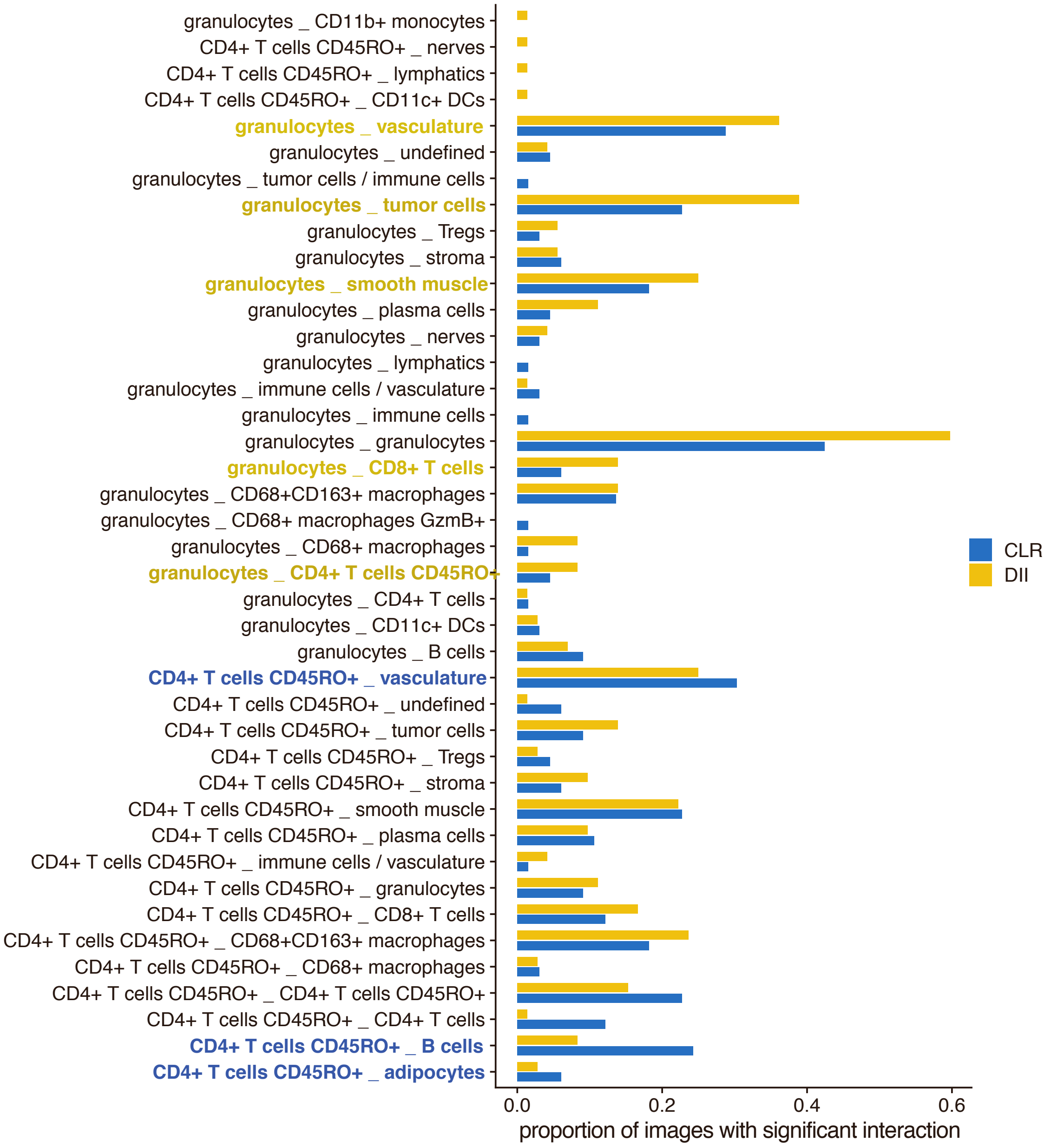

Fig. S26.
We applied the unsupervised histoCAT analysis to the data of the CRC cohort. The method enables unsupervised spatial interrogation of cell–cell interactions. The neighborhood analysis in histoCAT examines if a certain cell type is located significantly closer to another cell type in each tissue image than expected by chance using two individual one-tailed permutation tests. For each specific cell–cell interaction, we calculated the proportion of tissue images in which that interaction is significant per survival group and highlighted an interaction if the proportion is high in one of the groups.

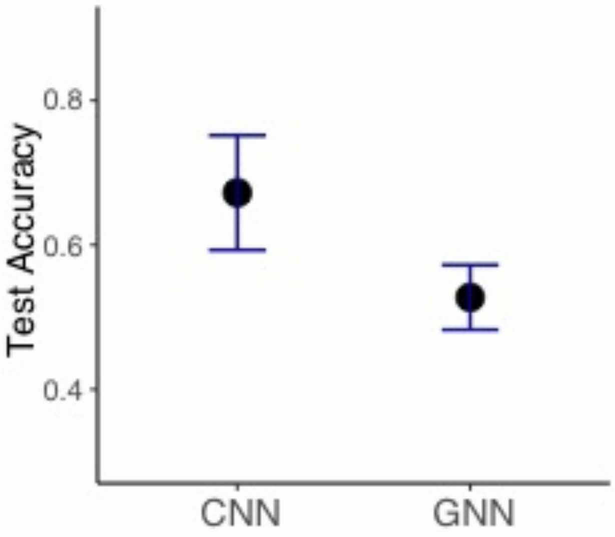

Fig. S27.
Comparing the prediction accuracy between the Graph Neural Networks (GNN) and the CNN model for global spatial enrichment analysis. The GNN model consists of a ReLu activation function, a pooling layer, and a classification output. It takes into account the marker expression of the cells in each multi-cell input to construct the neighborhood graph. To compare its performance with the CNN model, we used an identical multi-cell input of the global spatial enrichment analysis of S3-CIMA (i.e., k-NN cells of random anchor cells) to construct the neighborhood graph, while maintaining the same training and test sets. The standard GNN yielded lower classification accuracy.
