# Supplementary material for: S3-CIMA: Supervised spatial single-cell image analysis for identifying disease-associated cell-type compositions in tissue
Source: Patterns (N Y). 2023 Aug 17;4(9):100829. doi: 10.1016/j.patter.2023.100829 (PMC10500029; doi:10.1016/j.patter.2023.100829)
Supplement: Document S2. Article plus supplemental information [file mmc2.pdf]

Patterns

Article

S^3-CIMA: Supervised spatial single-cell image analysis for identifying disease-associated cell-type compositions in tissue

Graphical abstract

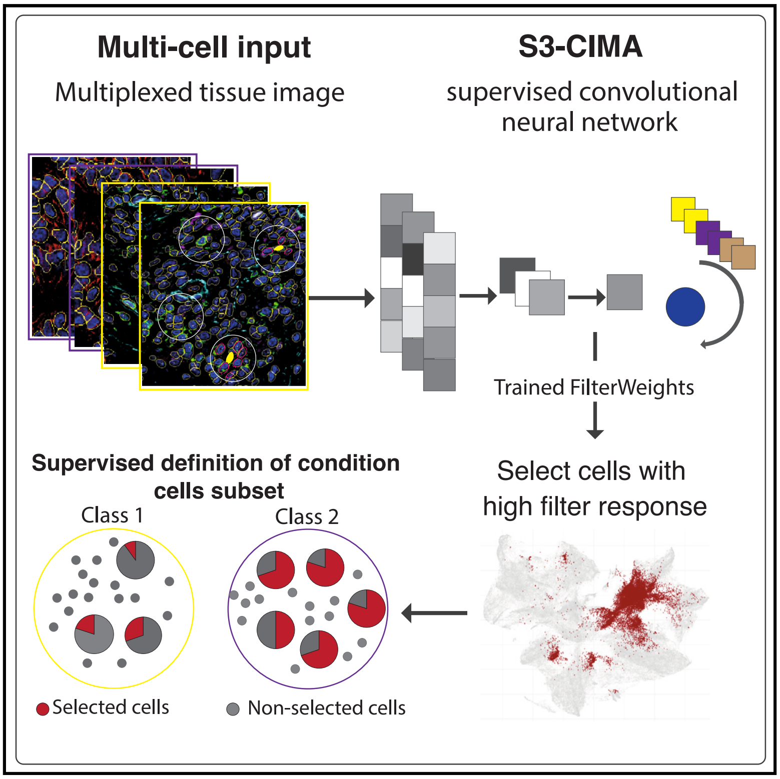

Highlights

- S^3-CIMA, a supervised convolutional neural network model for single-cell image analysis
- S^3-CIMA can be used to discover disease-associated spatial cellular interactions
- Application reveals cellular associations with colorectal cancer outcome

Authors

Sepideh Babaei, Jonathan Christ, Vivek Sehra, ..., Kilian Wistuba-Hamprecht, Christian Schürch, Manfred Claassen

Correspondence

manfred.claassen@med.uni-tuebingen.de

In brief

The tissue microenvironment is a dynamic system consisting of various cell types, arranged spatially to create emergent tissue properties, in health and disease. Here, the authors present S^3-CIMA, a weakly supervised convolutional neural network model that can be used to identify disease-specific microenvironment compositions from single-cell imaging data. The model reveals cellular interactions and spatial organization in tumor microenvironments of colorectal cancer, pancreatic tissue in type 1 diabetes, and cellular signaling activity.

Babaei et al., 2023, Patterns *4*, 100829
September 8, 2023 © 2023 The Author(s).
https://doi.org/10.1016/j.patter.2023.100829

CellPress

Patterns

CellPress
OPEN ACCESS

Article

S^3-CIMA: Supervised spatial single-cell image analysis for identifying disease-associated cell-type compositions in tissue

Sepideh Babaei,[1,6] Jonathan Christ,[2] Vivek Sehra,[1,3,6] Ahmad Makky,[4] Mohammed Zidane,[4]
Kilian Wistuba-Hamprecht,[1,5,6] Christian Schürch,[4] and Manfred Claassen[1,3,6,7,8,*]
[1]Department of Internal Medicine I, University Hospital Tübingen, Tübingen, Germany
[2]Department of Physics, University of Vienna, Vienna, Austria
[3]Department of Computer Science, University of Tübingen, Tübingen, Germany
[4]Department of Pathology and Neuropathology, University Hospital and Comprehensive Cancer Center Tübingen, Tübingen, Germany
[5]Department of Immunology, Institute of Cell Biology, University Hospital Tübingen, Tübingen, Germany
[6]M3 Research Center, University Hospital Tübingen, Tübingen, Germany
[7]Institute for Bioinformatics and Medical Informatics, University of Tübingen, Tübingen, Germany
[8]Lead contact
*Correspondence: manfred.claassen@med.uni-tuebingen.de
https://doi.org/10.1016/j.patter.2023.100829

THE BIGGER PICTURE The spatial organization of cell types within a tissue is an important determinant of physiological as well as pathological processes, including cancer and autoimmune diseases. While new single-cell imaging technologies are enabling the study of tissues in detail, it remains challenging to derive models that describe the spatial organization of cells and link this information to disease conditions. This work describes a supervised convolutional neural-network-based method that can be used to bridge this gap. For instance, this model allows the identification of local organizations of cell types in colorectal cancer tissues that are associated with poor disease outcomes. Such findings can be used to generate new mechanistic hypotheses, potentially leading to new insights into health and disease. Methods like this one may help the development of new personalized interventions that favorably influence disease states, e.g., novel immunotherapies to deplete inhibitory cell types.

1 2 3 4 5 **Development/Pre-production:** Data science output has been rolled out/validated across multiple domains/problems

SUMMARY

The spatial organization of various cell types within the tissue microenvironment is a key element for the formation of physiological and pathological processes, including cancer and autoimmune diseases. Here, we present S^3-CIMA, a weakly supervised convolutional neural network model that enables the detection of disease-specific microenvironment compositions from high-dimensional proteomic imaging data. We demonstrate the utility of this approach by determining cancer outcome- and cellular-signaling-specific spatial cell-state compositions in highly multiplexed fluorescence microscopy data of the tumor microenvironment in colorectal cancer. Moreover, we use S^3-CIMA to identify disease-onset-specific changes of the pancreatic tissue microenvironment in type 1 diabetes using imaging mass-cytometry data. We evaluated S^3-CIMA as a powerful tool to discover novel disease-associated spatial cellular interactions from currently available and future spatial biology datasets.

INTRODUCTION

The tissue microenvironment (TME) constitutes a spatially organized, dynamic, and complex system of multiple cell types conferring emergent tissue properties in health and disease. Lymph nodes constitute a seminal example of how spatial organization of cell-type composition in the TME confers emergent properties in terms of orchestrating the immune response to

This is an open access article under the CC BY license (http://creativecommons.org/licenses/by/4.0/).

pathogens.[1] In particular, dendritic cells and T cells are spatially matched in the lymph node's T cell zone to increase the efficiency of adaptive immunity.[2,3] Until recently, it has been difficult to study such structures due to the technical requirement to resolve both spatial as well as high dimensional molecular profiles at the single-cell level. Moreover, the subsequent requirements to—typically computationally—integrate and interpret the resulting data to the end of defining descriptions or models of the TME, and their disease-associated aberrations have been challenging.

This issue has been addressed technically with the development of high-dimensional proteomic, transcriptomic, and epigenomic methods;[4,5] single-cell spatial biology technologies; covering multiplexed fluorescence *in situ* hybridization (FISH); imaging mass cytometry (IMC);[6] and multiplexed microscopy such as cyclic immunofluorescence (CyCIF)[7] and co-detection by indexing (CODEX).[8,9] These approaches enable joint measurement of dozens of features in FISH, up to hundreds in multiplexed microscopy, and tens of thousands in spot-based spatial transcriptomics. Different resolutions, from local tissue spots as in spatial transcriptomics (e.g., high-definition spatial transcriptomics,[10] Visium,[11] deterministic barcoding in tissue sequencing [DBit-seq][12]) to subcellular resolution (e.g., FISH, IMC, CyCif, CODEX) are possible. Further, dedicated image processing, segmentation, and registration approaches have been developed to process and visualize the resulting data and quantify their respective signal intensities and distributions.[13–15]

The resulting data lend themselves to infer a description of TME arrangements, specifically in terms of cell-type/state frequencies. Inferring the TME is a machine learning task that encompasses two distinct approaches: unsupervised inference, which defines TME arrangements solely from the spatial single-cell data, and supervised inference, which defines TME characteristics associated with external cues, such as disease state. While the former approaches allow the definition of TME cell-type composition, the latter approaches take an additional step to identify differential cell-type composition across conditions, potentially revealing novel and previously unrecognized cell subtypes. So far, only unsupervised approaches of the first kind have been reported, and supervised approaches of the second kind are lacking.

Approaches of the first kind include Histology Topography Cytometry Analysis Toolbox (histoCAT),[16] enabling unsupervised spatial interrogation of cell-cell interactions in IMC data. The neighborhood analysis in histoCAT examines if a certain cell type is located significantly closer to another cell type than expected by chance using two individual one-tailed permutation tests. Spatial transcriptomics learn (stLearn) was developed to calculate cell-cell interactions based on the unsupervised clustering of morphological similarity of spatial transcriptomics data.[17] Briefly, stLearn utilizes unsupervised clustering to group similar spots into clusters (i.e., spatial morphological gene expression), and then significant cell-cell interactions are detected by a permutation approach. The tissue location hotspots are determined as locations in the tissue where there are both high interaction activity and diverse cell-type co-localization. The Giotto analyzer and viewer[18] consists of tools to process and visualize spatial transcriptomics and proteomics expression data. The toolbox introduced new methods to identify a feature (gene or protein) that constructs a coherent spatial pattern based on unsupervised clustering and statistical enrichment of spatial network neighbors. In sum, these approaches often utilize nearest neighbor and other statistical approaches to identify relationships between different cell types and their TME based on an unsupervised strategy.[19,20]

The above-mentioned methods are TME inference approaches of the first kind—as defined above—i.e., they are all unsupervised learning approaches that allow to define TME prototypes as not allowing to infer TME differences associated with and possibly explaining external cues, such as disease state. Supervised learning approaches are required to overcome this limitation. Such approaches allow for identifying differences of TME models in comparative study settings, aiming at identifying differences of the same TME prototype across conditions, such as disease state or patient outcome. Supervised learning approaches enabling the detection of disease-associated TME compositions are so far lacking.

To close this gap, we present supervised spatial single-cell image analysis (S^3-CIMA) for the identification of disease-associated cell-type compositions in tissue microenvironments. S^3-CIMA is a weakly supervised approach that leverages a single-layer convolutional neural network (CNN) architecture.[21] We demonstrate the utility of S^3-CIMA by identifying outcome-specific cell-state compositions from a CODEX-based study of the colorectal cancer (CRC) TME. Specifically, we applied the method to a cohort of CRC patients to identify high-risk-specific spatial neighborhood cell-type compositions in the tumor.[20] Further, we use S^3-CIMA to identify disease-onset-specific changes of the pancreatic TME in type 1 diabetes (T1D) in an IMC study.[22] We expect that S^3-CIMA will enable the study of other diseases and spatial biology data types and will generally be valuable to identify novel disease-associated cellular interactions.

RESULTS

Weakly supervised learning of disease-associated TME compositions with S^3-CIMA

We aim at learning (disease) condition-associated TME composition from multiparametric and spatially resolved single-cell data. Specifically, we consider the notion of condition-associated TME compositions as the spatial local enrichment of specific cell subsets with respect to a condition, i.e., supervised spatial enrichment analysis (Figure 1A).

We distinguish three different types of supervised spatial enrichment analysis. In global spatial enrichment analysis, we aim at identifying which cell subsets are enriched in spatial neighborhoods across conditions, e.g., in CRC tumor tissues of disease manifestations with varying clinical outcomes. In local spatial enrichment analysis, we instead aim at identifying condition-specific cell subset enrichment in the proximity of an anchor cell type, e.g., tumor-infiltrating cytotoxic T cells in CRC patients with different clinical outcomes. In functional spatial enrichment analysis, we aim at identifying cell subsets enriched in the proximity of a specific functional activity of the tissue, e.g., local expression of a functional immune cell marker.

The input of S^3-CIMA is derived from high-dimensional *in situ* proteomic imaging data that have been processed up

CellPress
OPEN ACCESS

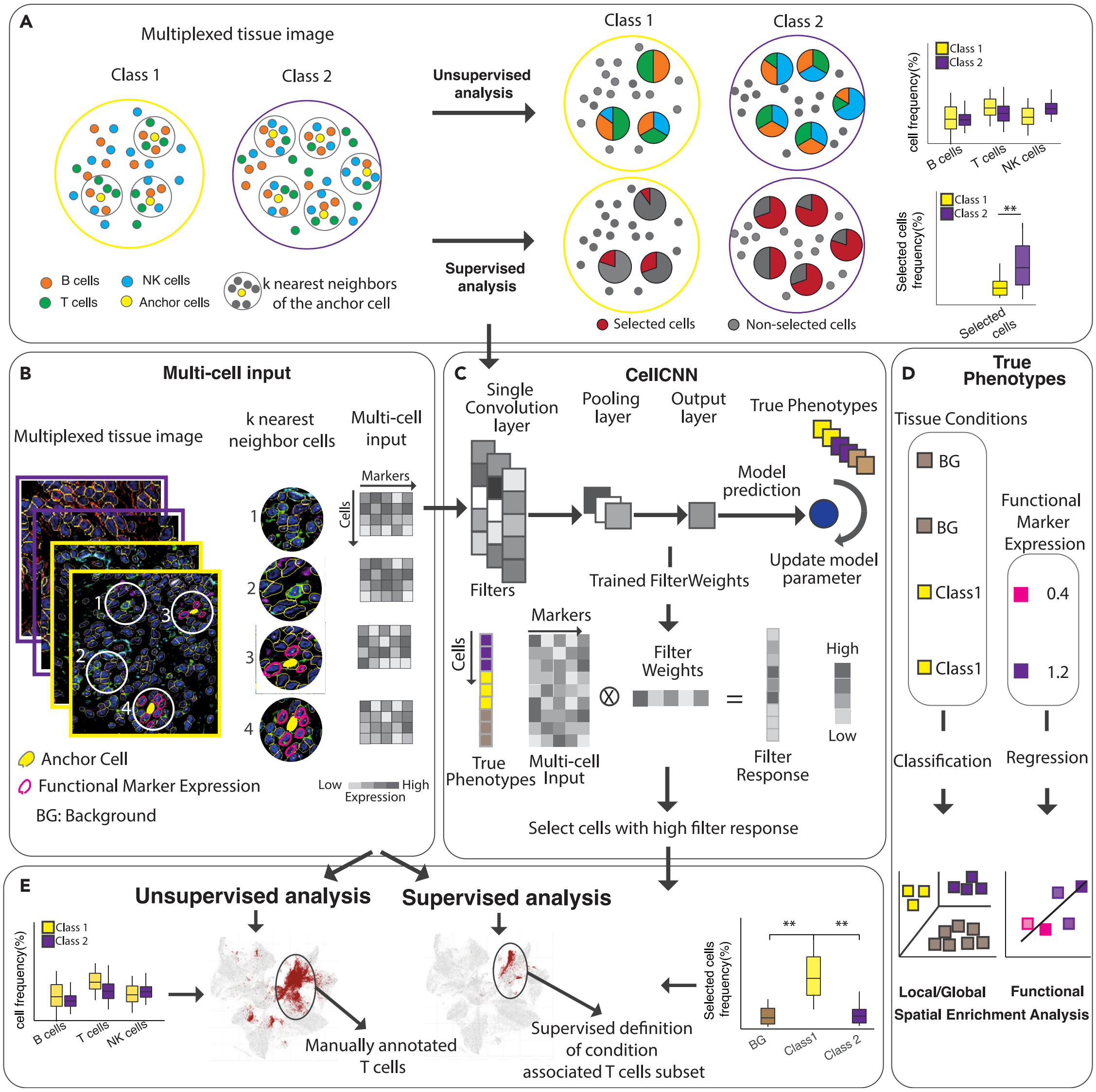

Figure 1. S^3-CIMA overview and CRC global spatial enrichment analysis

S^3-CIMA leverages a single-layer CNN architecture adopted from the CellCNN model.[23]

(A) Two types of TME definitions: unsupervised and supervised. Unsupervised approaches allow for defining cell type composition of the TME. Unsupervised approaches compare the frequency of conventional cell types across the conditions, while supervised approaches identify differential subsets of cell type composition across tissue conditions.

(B) The multi-cell input consists of k nearest neighbor cells around an anchor cell in a tissue image per phenotype label (i.e., class) and a set of marker expressions as the features.

(C) The model is a single-layer CNN in which node activities are computed by weighted sums over marker expression values of each cell. The pooling layer summarizes the filter response of all cells in a multi-cell input per each convolutional filter. The trained filter weights are obtained by optimizing the CNN weights using true labels of multi-cell inputs. For each cell in the image data, the filter response is calculated by scalar product of the cell marker expression and the CNN trained filter weights. The cells with relatively high filter response indicate cells with a distinct signature to manifest the class of the image. The significant difference of frequency of selected cell populations between labels shows that the CNN model learns the difference between the phenotype labels.

(D) Different types of supervised spatial enrichment analysis, including local spatial enrichment analysis for identifying which cell subsets are enriched in the proximity of an anchor cell type, functional spatial enrichment analysis for identifying cell subsets enriched in the proximity of a specific functional activity of the tissue.

(E) Two types of TME definitions: unsupervised and supervised. Unsupervised approaches allow for defining cell type composition of the TME; supervised approaches go a step further to identify differential cell type composition across tissue conditions, including condition-associated cell subtypes.

to single-cell segmentation, giving rise to a data matrix where every single cell (row) is associated with a profile of protein marker expression levels and spatial coordinates in the tissue image (columns) (Figure 1B). The input for S^3-CIMA are multi-cell inputs of spatially proximal cells, i.e., k-nearest cell neighborhoods (k-NNs) around an anchor position (e.g., position of

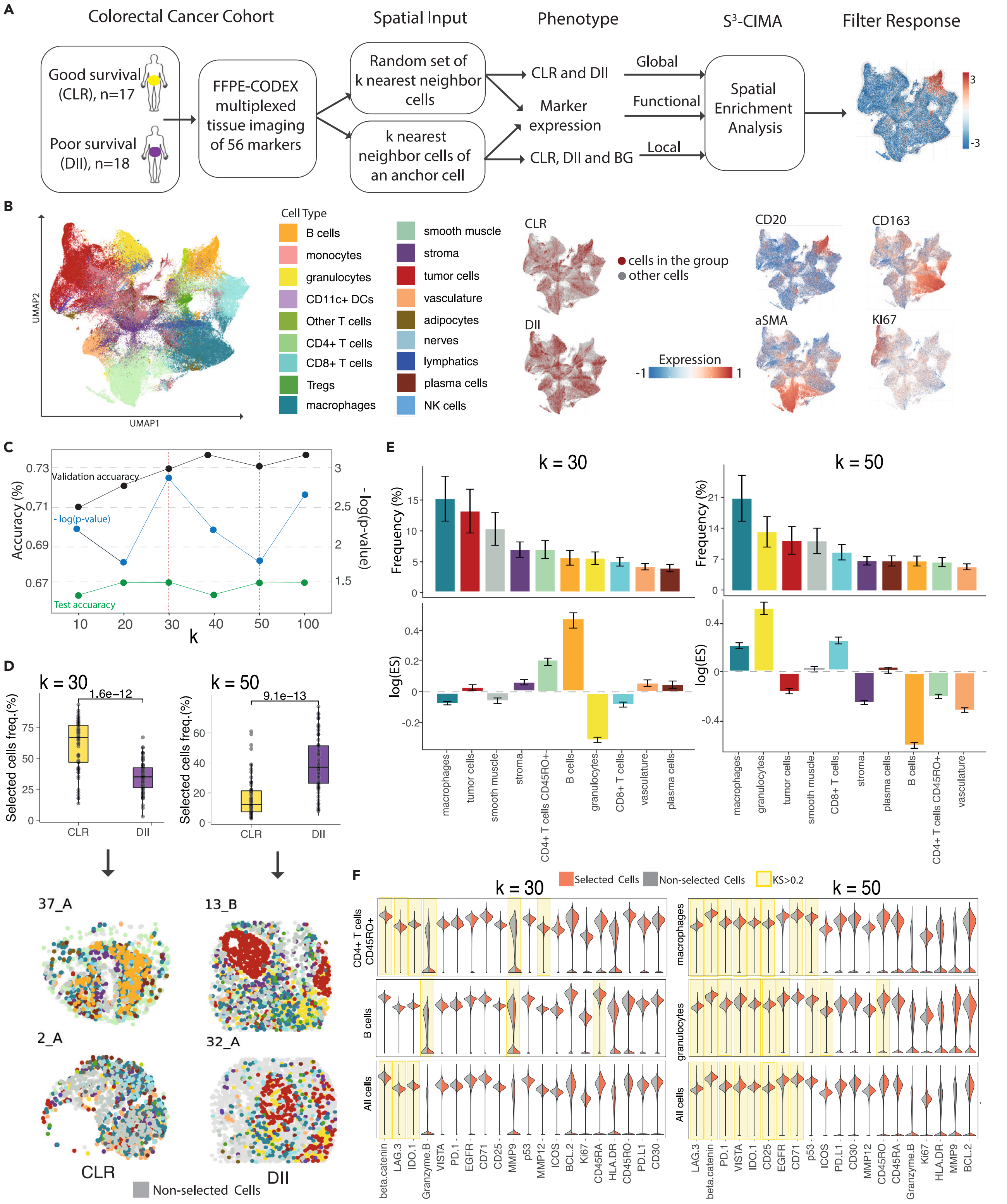

Figure 2. S3-CIMA global spatial enrichment analysis

(A) S3-CIMA global spatial enrichment analysis applied on the 140 CODEX images of 35 CRC patients.

(B) Uniform manifold approximation and projection (UMAP) indicating different cell types in the CRC tumor microenvironment. UMAP plots showing origin of single cells by clinical group of patients (CLR, DII) and expression of indicated markers are shown.

(legend continued on next page)

a cytotoxic T cell) associated with a phenotype indicating the originating condition (e.g., membership to a sample associated with a specific clinical outcome) (Figure 1B). The association between these cell neighborhoods and their phenotype might be conferred by the occurrence of a cell subset not matching to a canonical cell type or, more generally, being unknown *a priori*. S^3-CIMA addresses this challenge by a weakly supervised learning model, i.e., a CNN model[23] that takes these sets of cell profiles as input and learns their association with the phenotype label (Figure 1C; see experimental procedures).

Briefly, the inputs to the S3-CIMA model are multi-cell inputs, i.e., groups of cell profile items, each of which are vectors. Since these cell groups correspond to the cells present in the considered k-NN segments, they do not have a predefined order. In particular, it is expected that only a subset of cells in such k-NN segments are relevant for classification. These relevant cells and their profiles are not known *a priori* and are to be discovered by application of our model. These multi-cell inputs are therefore not applicable as input for regular neural networks, since these expect a vector input, with all entries coming in according to a predefined order. S^3-CIMA addresses this issue by implementing the solution presented in the CellCNN model.[23] As CellCNN, the S^3-CIMA model comprises a convolutional layer composed of filters, a pooling layer, and a classification/regression output. It implements a simple CNN model that is invariant to the permutations of the multicell inputs, and the convolutional units are single-cell abundance profiles, i.e., permutation invariance is achieved via the pooling operation (e.g., max or mean pooling), which is performed across all cells in a multi-cell input.

The filters encode cell composition patterns from the molecular profiles (i.e., protein marker expression levels) by fitting the model to the training data. The pooling layer summarizes these pattern frequencies encoded by each filter, which in turn are used to associate with the classification/regression label (Figure 1D).

The trained model is then used to identify the cell subsets critical for association with the phenotype label (e.g., patient outcome, treatment response, survival). Therefore, the trained filter weights correspond to molecular profiles and not to predefined annotated cell types of cell subsets in the local anchor cell neighborhood. These cell subsets are further characterized with respect to their cell type and molecular profile (Figure 1E). The proximal enrichment of these cell subsets further motivates hypotheses about putative mechanisms conferring this enrichment (e.g., paracrine signaling).

We evaluated the S^3-CIMA workflow for studies based on two different *in situ* proteomic imaging technologies, i.e., CODEX highly multiplexed fluorescence microscopy data of 56 markers in tumor tissues of 35 patients with low- and high-risk CRC, as well as IMC data of 35 markers in pancreas tissues from 12 donors with T1D.

S^3-CIMA identifies spatially enriched cell subsets associated with differential the colorectal cancer outcome

The spatial organization of the TME in CRC, indicating the colocalization and density of immune cells, is linked to disease progression and patient survival.[24,25] Therefore, we applied S^3-CIMA to the data of the CRC study,[20] where patients were stratified in two groups with differential survival, i.e., 17 patients with Crohn's-like reaction (CLR) and 18 patients with diffuse inflammatory infiltration (DII) (Figure 2A). It has been previously reported that overall survival of patients with CLR is significantly longer than that of patients with DII.[26] All cells in the CODEX images had been annotated to 28 unique cell types, including 18 immune, 6 stromal, 2 mixed, and 1 tumor cell groups[20] (Figure 2B, S1). The study showed the differences in the frequency of immune cell compositions between CLR and DII patients in the individual TMEs. Most notably, CLR patients had higher frequencies of B cells and lower frequencies of macrophages than DII patients, respectively (Figure S2). The study determined different cellular neighborhoods, i.e., tissue regions with a spatial localization of a cell type by means of an unsupervised framework, and showed that in the DII patients, PD-1+ $CD4^+$ T cells were enriched within a granulocyte cellular neighborhood.

We expanded this analysis by assessing in an unbiased data-driven fashion the spatial enrichment of specific, possibly non-canonical cell subsets in the CRC TME between CLR and DII patients using the S^3-CIMA model (i.e., global spatial enrichment analysis) (Figure 2A). We selected 100 random subsets of cells that are in k-NNs ($10 \leq k \leq 100$) in each tissue image as the input for S^3-CIMA. Each input was labeled according to the image group as CLR or DII (Figure S5).

The classification model was trained and validated on 12 samples from each CRC group i.e., 24 samples in total randomly split into training and validation sets (80% and 20% respectively) and tested on the remaining 11 samples. The trained model with highest predictive accuracy on the validation set was selected and was used to identify the selected cells, i.e., the cell subset whose molecular profile (and not their annotated cell types;

(C) S^3-CIMA classification model performance (accuracy on test and validation data) and $-$log(p value) of comparing the frequency of selected cells (Wilcoxon test) of the validation set by model across different cell neighborhood sizes k (10–100). The spatial inputs of 24 patients (12 patients from each survival group) were randomly split into training and validation sets (80% and 20%, respectively). The spatial input of the remaining 11 patients was used as the test set. The best neighborhood size was chosen at k = 30 and 50 based on the highest significance of differential frequency of the selected cells in the validation set (Figure S5A). Further, we also observe the high test accuracy for these neighborhood sizes.

(D) The frequency of selected cells (cells in the training validation and test sets with high filter response) between groups in k = 30 (significantly more from CLR group) and k = 50 (significantly more from DII group) are shown. Selected cells (colored by cell type, color legend as in B) are mapped back to the corresponding patient CODEX images in both CLR and DII groups (see also Figure S8, S9, S10, and S11).

(E) The frequency and ES (median value across all patients) of selected cells across the cell types. Bars are colored by cell type. A positive log value of ES indicates the enrichment of the corresponding cell type. The median absolute deviation (MAD) is shown by the error bar.

(F) Density of the marker expression showing greatest differential abundance in terms of the Kolmogorov-Smirnov (KS) two-sample test statistics between the selected and non-selected cell subsets in all cell populations (regardless of the cell types) and between the selected and non-selected specific cell type subsets (see also Figures S6 and S7). The high differential abundance (KS statistics) is highlighted by yellow color. The expression values were normalized between 0 and 1.

Figure 1A) is relevant for classification of the CLR/DII patients (see S^3-CIMA model training).

The best neighborhood size (k) was selected on the basis of the lowest p value of comparing the frequency of selected cells of the validation set (see characterization of spatially enriched cell subsets). To assess test performance, we also evaluated classification performance on the unseen test set (Figures 2C and S5A). To assess whether the selected cells constitute a distinct cell subset, we also evaluated significance of differential frequency of the selected cells for the test set (Figure S5A). The model robustly selected cells that are more frequent in the CLR group for neighborhood size k = 30 ($p = 1.6e{-}12$, Wilcoxon test) and in the DII group for neighborhood size k = 50 ($p = 9.1e{-}13$, Wilcoxon test) (Figures 2D and S5A).

Since the selected cell subset consists of multiple cell types, we assessed the enrichment of each cell type of the selected cells by calculating an enrichment score (ES) across the patient groups, i.e., DII or CLR (see experimental procedures). We found that at k = 30, the selected cells were predominantly from the CLR group and composed of macrophages (16%), tumor cells (14%), smooth muscle (10%), stroma (7%), and $CD4^+$ T cells CD45RO+ (7%) (Figure 2E). The selected $CD4^+$ T cells $CD45RO^+$ and B cells were significantly enriched (i.e., ES > 1) in the CLR group (Figure 2E). For example, the selected B cells and $CD4^+$ T cells $CD45RO^+$ comprise a subset that exhibits significantly lower expression of granzyme B compared to the non-selected cells (Figures 2F and S6). We identified that the frequencies of subsets (i.e., selected cells by S^3-CIMA) of B cells, $CD8^+$ T cells, plasma cells, stroma, and vasculature are significantly higher in the CLR than in the DII group (Figure S3), while the aforementioned cell-type frequencies are not significantly different when all cells are considered (i.e., by unsupervised analysis) (Figure S2). On the other hand, we found that at k = 50, the selected cells were predominantly from the DII group and composed of macrophages (21%), granulocytes (13%), tumor cells (12%), smooth muscle (12%), and $CD8^+$ T cells (8%) (Figure 2E). The selected immune cells have higher expression of the checkpoint molecules LAG3, PD-1, and V-domain immunoglobulin suppressor of T cell activation (VISTA) compared to the bulk of their corresponding cell types that are in the tissue but not selected (Figures 2F and S7). The S^3-CIMA model can thus identify specific cell subsets at different neighborhood sizes that associate with either poor or superior surviving patients (i.e., at k = 30 and k = 50). This suggests that S^3-CIMA captures the disease-associated TME characteristics at multiscale cellular neighborhoods. We also identified that the frequencies of subsets of tumor cells, $CD4^+$ T cells, CD45RO+, stroma, and $CD68^+$ macrophages, which are selected by S^3-CIMA compared with non-selected cells from the same cell types, are significantly higher in the DII than in the CLR group (Figure S4). However, the frequencies of these cell types are not significantly different when all cells are considered (i.e., by unsupervised analysis) (Figure S2). The baseline model relies on differential frequency analysis of *a priori* defined cell types. This model is comparable to a S^3-CIMA model with *a priori* defined filter weights followed by assessing differential frequencies. Furthermore, we evaluated a S^3-CIMA model variant as a two-layer CNN and observed evidence of overfitting, indicated by decrease in classification accuracy on the test set (data not shown).

S^3-CIMA reveals high enrichment of PD-1+ $CD4^+$ T cell subset in granulocyte neighborhoods of DII patients

Schürch et al.[20] defined nine cellular neighborhood classes as tissue regions with a high density of a specific cell type and assessed if the frequencies of these exhibit any differences between two CRC patient groups. This analysis showed that except for the follicle cellular neighborhood, which was highly enriched in CLR patients, none of the other cellular neighborhood categories differed significantly. It also identified that $PD\text{-}1^+$ $CD4^+$ T cells enrichment in the granulocyte neighborhood correlated with overall survival in the DII patient group. The study therefore suggested that to understand the underlying process shaping the CRC TME, cell types and the cellular neighborhood should be considered simultaneously.

S^3-CIMA allows to achieve this goal in an unbiased data-driven fashion by learning disease-associated cellular neighborhoods around specific anchor cell types, i.e., the anchor cell niche (Figure 1B). We considered each cell type in the dataset as the anchor cell type. We generated condition-specific (i.e., CLR or DII) CNN models with the adequate classification accuracy when using B cells, $CD4^+$ T cells, tumor cells, M2 macrophages, and granulocytes as anchor cell types across a range of k-NN sizes ($10 \leq k \leq 50$) (Figure S12). We used the identical training-, validation-, and test-set splits of the global spatial enrichment analysis of S3-CIMA to train and test the models.

Considering granulocytes as the anchor cell type, we found that the frequency of selected cells is significantly higher in the DII group compared to CLR and background at k = 30 ($p = 4.6e{-}5$ and $p = 1.2e{-}15$, Wilcoxon test, respectively) (Figures 3A and S13) and composed of granulocytes (58%), macrophages (7%), tumor cells (6%), and smooth muscle (5%) (Figure 3B).

To assess whether the selected cell types are spatially enriched in the granulocyte neighborhood and are not selected because of the abundance, we calculated an analytical ES for each cell type. The total number of cells of a given annotated cell type in the granulocyte neighborhood does not correlate with a higher ES (i.e., ES > 1). The granulocyte niche is enriched for $CD4^+$ T cells CD45RO+ (ES = 1.14), vasculature (ES = 1.05), and $CD8^+$ T cells (ES = 1.04) (Figure 3B). Interestingly, we confirmed that the granulocyte neighborhood is highly enriched for $CD4^+$ T cells CD45RO+ in the DII group compared to the CLR group (Figure 3C). To characterize the selected cell subset compared to non-selected cells and assess its differential functional capacity, we performed differential marker expression analysis. Specifically, we compared the functional marker expression, such as apoptosis, activation/proliferation, inhibition, and cytokine signaling markers, in the selected subsets of cells versus non-selected cells within each specific cell-type compartment. The selected cells in the granulocytes niche highly express VISTA (KS = 0.48), granzyme B (KS = 0.32), and MMP9 (KS = 0.31) (Figures 3E and S16). We also identified that the spatially enriched $CD4^+$ T cells $CD45RO^+$ subset in the granulocyte niche over-expresses T cell exhaustion/activation markers VISTA (KS = 0.44), PD-1 (KS = 0.22), and epidermal growth factor receptor (EGFR) (KS = 0.28) (Figure 3E). To confirm the protein marker activity of enriched cell subset in the granulocytes niche, we then mapped the selected cells in the granulocyte niche to the CODEX images and observed a high expression of PD-1 and CD4 markers in selected cell subsets (Figures 3D, 3F, S14, and S15).

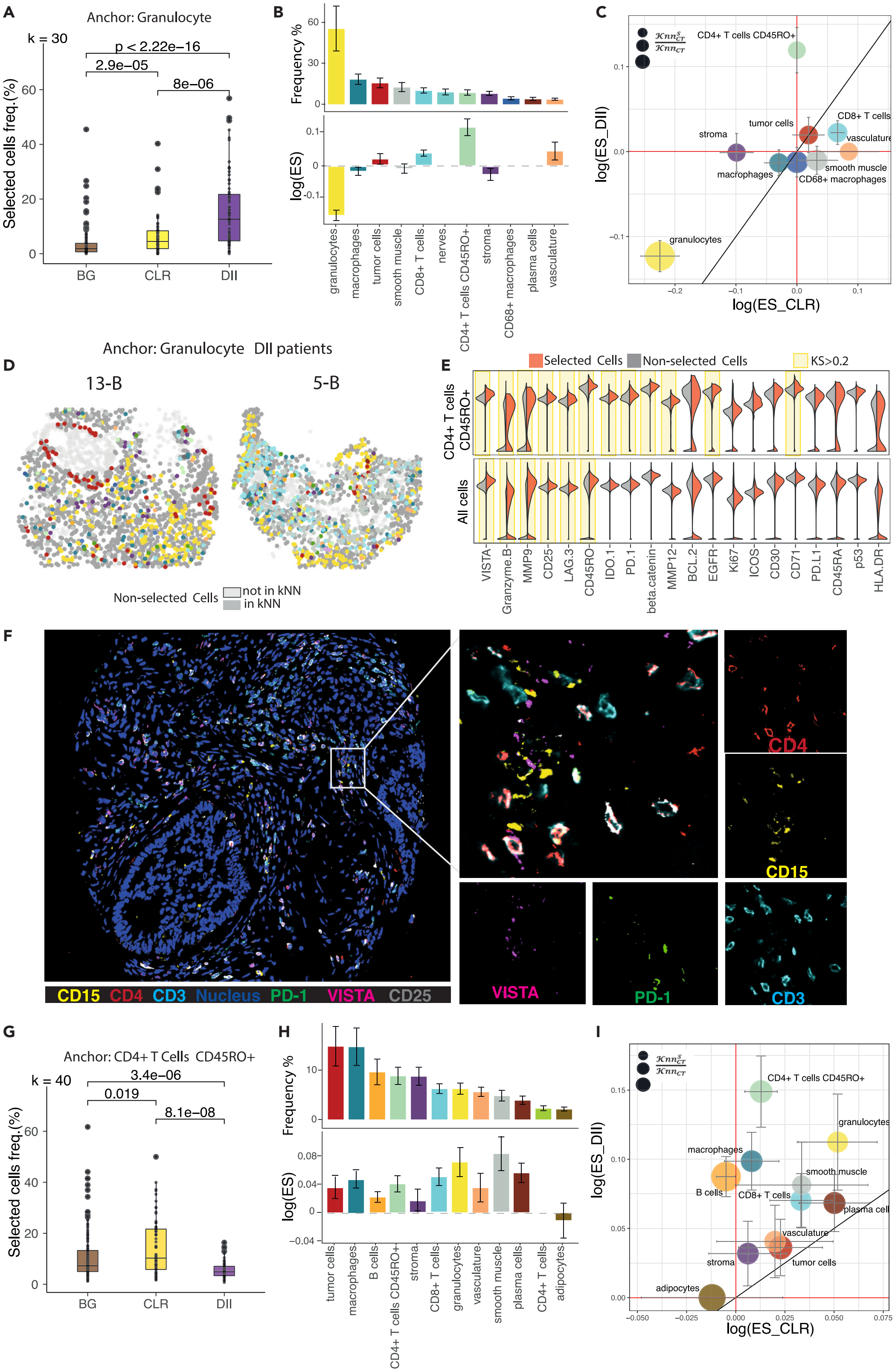

Figure 3. S[3]-CIMA local spatial enrichment analysis

The multi-cell inputs generated from k nearest neighbor cells around granulocytes (k = 30) and CD4^{+} T cells CD45RO+ (k = 40) across all 35 CODEX images of the CRC dataset. Each multi-cell input was labeled by a CRC outcome class (CLR or DII) and background (BG) for the classification task.

(legend continued on next page)

Then, to investigate the mutual interaction of CD4$^+$ T cells CD45RO+ and granulocytes, we performed S^3-CIMA local enrichment analysis considering CD4$^+$ T cells CD45RO$^+$ as the anchor cell type. We found that the frequency of selected cells is significantly higher in the CLR group compared to DII and background at k = 40 (p = 8.1e−8 and p = 0.019, Wilcoxon test, respectively) (Figures 3G and S13) and composed of mostly tumor cells (15%), CD163+ macrophages (15%), and B cells (8%) (Figures 3H, S17, S18, and S19). The enrichment analysis showed that the CD4$^+$ T cells CD45RO + niche is enriched by smooth muscle (ES = 1.08), granulocytes (ES = 1.07), plasma cells (ES = 1.06), and CD8$^+$ T cells (ES = 1.05) (Figure 3I).

We then calculated the ES for each CRC group separately, and interestingly, we also identified the spatially enriched granulocyte subset in the CD4$^+$ T cells CD45RO$^+$ niche, which is enriched in the DII group, indicating a specific mutual interaction of these two cell types (Figure 3I). This interaction can be confirmed by colocalization of representative cells in the original CODEX images (Figure 3F). These results suggest that S^3-CIMA can determine the local enrichment of the subset of a conventional cell type in the spatial neighborhood of a specific cell type as well as the one-to-one cell type interaction.

Functional spatial enrichment analysis reveals an EGFR-expressing cell subset in the proximity of granulocytes

We then performed functional spatial enrichment analysis by S^3-CIMA to identify cell subsets enriched in the proximity of an anchor cell, i.e., granulocytes (the same anchor that we used for the local enrichment analysis) varying in local EGFR expression. EGFR plays a key role in different cellular functions, such as proliferation, apoptosis, and differentiation. It has been shown that high expression of EGFR is common in many tumors. In particular, in CRC, high expression of EGFR is associated with a poor prognosis.[27]

To determine the effect of localized EGFR expression, we considered the phenotype label of a multi-cell input (i.e., cells in the k nearest neighborhood) as the average expression of EGFR over all k cells in the nearest neighborhood of each granulocyte and then trained a regression S^3-CIMA model (R2-score = 63.8%, RMSE = 0.28). To summarize the distribution of selected cells with respect to local EGFR expression, we report selected cell frequencies for either high or low, i.e., higher or lower than average EGFR expression across all considered cell neighborhoods. The model learns the spatial characteristics of both groups with two discriminative filters. We found that the frequency of selected cells is significantly higher in the EGFR^{Low} group compared to EGFR^{High} for filter 1 and vice versa for filter 2 at k = 20 (p = 2.2e−16, Wilcoxon test) (Figure 4A). Considering the response of filter 1, we identified that the granulocyte niche is highly enriched in adipocytes, plasma cells, and CD8$^+$ T cells in the EGFR^{Low} group (Figures 4B and 4C). The selected cell subset in the granulocyte niche is associated with significantly reduced EGFR, CD25, and Ki67 expression (Figure 4D). The response of filter 2 determined that the granulocyte niche is highly enriched by B cells and tumor cells in the EGFR^{High} compared to the EGFR^{Low} group (Figure 4B). The spatially enriched B cells subset in the granulocyte niche significantly over-expresses the interleukin-2 receptor CD25 (KS = 0.35), EGFR (KS = 0.34), and PD-L1 (KS = 0.32) (Figure 4D). This result shows that colocalization of granulocytes and B cells is associated with high expression of EGFR in the TME. With this case, we demonstrated that S^3-CIMA is capable of identifying determinants of niche composition that are associated with local signaling activity in the tissue such as EGFR signaling (Figure 4E).

S^3-CIMA reveals high enrichment of beta cells in cytotoxic T cell neighborhoods in T1D onset patients

We examined if S^3-CIMA is also capable of finding a spatial pattern of cells in imaging mass cytometry (IMC) data. To accomplish this, we used a T1D study including IMC measurement of 35 protein markers of pancreas tissues from 12 donors, comprising healthy, T1D disease onset, and long disease duration conditions[22] (Figure 5A). T1D is an autoimmune disease that is caused by an immune system attack on insulin-producing

(A) Frequency of selected cells across the patients between groups with granulocytes as an anchor. The cells with the high filter response have a significantly increased frequency in the DII group (Wilcoxon test).

(B) Frequency and ES (median value across all patients) of selected cells across the cell types obtained from local enrichment spatial analysis for granulocytes as the anchor. The median absolute deviation (MAD) is shown by the error bar. Bars are colored by cell type. A positive log value of the ES indicates the enrichment of the corresponding cell type.

(C) Bubble plot of ES of each cell type in the proximity of granulocytes comparing different survival groups (median of ESs across all patients). The MAD is shown by the error bar. The color and size of the bubbles indicate the cell types (color legend as in Figure 2B) and the ratio of the number of selected cells to the total number of cells of the specific type, respectively.

(D) Selected cells (colored by cell type; color legend as in Figure 2B) are mapped back to the corresponding patient CODEX images in the DII groups (Figures S14 and S15).

(E) Density of functional marker expression showing greatest differential abundance in terms of the KS two-sample test statistics between the selected and non-selected cell subsets in all cell populations (regardless of the cell types) and between the selected and non-selected specific cell type subsets (see also Figure S16). The expression values were normalized between 0 and 1.

(F) Codex image of patient 30 (DII group). The spatial neighborhood of one granulocyte as the anchor is zoomed in. The PD-1, CD4, CD15, and CD68 markers intensities of the selected cells around this anchor are shown.

(G) Frequency of selected cells across the patients between groups with CD4$^+$ T cells CD45RO + as an anchor. The cells with the high filter response have significantly increased frequency in the CLR group (Wilcoxon test).

(H) Frequency and ES (median value across all patients) of selected cells across the cell types obtained from local enrichment spatial analysis for CD4$^+$ T cells CD45RO$^+$ as the anchor. The MAD is shown by the error bar. Bars are colored by cell type. A positive log value of the ES indicates the enrichment of the corresponding cell type.

(I) Bubble plot of ES of each cell type in the proximity of CD4$^+$ T cells CD45RO$^+$ comparing different survival groups (median of ESs across all patients). The MAD is shown by the error bar. The color and size of the bubbles indicate the cell types (color legend as in Figure 2B) and the ratio of the number of selected cells to the total number of cells of the specific type, respectively.

CellPress
OPEN ACCESS

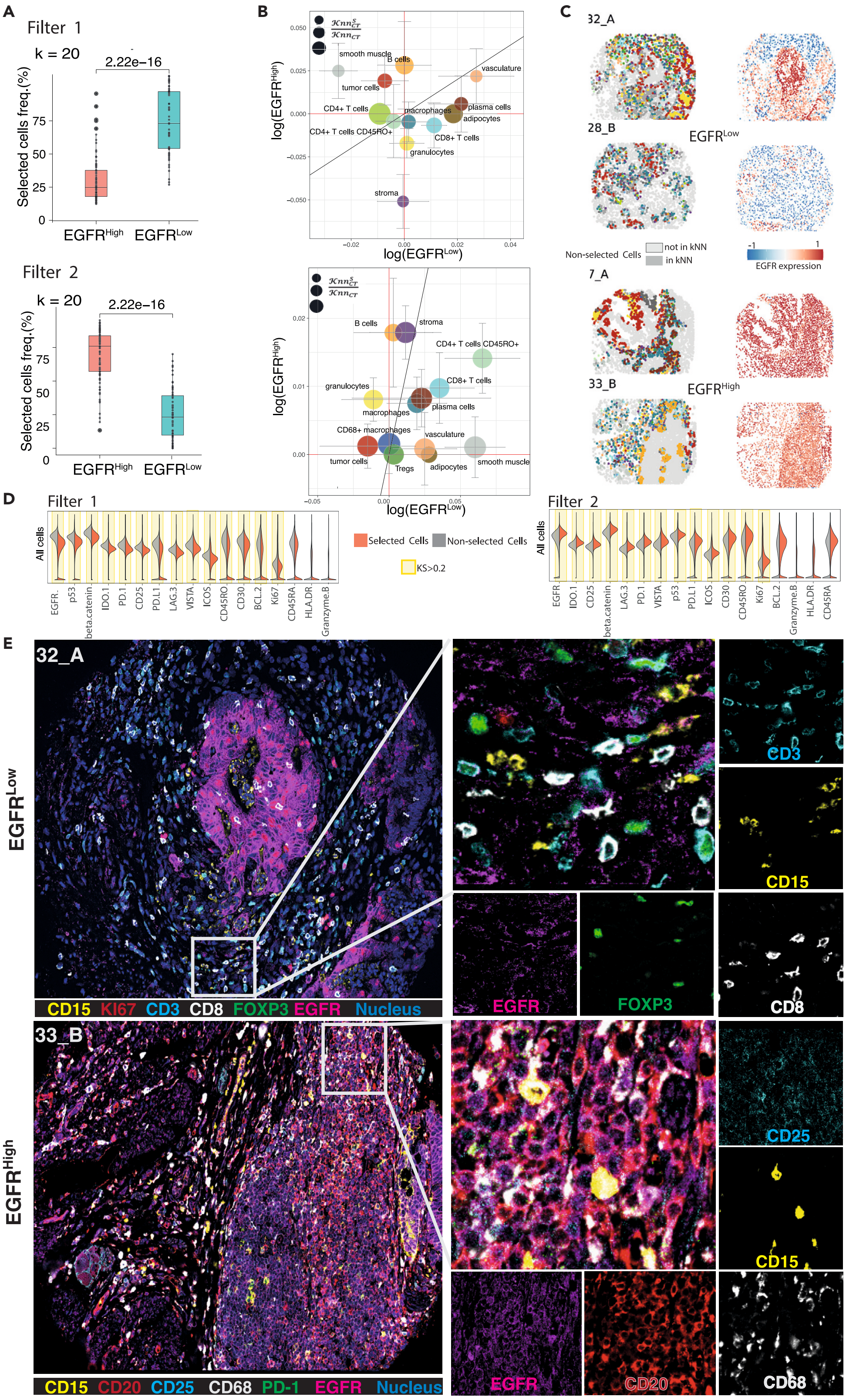

Figure 4. S^3-CIMA functional spatial enrichment analysis

(A) The multi-cell inputs generated from k nearest neighbor cells around granulocytes (k = 20) across all 35 CODEX images of the CRC dataset. Each multi-cell input was labeled by the average of EGFR marker expression of the cells in that multi-cell input (continuous value) as a surrogate for local signal transduction

(legend continued on next page)

beta cells in the pancreatic islets of Langerhans. The spatial interaction between islets and immune cells can be involved in the progression of the disease.[28] This study demonstrated that beta cell destruction is preceded by recruitment of cytotoxic and helper T cells in T1D disease onset by applying histoCAT, which is an unsupervised neighborhood analysis.[16]

We performed S^3-CIMA analysis for local spatial enrichment analysis to understand association between islet cells and immune cells during T1D progression. The study showed that the association between immune cells and islet cells is not common; however, during T1D onset, because of the assembly of a destructive immune reaction, cytotoxic and helper T cells are recruited to beta cell-rich islets.[29] Similarly, we also observed that the presence of immune cells (monocytes, neutrophils, and B cells) in spatial proximity of islet cells (alpha, beta, gamma, and delta cells) is not frequent in all three disease stages, i.e., there is no spatially enriched subset of islet cells when any of these immune cell types is an anchor cell. However, we observed that when we performed S^3-CIMA with T cells as an anchor (T helper cells, cytotoxic T cells) (Figure 5B) and naive T cells (Figure S24), there is a high enrichment of the presence of beta cells in the neighborhood of cytotoxic T cells from the T1D onset group (Figures 5C and 5D). It is known that cytotoxic and helper T cells are involved in the destruction of beta cells in T1D.[30] This result confirms the previous finding[22] that showed that beta cell destruction is preceded by recruitment of cytotoxic and helper T cells in T1D disease onset. In summary, we find that S^3-CIMA is capable of discovering novel disease-associated spatial cellular interactions from various spatial biology data types.

DISCUSSION

Understanding how cell type compositions in the TME differ from one disease condition to another can pinpoint tentative disease mechanisms. S^3-CIMA provides a supervised spatial enrichment analysis by learning from disease-associated TME composition to systematically identify spatially enriched cell subsets associated with the disease conditions. We showed that S^3-CIMA achieves this goal in an unbiased data-driven fashion by learning disease associated cellular neighborhoods around specific anchor cell types (local spatial enrichment analysis) or random sets (global spatial enrichment analysis). We demonstrated, for both CODEX and IMC data, that S^3-CIMA can reveal novel subpopulation structures and tissue organization possibly missed by single modalities or other methods. The frequencies of selected subpopulations are significantly different across the phenotypes (e.g., CLR and DII groups in the CRC cohort analyses), while the frequency of the whole population of those cell types (i.e., unsupervised analysis) are similar in the phenotype groups (Figures 2, 3, and 4). Identified cellular subpopulations can, in principle, always be further subdivided to reflect a finer characterization of observed heterogeneity. Subdivision based on multiple modalities provides an opportunity to identify more meaningful biological distinctions.

Here, we examined the S^3-CIMA model on antibody-based proteomic imaging data that is inherently limited to subgenome-wide analyses. This limited spectrum of molecular parameters possibly precludes the detection of sample condition-associated cell subsets defined by unmeasured molecular markers. However, the increase in spatial resolution of spatial transcriptomic approaches is likely going to alleviate this limitation and allow S^3-CIMA to comprehensively assess the cell states across genome-wide expression profiles. Further, the greedy nature of the model fitting can fail to report the cell subsets that are less but still significantly correlated with the supervision signal than the selected cell subset.

How supervised are the baseline methods of this study that are termed unsupervised? The use of predefined cell types can be considered as introducing supervision by focusing on *a priori* promising cell subsets and excluding a prior unlikely relevant one from the analysis. Considering predefined cell types injects prior knowledge into TME model learning. It can be compared to feature engineering in, e.g., computer vision applications, where for image classification specific image features (e.g., edges) are extracted and used as inputs to both unsupervised and supervised learning tasks (e.g., clustering or respectively classification).

S^3-CIMA operates by evaluating k nearest neighborhoods and selects their size in a data driven fashion (experimental procedures). The evaluation of the best-performing k settings allows for speculation of the type of interaction mechanism conferring the association of spatial cell type enrichment and phenotype. While small k settings (e.g., $k = 10, 20$) suggest direct cell-cell interactions, e.g., through direct binding between surface receptors and ligands, larger k settings (e.g., $k = 30, 40, 50$) rather suggest indirect interaction through intercellular signaling molecules such as secreted cytokines.

Up to now, most of the spatial proteomic imaging data analysis efforts applied unsupervised methods that cannot use the associated external cues of interest directly and therefore can only report descriptions of—possibly enriched—cell-cell interactions (Figure S26). However, an increasing number of spatial proteomic imaging studies in the field of health and disease are and will be comparative, i.e., aiming at identifying spatial cell-type

activity for the regression task. Frequency of selected cells of functional enrichment analysis with granulocytes as an anchor comparing two groups of patients with high and low EGFR marker expression. The cells with a high filter response have a significantly increased frequency in the $EGFR^{Low}$ group in filter 1 and $EGFR^{High}$ group in filter 2 (Wilcoxon test).

(B) Bubble plot of ES (median of ESs across all patients).of each cell type in the proximity of granulocytes showing the cytokine signaling activity of the EGFR marker in each group. The MAD is shown by the error bar. The color and size of the bubbles indicate the cell types (color legend as in Figure 2B) and the ratio of the number of selected cells to the total number of cells of the specific type, respectively.

(C) Selected cells (colored by cell type; color legend as in Figure 2B) and the EGFR expression value for each cell are mapped back to the corresponding patient CODEX images in both $EGFR^{High}$ and $EGFR^{Low}$ groups (also Figures S20, S21, S22, and S23).

(D) Density of functional marker expression showing greatest differential abundance in terms of KS two-sample test between the selected and non-selected cell subsets in all cell populations and per cell types for regression task. The expression values were normalized between 0 and 1.

(E) Codex images of patient 16 (Spot 32_A, DII group, $EGFR^{Low}$) and patient 17 (Spot 33_B, CLR group, $EGFR^{High}$). The spatial neighborhood of one granulocyte as the anchor is zoomed in. The mentioned marker intensities of the selected cells around this anchor are shown.

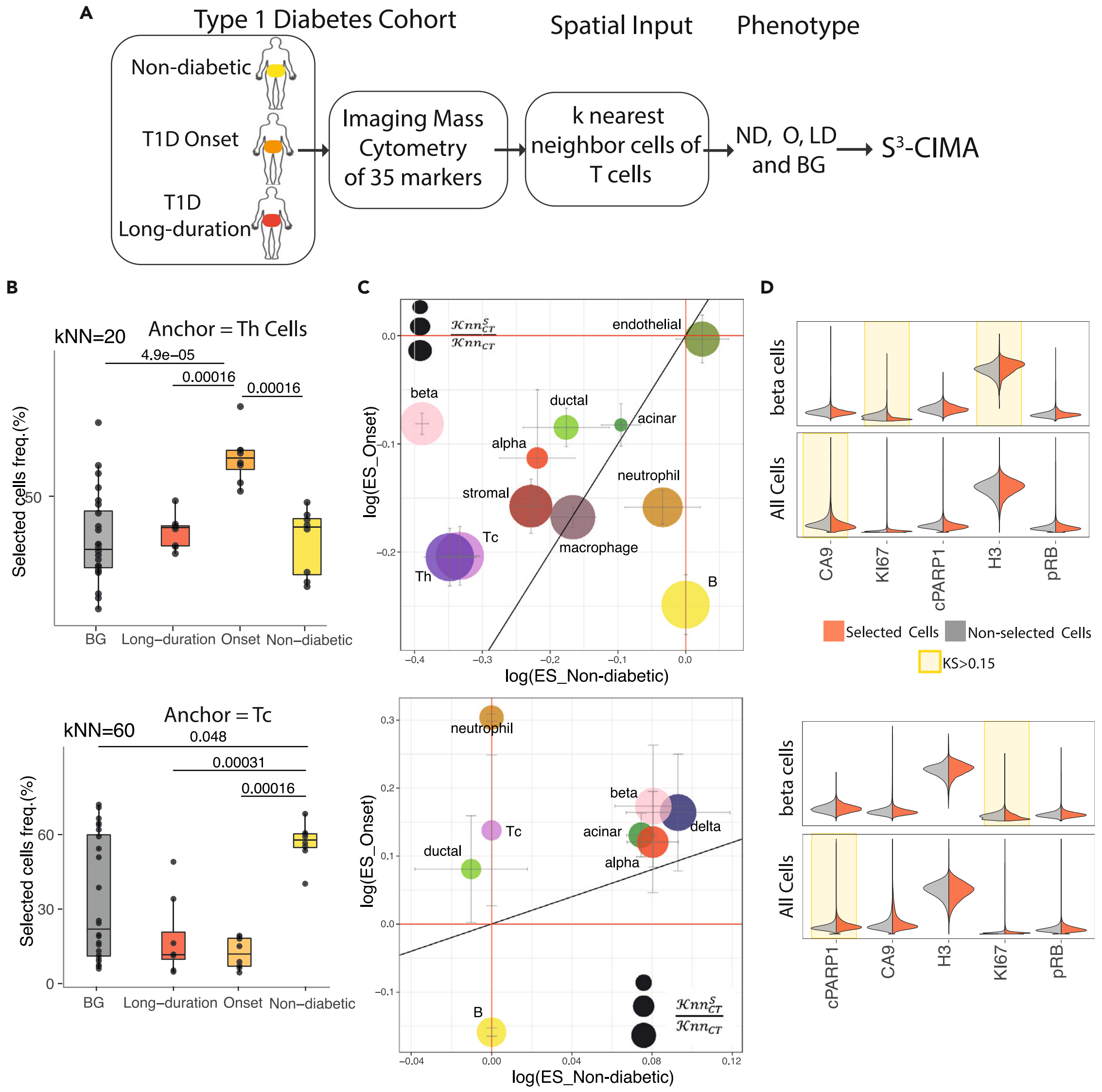

Figure 5. S3-CIMA local spatial enrichment analysis

(A) S3-CIMA local spatial enrichment analysis was applied on IMC measurement of 35 protein markers of pancreas tissues from 12 human donors, comprising healthy, T1D onset, and long disease duration conditions. The spatial inputs of 9 patients (3 patients from each disease condition group) were randomly split into training and validation sets (80% and 20% respectively). The spatial input of the remaining 3 patients were used as the test set.

(B) Frequency of selected cells between disease stages with helper T cells as an anchor. The cells with the high filter response have significantly increased frequency in the onset T1D group (Wilcoxon test). Frequency of selected cells between disease stages with cytotoxic T cells as an anchor. The cells with the high filter response have significantly increased frequency in the non-diabetic group (Wilcoxon test).

(C) Bubble plot of ES (median of ESs across all patients) of each cell type in the proximity of helper T cells shows that there is no high enrichment of the presence of any cell type. The color and size of the bubbles indicate the cell types and the ratio of the number of selected cells to the total number of cells of the specific type, respectively. Bubble plot of ES (median of ESs across all patients) of each cell type in the proximity of cytotoxic T cells shows that there is a high enrichment of the presence of beta cells from the onset T1D group. The MAD is shown by the error bar.

(D) Density of functional marker expression showing greatest differential abundance in terms of KS two-sample test between the selected and non-selected cell subsets in all cell populations and beta cells. The expression values were normalized between 0 and 1.

composition patterns that explain external cues, such as physiological state, disease state, therapy response or signal transduction activity. The S3-CIMA model is designed for the analyze of individual studies, and therefore, if a dataset is provided from a new study that has been acquired under different study conditions (e.g., other marker panel), a new S3-CIMA model fit/selection is necessary.

We showed that S3-CIMA can use both discrete and continuous states of these external cues as the sample phenotypes. Other supervised models such as graph neural networks (GNNs) are in principle suited alternatives for this task. While the application of standard GNNs achieved inferior classification accuracy (Figure S27), we expect that further dedicated method development might yield at least a complementary approach to

supervised analysis of multiplexed imaging data. In summary, S3-CIMA constitutes a machine learning model that addresses this issue and—as first of its kind—is expected to enable productive interpretation of such comparative studies in the future, as for instance the recent study of the cellular and functional composition of the mucosal-associated invariant T cells (MAIT) cells niche in hepatocellular carcinoma (HCC) using CODEX images of HCC patient samples, where by virtue of S3-CIMA, we identified CSF1R+ PD-L1+ tumor-associated macrophages as key interacting players in the MAIT cell neighborhood in the adjacent liver, outside the tumor.[31]

EXPERIMENTAL PROCEDURES

Resource availability

Lead contact

Requests for further information and resources should be directed to the lead contact, Manfred Claassen (Manfred.Claassen@med.uni-tuebingen.de).

Materials availability

This study did not use or generate new materials.

Data and code availability

This paper analyzes existing, publicly available data as referenced in below subsection Datasets. The code of the S3-CIMA workflow has been deposited at https://github.com/claassenlab/S3-CIMA. DOI: https://doi.org/10.5281/zenodo.8189619.

Notation used throughout the paper

C: multi-cell input
(X, Y): position of a cell
k: number of nearest neighbor cells
T: cell type
p: p value
k-NN: k-nearest neighbors
KS: Kolmogorov-Smirnov test statistic

Datasets

Colorectal cancer

Here, we used the data of an already published CRC cohort including 56-marker multiplexed CODEX tissue imaging data of 35 CRC patients including 140 tissue microarray (TMA) spots, i.e., 4 CODEX images for each patient.[20] The marker set includes lymphocyte and myeloid cell canonical markers, functional immune cell markers (e.g., cytokine signaling, activation/proliferation, inhibition/checkpoint markers), and several auxiliary markers.

The flat table of the images was used for computing the spatial input of S3-CIMA indicating 258,385 cells across 140 CODEX images of 35 patients as rows and patient ID, marker intensity, position (i.e., X/Y coordinates), survival group (i.e., DII or CLR), and annotated cell type (29 cell types) as columns. Marker intensity values were log transformed by adding an offset (log(1e−3+ x)) and scaled by *Z* score across the dataset prior to calculating spatial inputs (We followed the same preprocessing procedure of Schürch et al.[20]). The spatial inputs of 24 patients (12 patients from each survival group) were randomly split into training and validation sets (80% and 20% respectively). The spatial input of the remaining 11 patients were used as the test. We calculated the nearest neighbors and enrichment analysis per TMA spots (i.e., images).

Type 1 diabetes

The T1D cohort includes IMC measurement of 35 protein markers of pancreatic islets of Langerhans tissues from 12 human donors, comprising healthy (n = 4), recent-onset T1D (<0.5 years, n = 4), and long-standing T1D duration (>8 years, n = 4).[22] The data includes two sections originating from different anatomical regions of the pancreas (tail, body, or head) for each donor. The flat table of the IMC images was used for computing the spatial input of S3-CIMA indicating 1,776,974 cells across 845 IMC images of 12 patients as rows and patient id, slide ID (24 slides), cell position in each image (i.e., X/Y coordinates), cell category (exocrine, immune, islet, other, unknown), and cell type (16 cell type) as columns. Marker intensity values were transformed and scaled by *Z* score across the dataset prior to calculate spatial inputs. The spatial inputs of 9 patients (3 patients from each disease condition group) were randomly split into training and validation sets (80% and 20% respectively). The spatial input of the remaining 3 patients were used as the test set.

S3-CIMA model training

S3-CIMA implements a weakly supervised CNN model to identify cell subsets whose frequency distinguishes the considered phenotype labels (i.e., disease associated conditions). The model is adopted from the CellCNN model,[23] comprising a single layer CNN and a pooling layer and a classification or regression output and using groups of cell expression profiles (multi-cell inputs) as input. The CNN is invariant to the permutations achieved via the pooling operation (e.g., max or mean pooling), which is performed across all cells in a multi-cell input. S3-CIMA uses multi-cell inputs, which are generated k nearest cell neighbors in a tissue image per class label with a set of marker expressions as the features (global spatial enrichment analysis). For local spatial enrichment analysis, S3-CIMA uses multi-cell inputs as *k* nearest cell neighbors around anchor cells of a specific cell type. Training and testing of the model are performed as described in Arvaniti et al.[23] Briefly, we consider a cross-validation training procedure and split the data into three independent subsets, training-, validation-, and test sets. We used the training set to train the CNN model using different hyperparameters (number of filters, learning rate, dropout) values. We trained 200 models on the training set and the model with highest predictive accuracy on the validation set was selected. The performance of the trained model to predict the unseen samples and to not overfit was assessed by calculating the classification accuracy on the test set.

Global spatial enrichment analysis

Global spatial enrichment analysis aims at identifying cell subsets that are spatially co-localized across the multiple conditions. The multi-cell input is generated using nearest neighbor cells of N randomly selected anchor cells in each tissue image with a set of marker expressions as the features. A multi-cell input is the set of marker expression vectors of the k nearest neighbor cells $[\mathbf{C}_i]_{KM}$, i ∈ {1, 2, …,*N*}, where *K* and *M* are the number of cells and number of markers, respectively.

We performed S3-CIMA global spatial enrichment analysis of the CRC cohort by selecting *N* random cells in each patient CODEX image and calculating *k-NN* ($10 \leq k \leq 100$) based on the Euclidean distance $\mathbf{C}_i$. Each input was labeled according to its corresponding image group as CLR or DII. Since some TMA images include only 200 cells, we selected n = 100 random cell subsets as the spatial multi-cell inputs of the classification model.

To control for variation due to the random selection of training and test sets, we repeated this approach with different seeds and noted that the classification accuracy did not differ significantly. (Figure S5C). Furthermore, we evaluate the robustness of the obtained results in terms of the ES of the selected cells that are significantly higher in one class. Specifically, we showed that in the global enrichment analysis at K = 50 the best model showed the enrichment of the CD8 T cells, granulocytes and macrophages in the DII survival group (ES > 1, Figure 2E). We showed that by repeating the model training with different seeds the ES of the selected cell types did not differ significantly (Figure S5C).

Local spatial enrichment analysis

Local spatial enrichment aims at identifying cell subsets that are enriched in spatial proximity of an anchor cell with a specific cell type (e.g., granulocytes) across multiple conditions. The multi-cell inputs k nearest neighbors of an anchor cell are denoted $[\mathbf{C}_i]_{KM}$, i ∈ {1, 2,…*N*}, where *N*, *K* and *M* are the number of the anchor cell, number of neighboring cells and number of markers, respectively. We added a set of randomly selected k nearest neighbor cells as the background (BG) label to ensure that the cell signatures identified by the S3-CIMA indicate the anchor cell specific spatial colocalization.

Functional spatial enrichment analysis

Functional spatial enrichment analysis aims at identifying cell subsets enriched in the proximity of a specific functional activity of a specific anchor cell type, e.g., local proliferative activity of tumor cells. We examined the S3-CIMA ability to detect spatial enriched functional subset using CRC dataset. The preprocessing and computing of the multi-cell input $[C_i]_{KM}$ is similar to the local

spatial enrichment analysis. However, the multi-cell inputs were labeled according to the specific functional activity i.e., the average expression of the marker of interest over all cells in $[C_i]_{K(M-1)}$. Here, the functional marker of interest was excluded and therefore the M-1 marker intensities of the k-NN of that anchor cell type were the features. The average of the functional marker intensities of the multi-cell inputs were used to capture the functional activity as the dependent variable during training of the model.

Quality of prediction of the regression model

To measure the accuracy and the predictive ability of the training, we used the R^2 score and root mean square error (RMSE), respectively. The R^2 score, shows the amount of variance of the true values, which can be explained by the independent variable, and therefore gives a probability about how well unseen samples can be predicted. It is defined as:

$$R^2(y,\hat{y}) = 1 - \frac{\sum_{i=1}^{n}(y,\hat{y})^2}{\sum_{i=1}^{n}(y,\bar{y})^2}$$

where y is the true value and $\hat{y}$ is the predicted value of the sample from a total of n samples. The term $\bar{y}$ is the average of the true values.

The measure of RMSE, gives an idea of the standard deviation of the predictive error, which is the difference between the observed and predicted values. RMSE captures how spread out these values are and is obtained by taking the square-root of the mean squared error, another common metric for measuring the accuracy of the model.

Characterization of spatially enriched cell subsets

The downstream analysis using the trained filter weights facilitates the model interpretation and identifies subset of cells that are associated to the specific phenotype label. The subset of cells with a high filter response (i.e., selected cells) indicates cells with a distinct signature to manifest the phenotype associated aspects of the TME. We calculated the filter response(s) for all individual cells in the training- validation- and test sets using the trained filter(s) weights. We selected and performed downstream analyses for the cells with the positive filter response in most of the analysis unless stated. The relative frequency of selected cells of all input cells per patient in each tissue type as well as background were calculated and compared using a Wilcoxon rank-sum test. The best neighborhood size (k) of each anchor was selected such that a distinct cell subset with significantly differential frequency across the considered conditions is identified. To this end k is chosen to optimize the p value of comparing the frequency of selected cells in the validation set. For local enrichment analysis to select the best k, we considered two comparisons to be significant to ensure that the cell signatures identified by the S^3-CIMA indicate the anchor cell specific spatial colocalization. First, we (1) only consider those neighborhood sizes in which the frequency of selected cells of the conditions (e.g., CLR or DII) were significantly different and then (2), we rank order the remaining neighborhood sizes according to the significance level of the differences in the frequency of selected cells between the chosen condition and the background set. To assess test performance, we also evaluated classification performance on the unseen test set. Further analyses with the model focus on analyzing selected cells.

We assessed the cell type composition and enrichment for selected cell subsets in local spatial enrichment analysis. Therefore, we defined an ES that quantifies the association or exclusion of selected cells of specific cell type in the spatial proximity of the anchor cell.

We consider two quantities for calculating the ES per cell type, (1) frequency of cell type with high filter response in the spatial neighborhood of the anchor cells and (2) frequency of cell type with high filter response outside of the spatial neighborhood of the anchor cells (Figure S25). We define the score of cell type T to be in the nearest neighborhood of the anchor cell and selected:

$$S_T^S = \frac{K_T^S}{E_T^S}$$

We define the score of cell type T to be in nearest neighborhood of the anchor cell:

$$S_T = \frac{K_T}{E_T}$$

where K_T^S is the number of selected cell type T in the nearest neighborhood of the anchor cell, K_T is the number of cell type T in the nearest neighborhood of the anchor cell, E_T and E_T^S are expected value of cell type T be presented in the nearest neighborhood of the anchor cell and expected value of cell type T be selected in the nearest neighborhood of the anchor cell, respectively and calculated as follow:

$$E_T^S = K^S \times \frac{N_T^S}{N^S}$$

$$E_T = K \times \frac{N_T}{N}$$

Where the variables K being the number of all cells in the nearest neighborhood of the anchor cell, N the number of all cells in the image, N_T the number of all cells of cell type T in the image, K^S the number of all selected cells in the nearest neighborhood of the anchor cell, N^S the number of all selected cells and N_T^S the number of all selected cells of type T.

The ES for cell type T is then defined by:

$$ES_T = \frac{S_T^S}{S_T}$$

The ES value above one indicates enrichment of the selected cells of type T in the spatial neighborhood of the anchor cell.

The ES of the global spatial enrichment analysis is calculated by:

$$E_T^S = N^S \times \frac{N_T}{N}$$

$$ES_T = \frac{N_T^S}{E_T^S}$$

The ES of a specific cell type across the patient cohort is reported as the median value of the scores across all patients. The error bars are calculated as the MAD.

Differentially marker expression analysis

To characterize the spatially enriched cell subset, we examine if the functional marker expression in selected subsets significantly differed from non-selected one. We quantify the difference in marker expression distribution by calculating the Kolmogorov–Smirnov two-sample test statistic (KS score) for each marker per cell type. The KS score equals to 1 indicates the highest effect size between two distributions and the KS score equals to 0 indicates that two distributions are similar.

SUPPLEMENTAL INFORMATION

Supplemental information can be found online at https://doi.org/10.1016/j.patter.2023.100829.

ACKNOWLEDGMENTS

This research was supported by the DFG Excellence Clusters EXC 2180 and EXC 2064 (S.B.) and the Department of Pathology and Neuropathology (C.M.S.).

AUTHOR CONTRIBUTIONS

Conceptualization, M.C.; methodology, S.B., M.C.; software, S.B.; validation, S.B., J.C., M.C., C.M.S., A.M., M.Z; formal analysis, S.B., J. C., M.C.; investigation, S.B., M.C., C.M.S.; resources, S.B; data curation, S.B; writing – original draft, S.B., M.C.; writing – review and editing, M.C., S.B., C.M.S., K.W.H.; visualization, S.B., M.C., C.M.S., A.M., M.Z; supervision, M.C., C.M.S. All authors edited the manuscript and approved its final version.

DECLARATION OF INTERESTS

M.C. is a co-founder of, is a scientific advisor to, and holds stock in Scailyte AG. This work is independent of this status. C.M.S. is a scientific advisor to, has stock options in, and has received research funding from Enable Medicine, Inc., all outside of this work.

Received: January 18, 2023
Revised: June 27, 2023
Accepted: August 1, 2023
Published: August 17, 2023

REFERENCES

1. Gerner, M.Y., Kastenmuller, W., Ifrim, I., Kabat, J., and Germain, R.N. (2012). Histo-cytometry: A method for highly multiplex quantitative tissue imaging analysis applied to dendritic cell subset microanatomy in lymph nodes. Immunity *37*, 364–376. https://doi.org/10.1016/j.immuni.2012.07.011.
2. Qi, H., Kastenmüller, W., and Germain, R.N. (2014). Spatiotemporal basis of innate and adaptive immunity in secondary lymphoid tissue. Annu. Rev. Cell Dev. Biol. *30*, 141–167. https://doi.org/10.1146/annurev-cellbio-100913-013254.
3. Baptista, A.P., Gola, A., Huang, Y., Milanez-Almeida, P., Torabi-Parizi, P., Urban, J.F., Shapiro, V.S., Gerner, M.Y., and Germain, R.N. (2019). The Chemoattractant Receptor Ebi2 Drives Intranodal Naive CD4+ T Cell Peripheralization to Promote Effective Adaptive Immunity. Immunity *50*, 1188–1201.e6. https://doi.org/10.1016/j.immuni.2019.04.001.
4. Thornton, C.A., Mulqueen, R.M., Torkenczy, K.A., Nishida, A., Lowenstein, E.G., Fields, A.J., Steemers, F.J., Zhang, W., McConnell, H.L., Woltjer, R.L., et al. (2021). Spatially mapped single-cell chromatin accessibility. Nat. Commun. *12*, 1274. https://doi.org/10.1038/s41467-021-21515-7.
5. Cardozo Gizzi, A.M. (2021). A Shift in Paradigms: Spatial Genomics Approaches to Reveal Single-Cell Principles of Genome Organization. Front. Genet. *12*, 780822. https://doi.org/10.3389/fgene.2021.780822.
6. Giesen, C., Wang, H.A.O., Schapiro, D., Zivanovic, N., Jacobs, A., Hattendorf, B., Schüffler, P.J., Grolimund, D., Buhmann, J.M., Brandt, S., et al. (2014). Highly multiplexed imaging of tumor tissues with subcellular resolution by mass cytometry. Nat. Methods *11*, 417–422. https://doi.org/10.1038/nmeth.2869.
7. Lin, J.R., Fallahi-Sichani, M., Chen, J.Y., and Sorger, P.K. (2016). Cyclic Immunofluorescence (CycIF), A Highly Multiplexed Method for Single-cell Imaging. Curr. Protoc. Chem. Biol. *8*, 251–264. https://doi.org/10.1002/cpch.14.
8. Goltsev, Y., Samusik, N., Kennedy-Darling, J., Bhate, S., Hale, M., Vazquez, G., Black, S., and Nolan, G.P. (2018). Deep Profiling of Mouse Splenic Architecture with CODEX Multiplexed Imaging. Cell *174*, 968–981.e15. https://doi.org/10.1016/j.cell.2018.07.010.
9. Black, S., Phillips, D., Hickey, J.W., Kennedy-Darling, J., Venkataraaman, V.G., Samusik, N., Goltsev, Y., Schürch, C.M., and Nolan, G.P. (2021). CODEX multiplexed tissue imaging with DNA-conjugated antibodies. Nat. Protoc. *16*, 3802–3835. https://doi.org/10.1038/s41596-021-00556-8.
10. Vickovic, S., Eraslan, G., Salmén, F., Klughammer, J., Stenbeck, L., Schapiro, D., Äijö, T., Bonneau, R., Bergenstråhle, L., Navarro, J.F., et al. (2019). High-definition spatial transcriptomics for in situ tissue profiling. Nat. Methods *16*, 987–990. https://doi.org/10.1038/s41592-019-0548-y.
11. Salmén, F., Ståhl, P.L., Mollbrink, A., Navarro, J.F., Vickovic, S., Frisén, J., and Lundeberg, J. (2018). Barcoded solid-phase RNA capture for Spatial Transcriptomics profiling in mammalian tissue sections. Nat. Protoc. *13*, 2501–2534. https://doi.org/10.1038/s41596-018-0045-2.
12. Liu, Y., Yang, M., Deng, Y., Su, G., Enninful, A., Guo, C.C., Tebaldi, T., Zhang, D., Kim, D., Bai, Z., et al. (2020). High-Spatial-Resolution Multi-Omics Sequencing via Deterministic Barcoding in Tissue. Cell *183*, 1665–1681.e18. https://doi.org/10.1016/j.cell.2020.10.026.
13. Lee, M.Y., Bedia, J.S., Bhate, S.S., Barlow, G.L., Phillips, D., Fantl, W.J., Nolan, G.P., and Schürch, C.M. (2022). CellSeg: a robust, pre-trained nucleus segmentation and pixel quantification software for highly multiplexed fluorescence images. BMC Bioinf. *23*, 46. https://doi.org/10.1186/s12859-022-04570-9.
14. Palla, G., Spitzer, H., Klein, M., Fischer, D., Schaar, A.C., Kuemmerle, L.B., Rybakov, S., Ibarra, I.L., Holmberg, O., Virshup, I., et al. (2022). Squidpy: a scalable framework for spatial omics analysis. Nat. Methods *19*, 171–178. https://doi.org/10.1038/s41592-021-01358-2.
15. Greenwald, N.F., Miller, G., Moen, E., Kong, A., Kagel, A., Dougherty, T., Fullaway, C.C., McIntosh, B.J., Leow, K.X., Schwartz, M.S., et al. (2022). Whole-cell segmentation of tissue images with human-level performance using large-scale data annotation and deep learning. Nat. Biotechnol. *40*, 555–565. https://doi.org/10.1101/2021.03.01.431313.
16. Schapiro, D., Jackson, H.W., Raghuraman, S., Fischer, J.R., Zanotelli, V.R.T., Schulz, D., Giesen, C., Catena, R., Varga, Z., and Bodenmiller, B. (2017). HistoCAT: Analysis of cell phenotypes and interactions in multiplex image cytometry data. Nat. Methods *14*, 873–876. https://doi.org/10.1038/nmeth.4391.
17. Pham, D., Tan, X., Xu, J., Grice, L.F., Lam, P.Y., Raghubar, A., Vukovic, J., Ruitenberg, M.J., and Nguyen, Q. (2022). stLearn: integrating spatial location, tissue morphology and gene expression to find cell types, cell-cell interactions and spatial trajectories within undissociated tissues. Preprint at BioRxiv. https://doi.org/10.1101/2020.05.31.125658.
18. Dries, R., Zhu, Q., Dong, R., Eng, C.H.L., Li, H., Liu, K., Fu, Y., Zhao, T., Sarkar, A., Bao, F., et al. (2021). Giotto: a toolbox for integrative analysis and visualization of spatial expression data. Genome Biol. *22*, 78. https://doi.org/10.1186/s13059-021-02286-2.
19. Palla, G., Fischer, D.S., Regev, A., and Theis, F.J. (2022). Spatial components of molecular tissue biology. Nat. Biotechnol. *40*, 308–318. Nature Research. https://doi.org/10.1038/s41587-021-01182-1.
20. Schürch, C.M., Bhate, S.S., Barlow, G.L., Phillips, D.J., Noti, L., Zlobec, I., Chu, P., Black, S., Demeter, J., McIlwain, D.R., et al. (2020). Coordinated Cellular Neighborhoods Orchestrate Antitumoral Immunity at the Colorectal Cancer Invasive. Cell *182*, 1341–1359.e19. https://doi.org/10.1016/j.cell.2020.07.005.
21. Lecun, Y., Bengio, Y., and Hinton, G. (2015). Deep learning. Nature *521*, 436–444. https://doi.org/10.1038/nature14539.
22. Damond, N., Engler, S., Zanotelli, V.R.T., Schapiro, D., Wasserfall, C.H., Kusmartseva, I., Nick, H.S., Thorel, F., Herrera, P.L., Atkinson, M.A., and Bodenmiller, B. (2019). A Map of Human Type 1 Diabetes Progression by Imaging Mass Cytometry. Cell Metab. *29*, 755–768.e5. https://doi.org/10.1016/j.cmet.2018.11.014.
23. Arvaniti, E., and Claassen, M. (2017). Sensitive detection of rare disease-Associated cell subsets via representation learning. Nat. Commun. *8*, 14825. https://doi.org/10.1038/ncomms14825.
24. Nearchou, I.P., Gwyther, B.M., Georgiakakis, E.C.T., Gavriel, C.G., Lillard, K., Kajiwara, Y., Ueno, H., Harrison, D.J., and Caie, P.D. (2020). Spatial immune profiling of the colorectal tumor microenvironment predicts good outcome in stage II patients. NPJ Digit. Med. *3*, 71. https://doi.org/10.1038/s41746-020-0275-x.
25. Galon, J., Costes, A., Sanchez-Cabo, F., Kirilovsky, A., Mlecnik, B., Lagorce-Pagès, C., Tosolini, M., Camus, M., Berger, A., Wind, P., et al. (2006). Type, density, and location of immune cells within human colorectal tumors predict clinical outcome. Science *313*, 1960–1964. https://doi.org/10.1126/science.1129139.
26. di Caro, G., Bergomas, F., Grizzi, F., Doni, A., Bianchi, P., Malesci, A., Laghi, L., Allavena, P., Mantovani, A., and Marchesi, F. (2014). Occurrence of tertiary lymphoid tissue is associated with T-cell infiltration and predicts better prognosis in early-stage colorectal cancers. Clin. Cancer Res. *20*, 2147–2158. https://doi.org/10.1158/1078-0432.CCR-13-2590.

27. Cohen, R.B. (2003). Epidermal growth factor receptor as a therapeutic target in colorectal cancer. Clin. Colorectal Cancer *2*, 246–251. https://doi.org/10.3816/CCC.2003.n.006.

28. Atkinson, M.A., Eisenbarth, G.S., and Michels, A.W. (2014). Type 1 diabetes. Lancet *383*, 69–82. https://doi.org/10.1016/S0140-6736(13)60591-7.

29. Bluestone, J.A., Herold, K., and Eisenbarth, G. (2010). Genetics, pathogenesis and clinical interventions in type 1 diabetes. Nature *464*, 1293–1300. https://doi.org/10.1038/nature08933.

30. Boldison, J., and Wong, F.S. (2016). Immune and Pancreatic β Cell Interactions in Type 1 Diabetes. Trends Endocrinol. Metab. *27*, 856–867. https://doi.org/10.1016/j.tem.2016.08.007.

31. Ruf, B., Bruhns, M., Babaei, S., Kedei, N., Ma, L., Revsine, M., Benmebarek, M.-R., Ma, C., Heinrich, B., Subramanyam, V., et al. (2023). Tumor-associated macrophages trigger MAIT cell dysfunction at the HCC invasive margin. Cell *186*. Published online August 17, 2023. https://doi.org/10.1016/j.cell.2023.07.026.

Patterns, Volume *4*

Supplemental information

S^3-CIMA: Supervised spatial single-cell image analysis for identifying disease-associated cell-type compositions in tissue

Sepideh Babaei, Jonathan Christ, Vivek Sehra, Ahmad Makky, Mohammed Zidane, Kilian Wistuba-Hamprecht, Christian Schürch, and Manfred Claassen

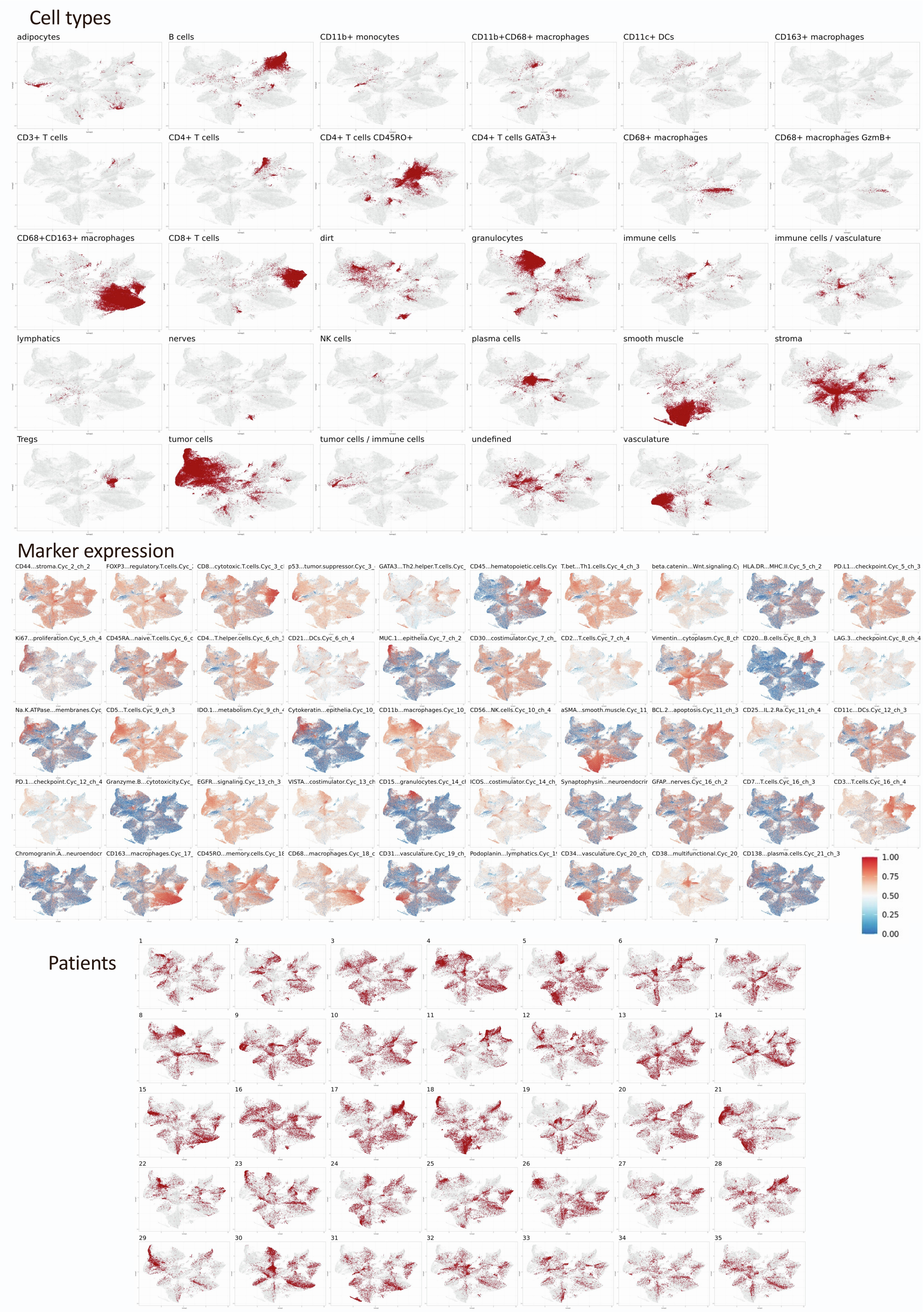

Fig. S1.

CRC data

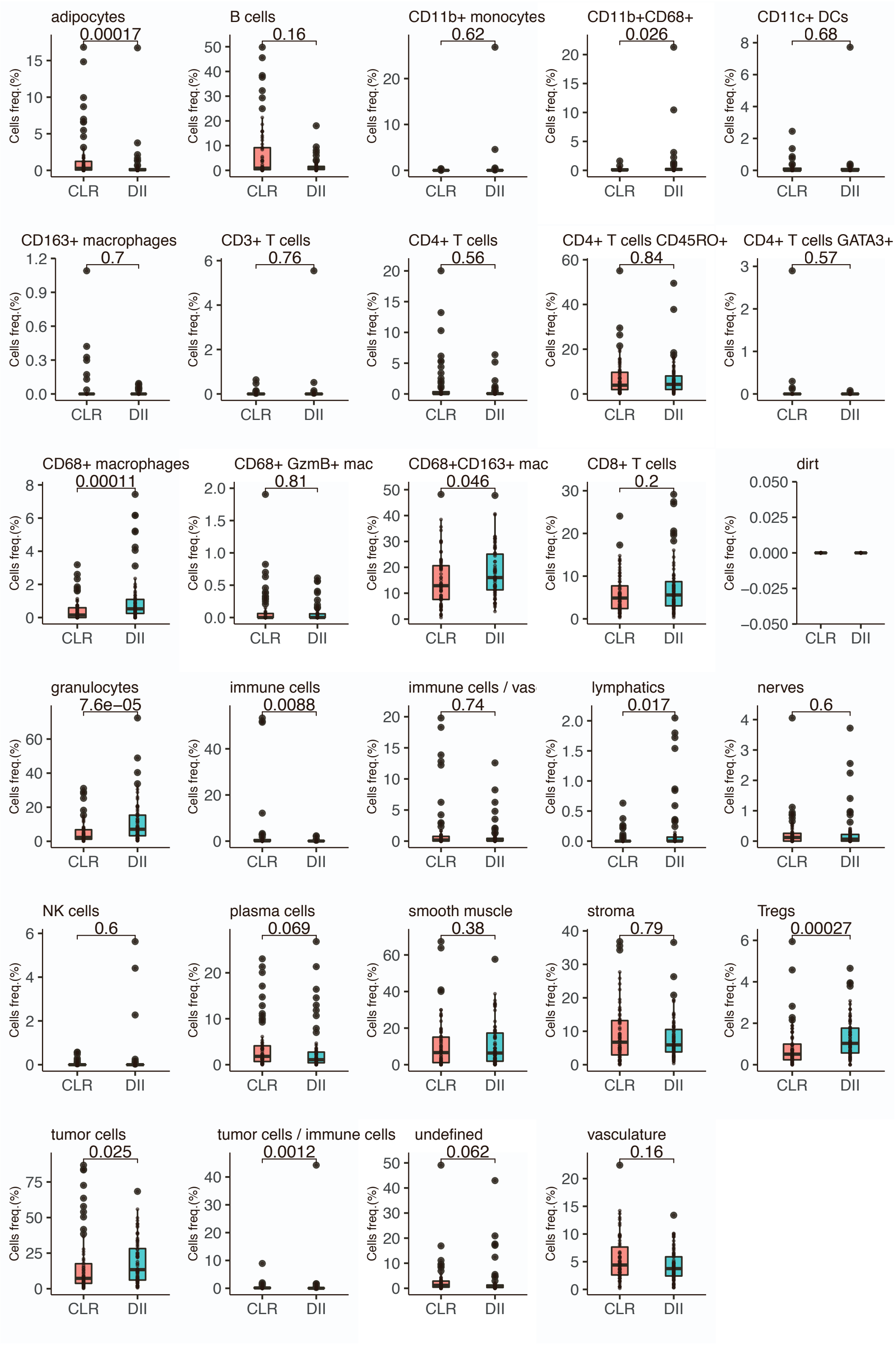

Fig. S2.

Box plot of frequency of each cell type in each CRC group (CLR vs DII)

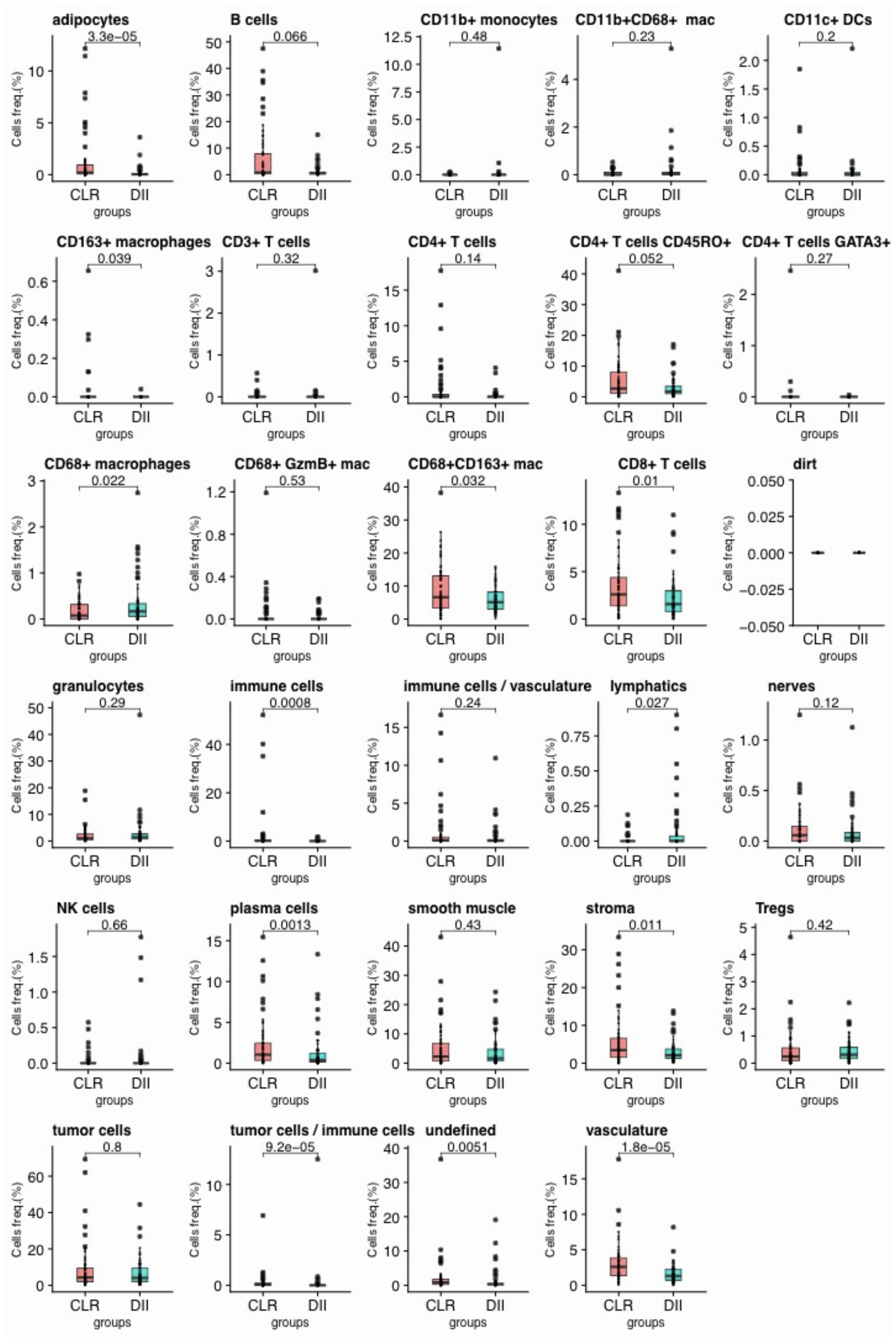

Fig. S3.

Box plot of frequency of selected cell obtained from S[3]-CIMA global enrichment analysis at k = 30 in each CRC group per cell type.

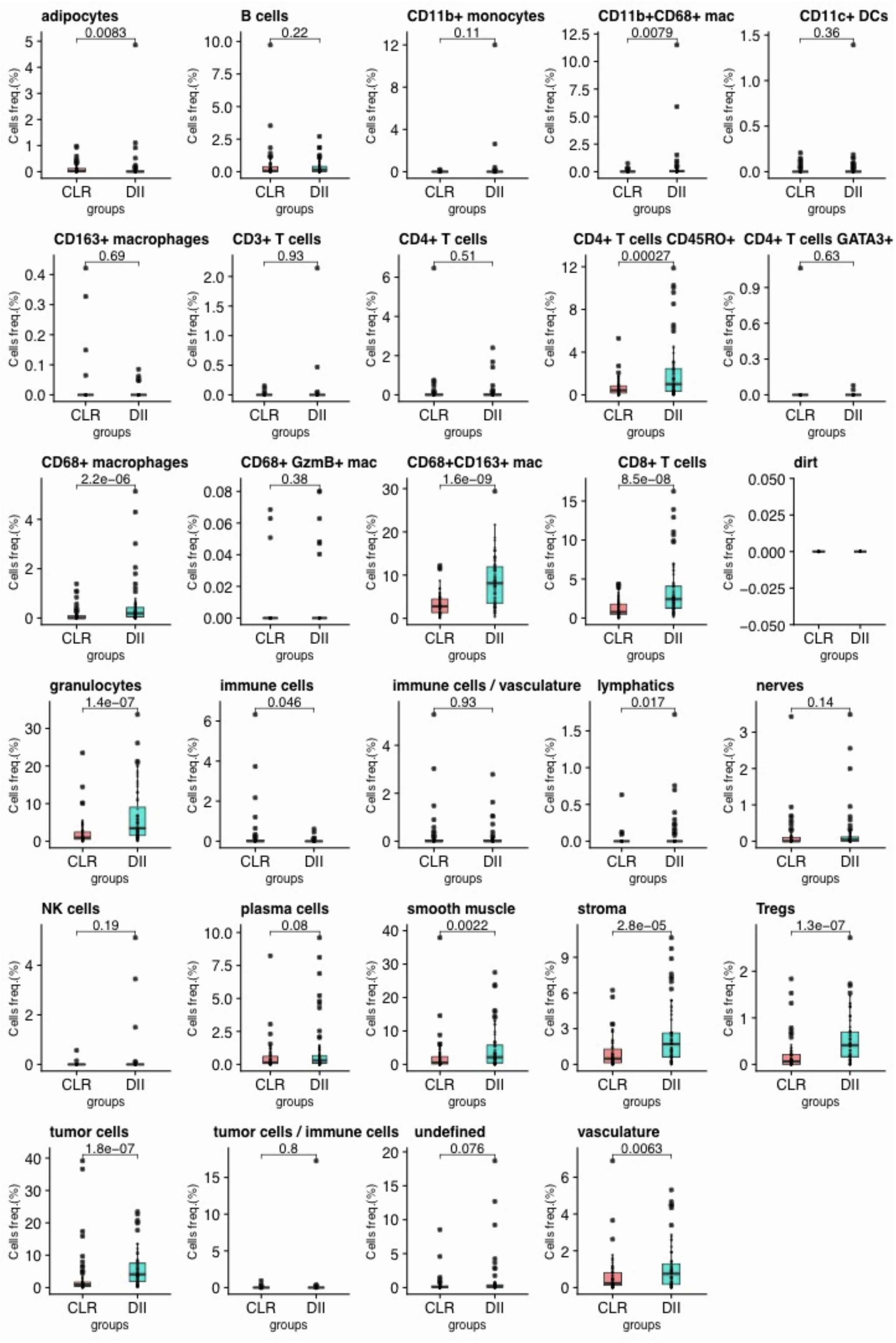

Fig. S4.

Box plot of frequency of selected cell obtained from S^3-CIMA global enrichment analysis at k = 50 in each CRC group per cell type.

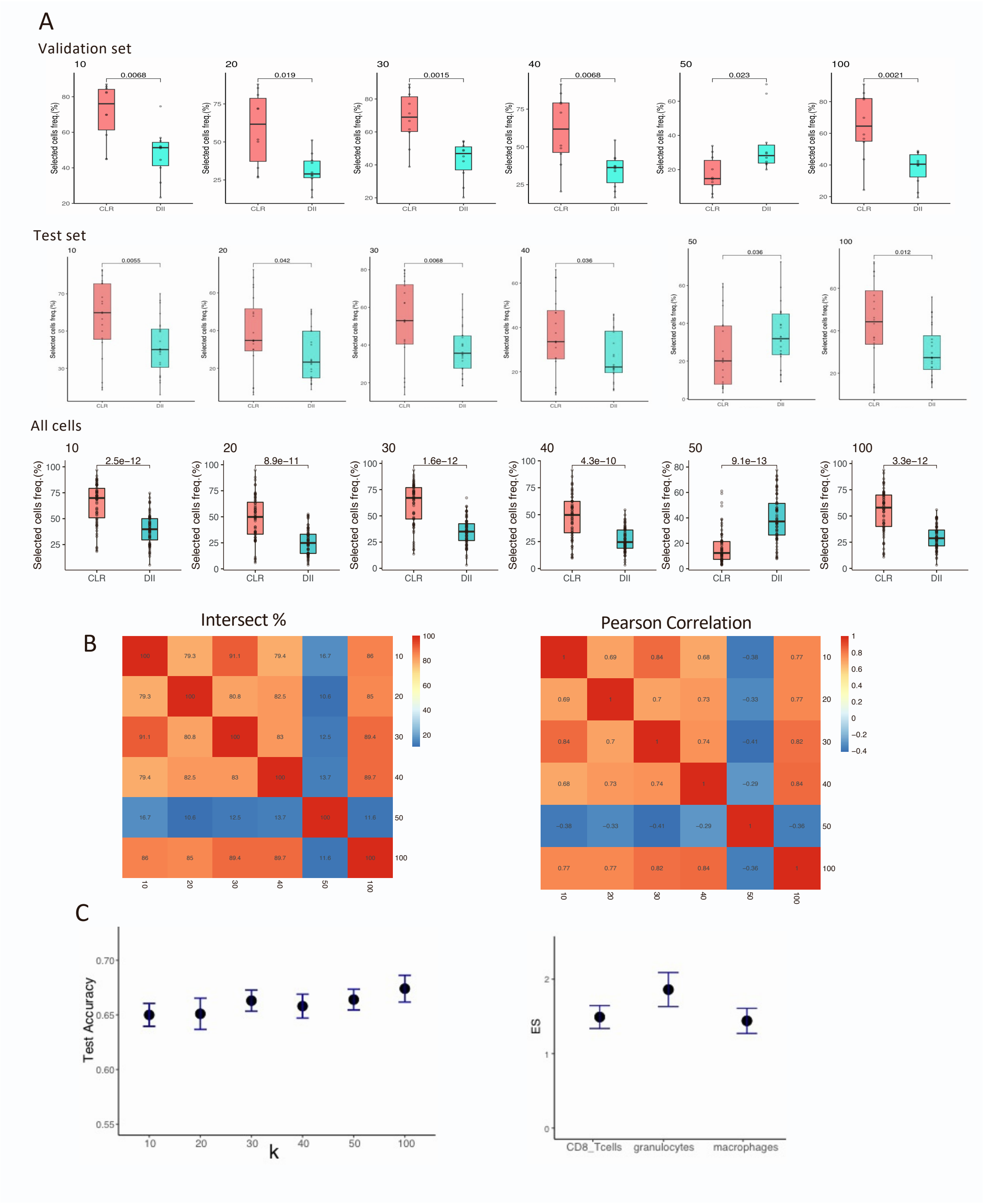

Fig. S5.

The frequency of selected cells between groups using S[3]-CIMA global enrichment analysis across different cell neighborhood sizes (10 to 100) using different subset of cells. Heatmaps show the overlap (%) and correlation between the set of selected cells over the different neighborhood sizes.

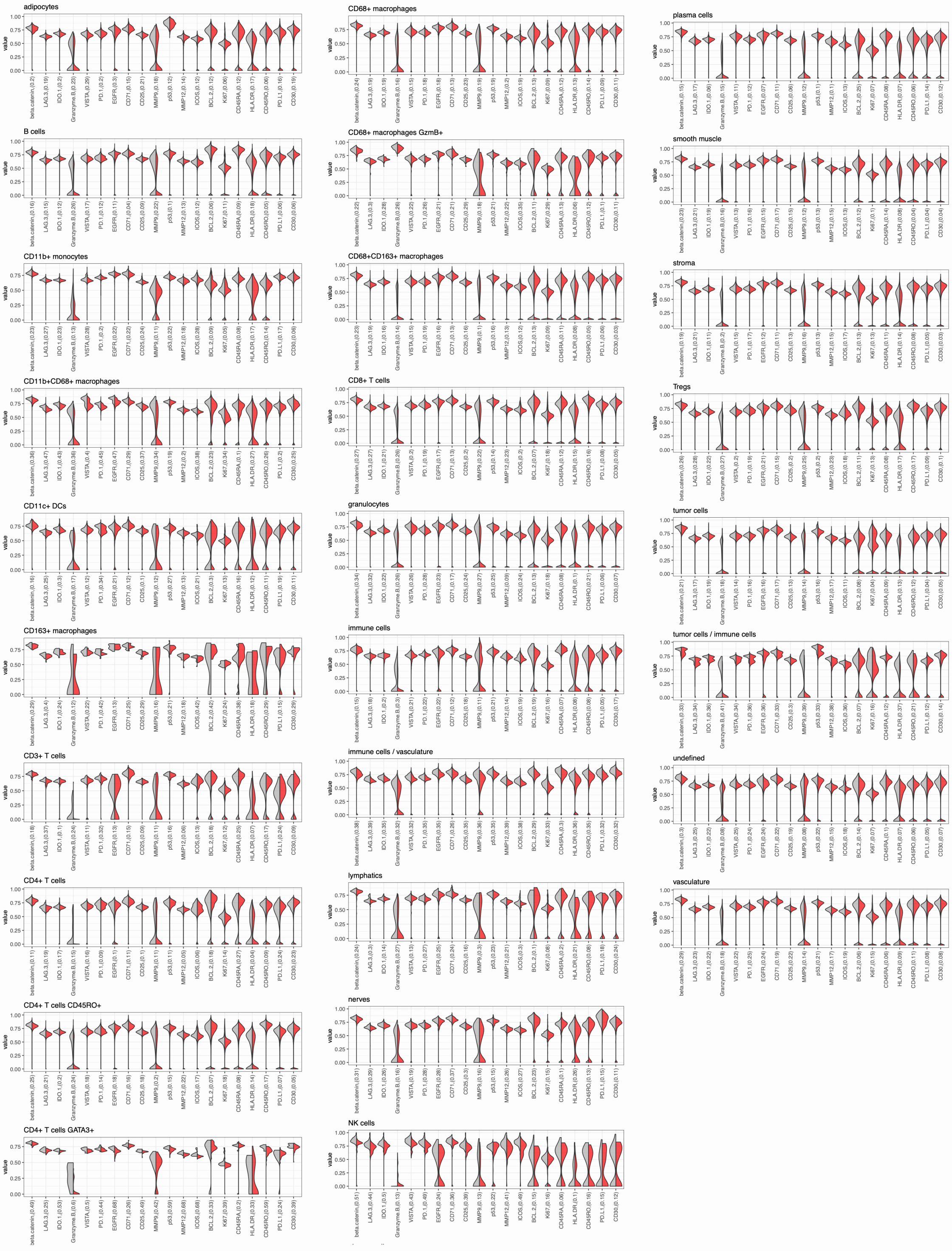

Fig. S6.

S^3-CIMA global enrichment analysis at k =30, Density of functional marker expression showing greatest differential abundance in terms of the Kolmogorov–Smirnov two-sample test between the selected and non-selected cell subsets per cell types.

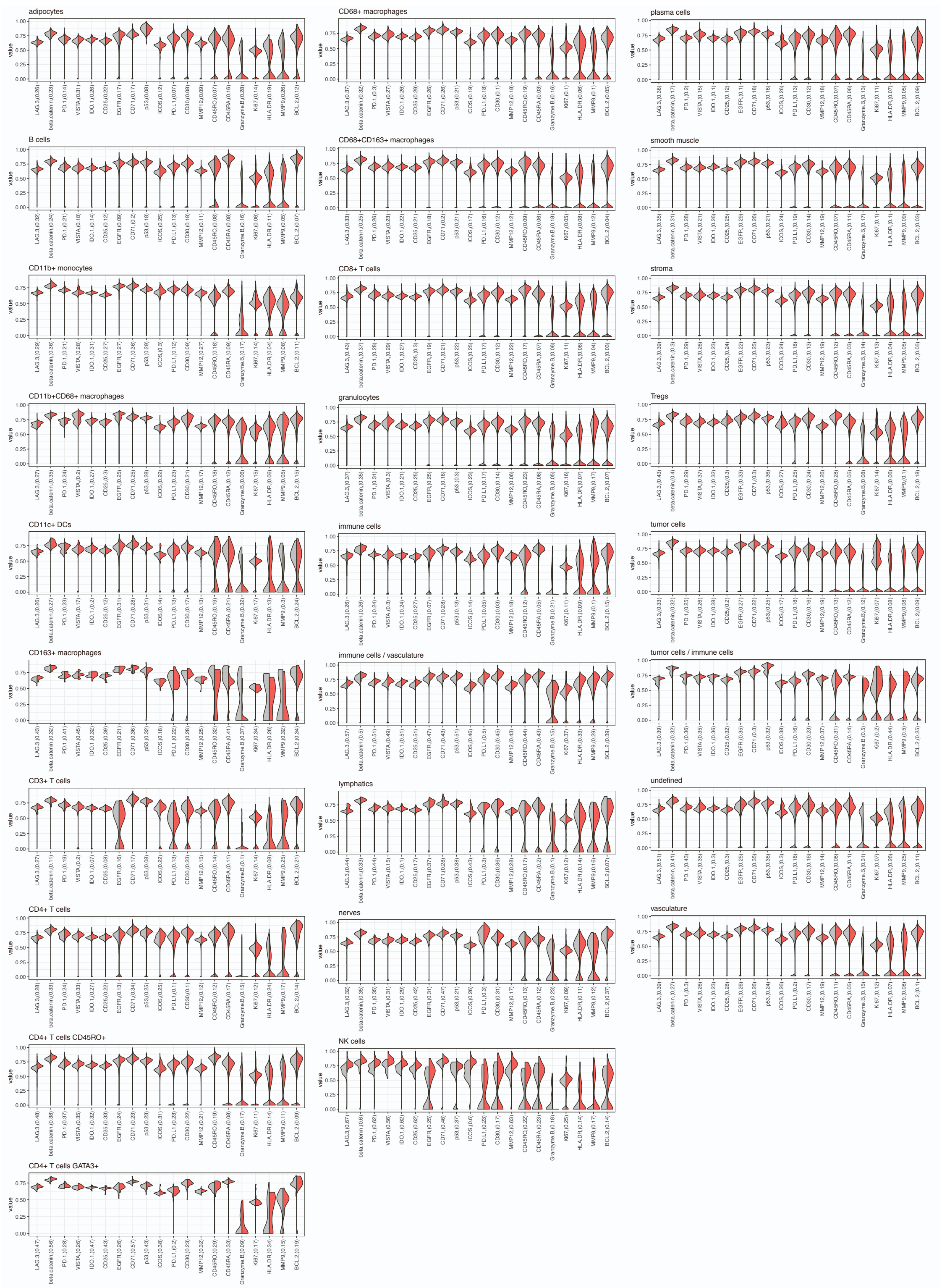

Fig. S7.

S[3]-CIMA global enrichment analysis at k =50, Density of functional marker expression showing greatest differential abundance in terms of the Kolmogorov–Smirnov two-sample test between the selected and non-selected cell subsets per cell types.

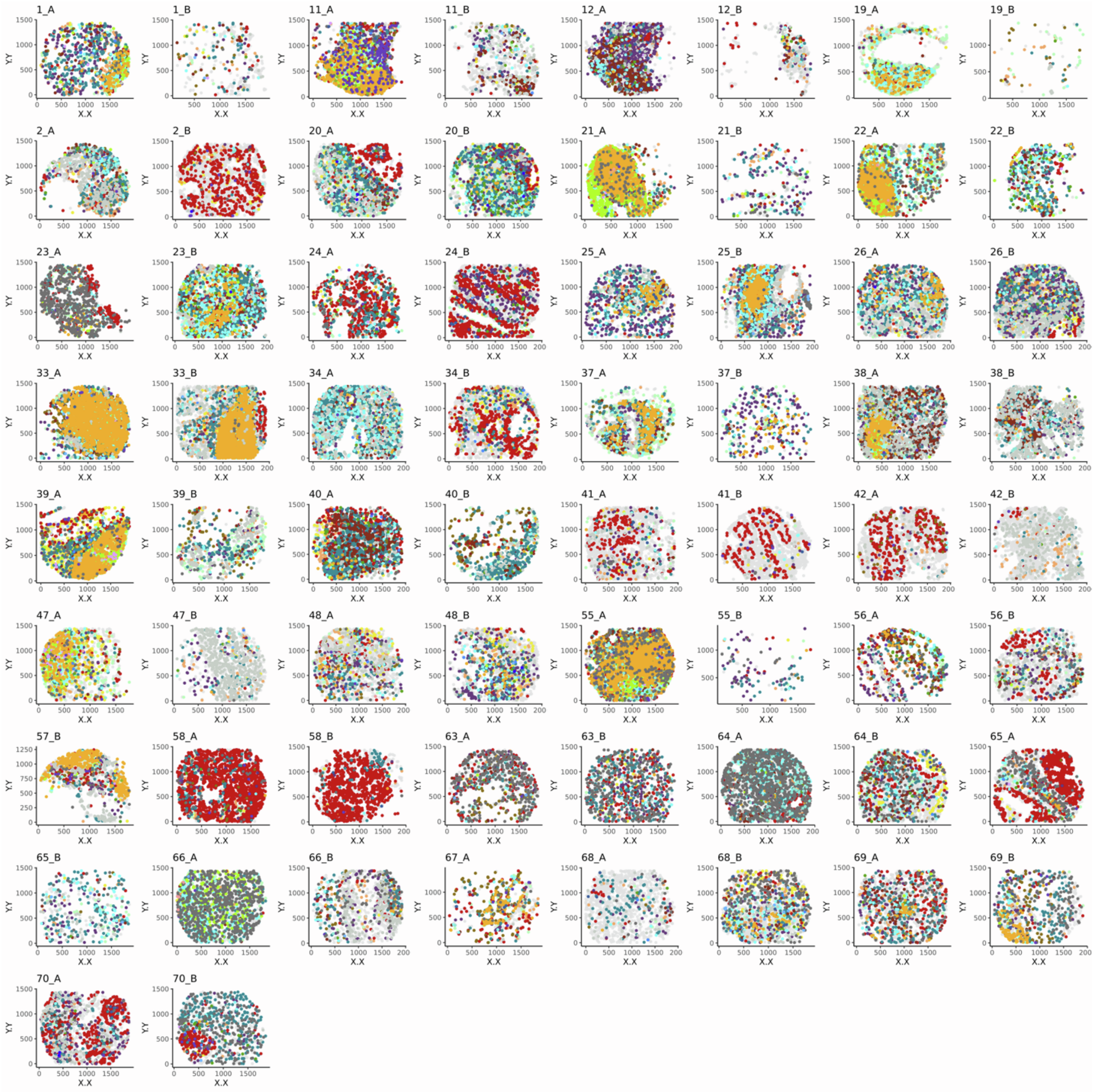

Fig. S8.

S^3-CIMA global enrichment analysis at k =30, Selected cells (colored by cell type) are mapped back to the corresponding patient CODEX images in both CLR group.

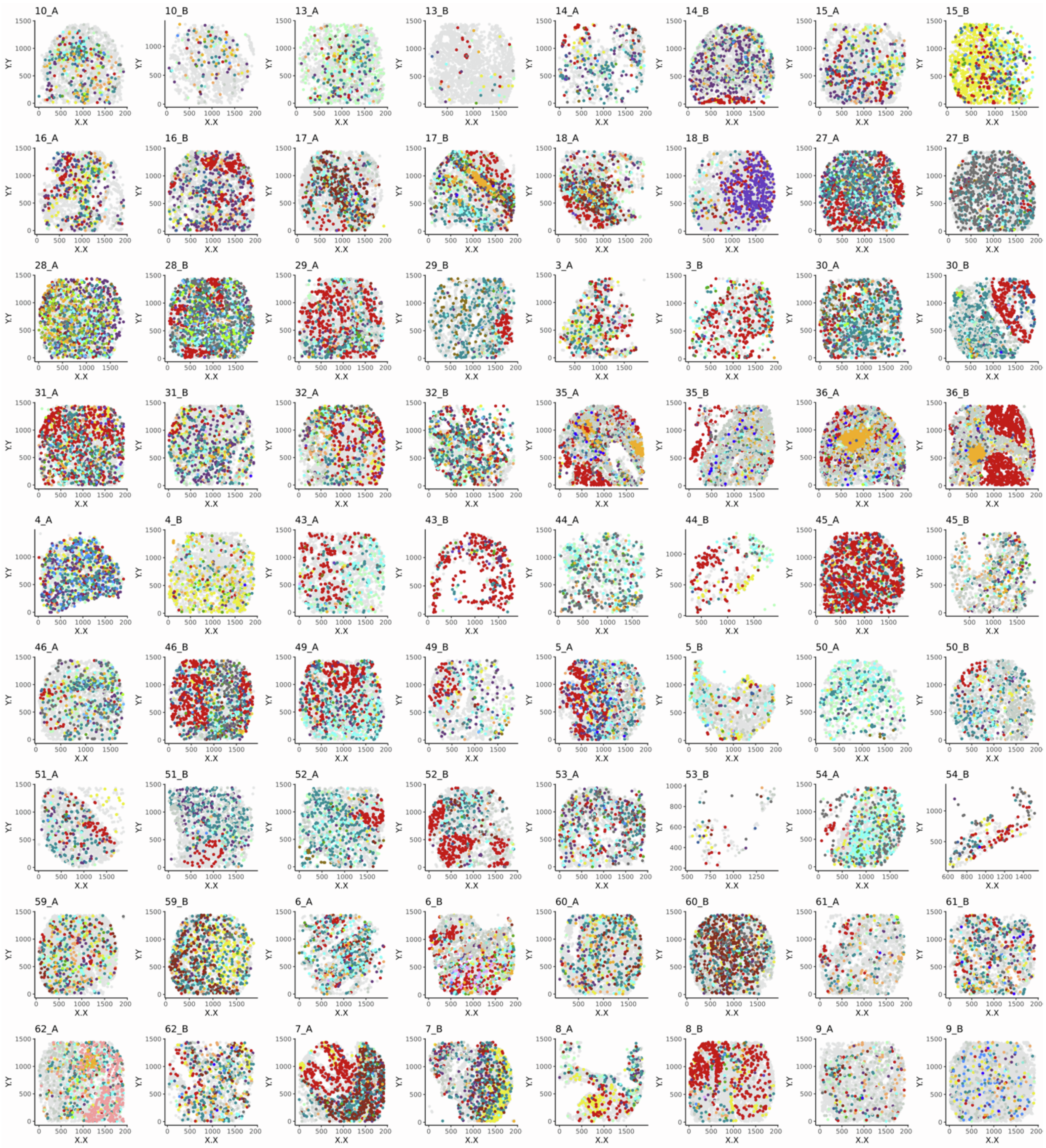

Fig. S9.

S3-CIMA global enrichment analysis at k =30, Selected cells (colored by cell type) are mapped back to the corresponding patient CODEX images in both DII group.

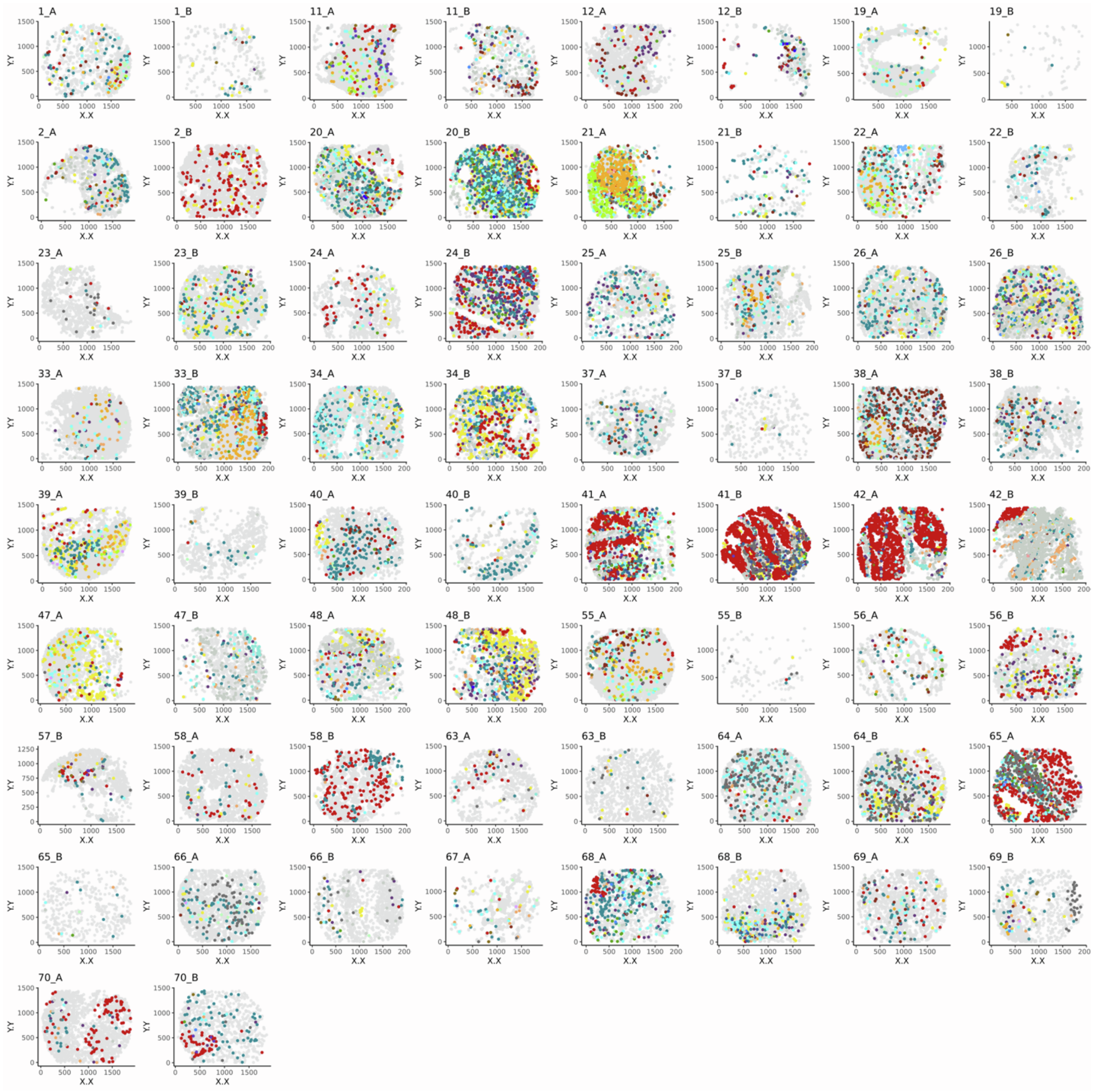

Fig. S10.

S^3-CIMA global enrichment analysis at k =50, Selected cells (colored by cell type) are mapped back to the corresponding patient CODEX images in both CLR group.

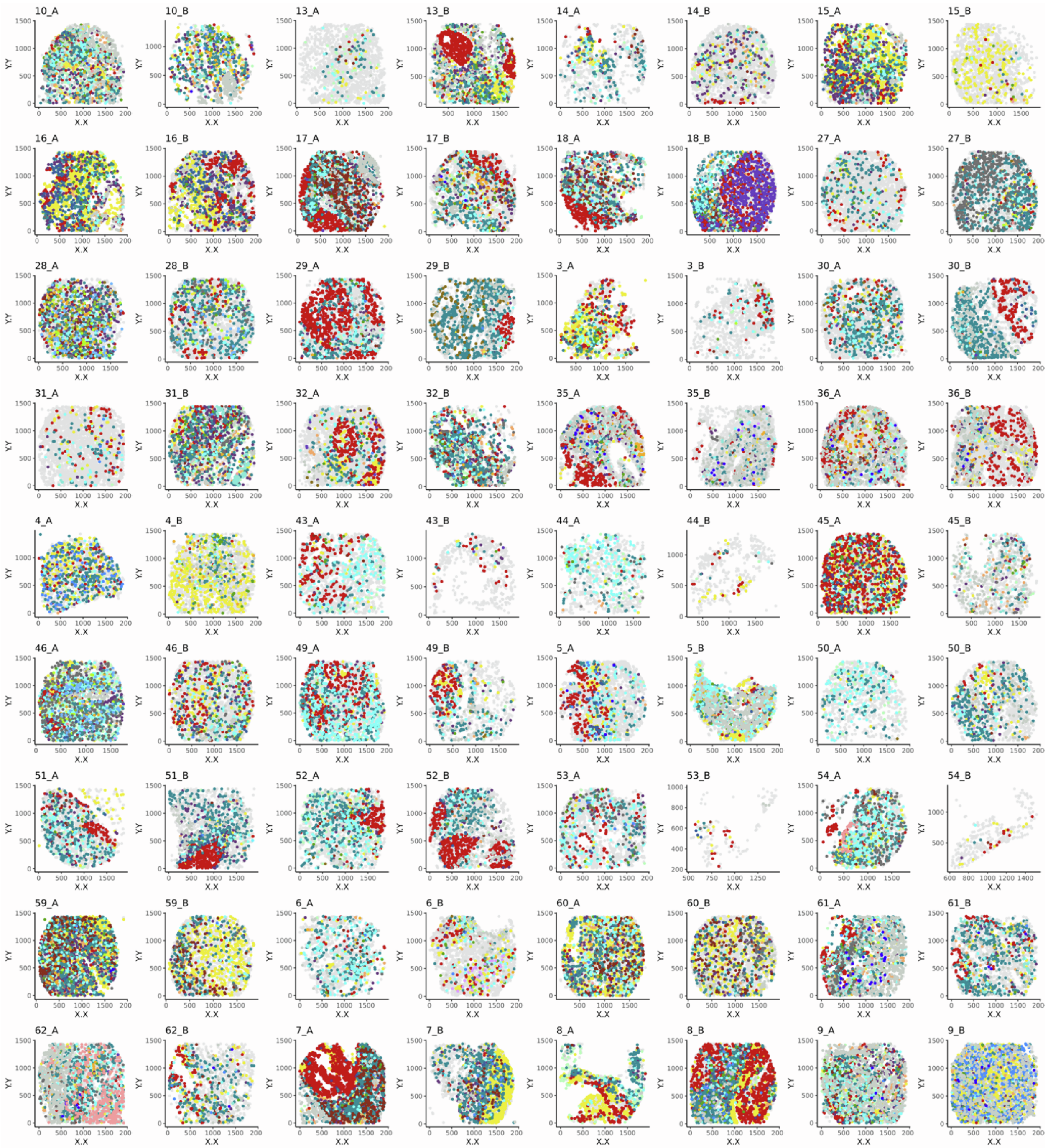

Fig. S11.

S[3]-CIMA global enrichment analysis at k =50, Selected cells (colored by cell type) are mapped back to the corresponding patient CODEX images in both DII group.

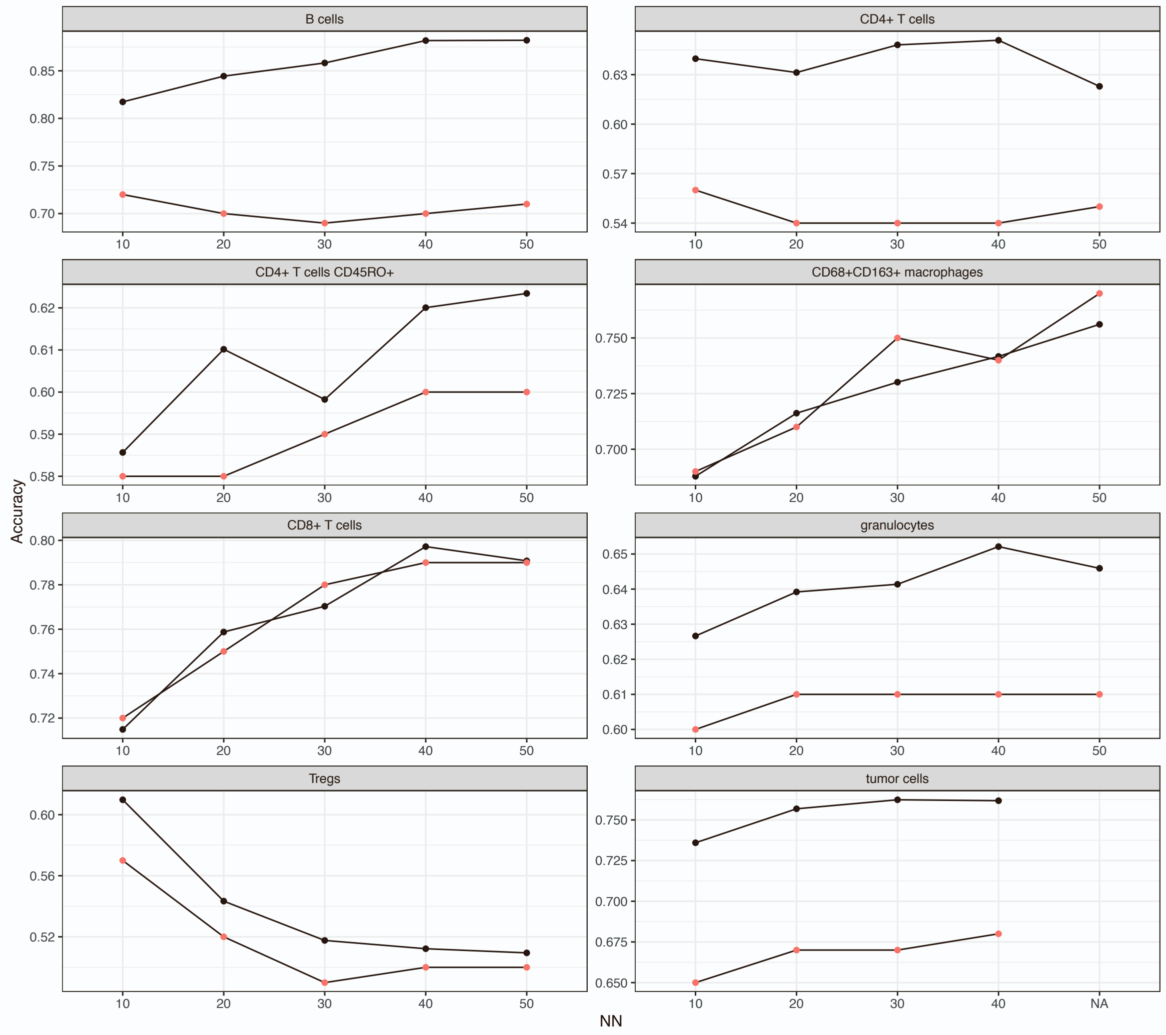

Fig. S12.

S^3-CIMA anchor based spatial enrichment analysis classification performance (test (red) and train (black) accuracy) across different cell neighborhood sizes (10 to 50).

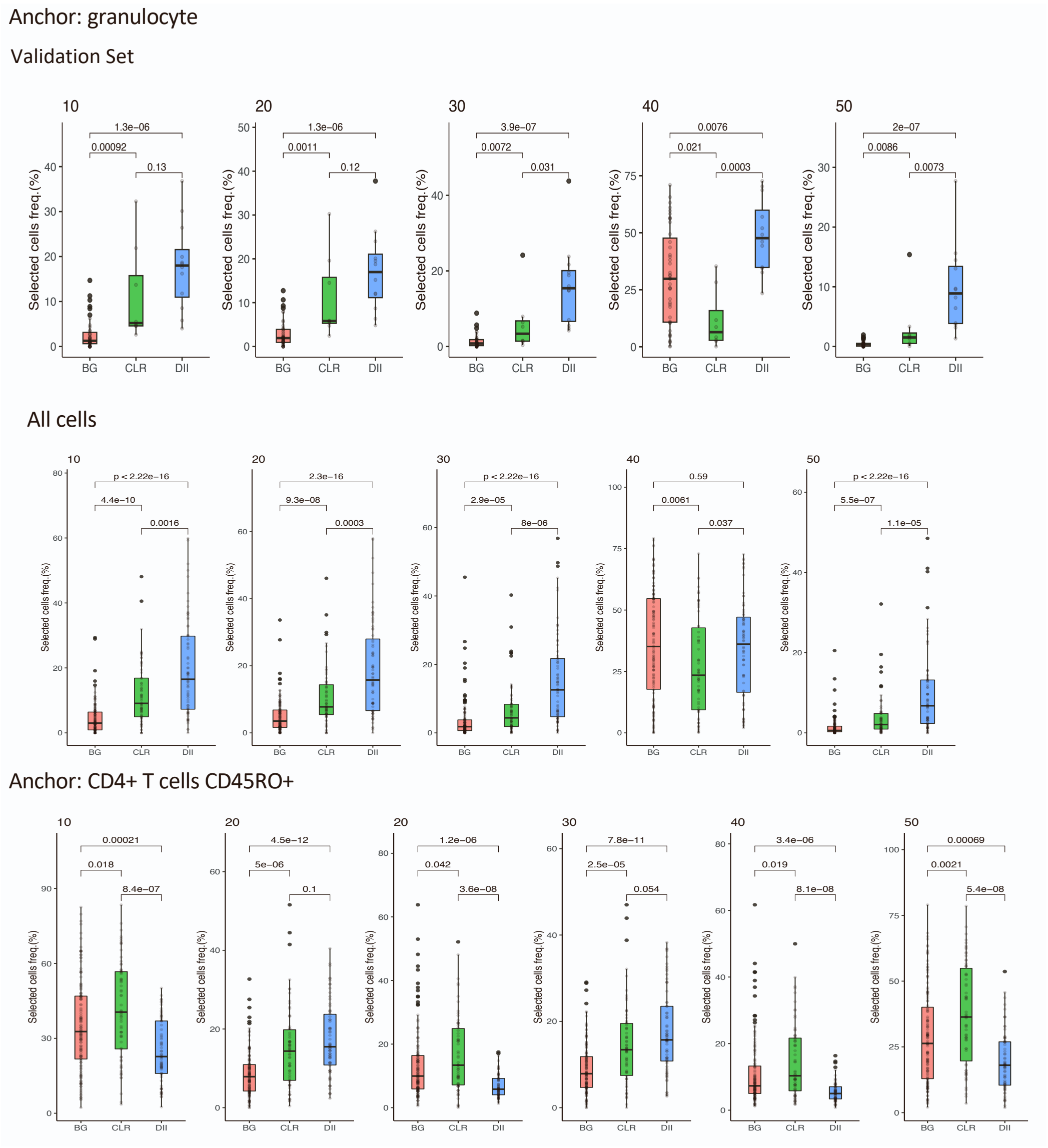

Fig. S13.

Boxplots of selected cell type frequency of two CRC groups using S^3-CIMA anchor based spatial enrichment analysis with granulocyte and CD4+ T cells CD45RO+ as the anchor.

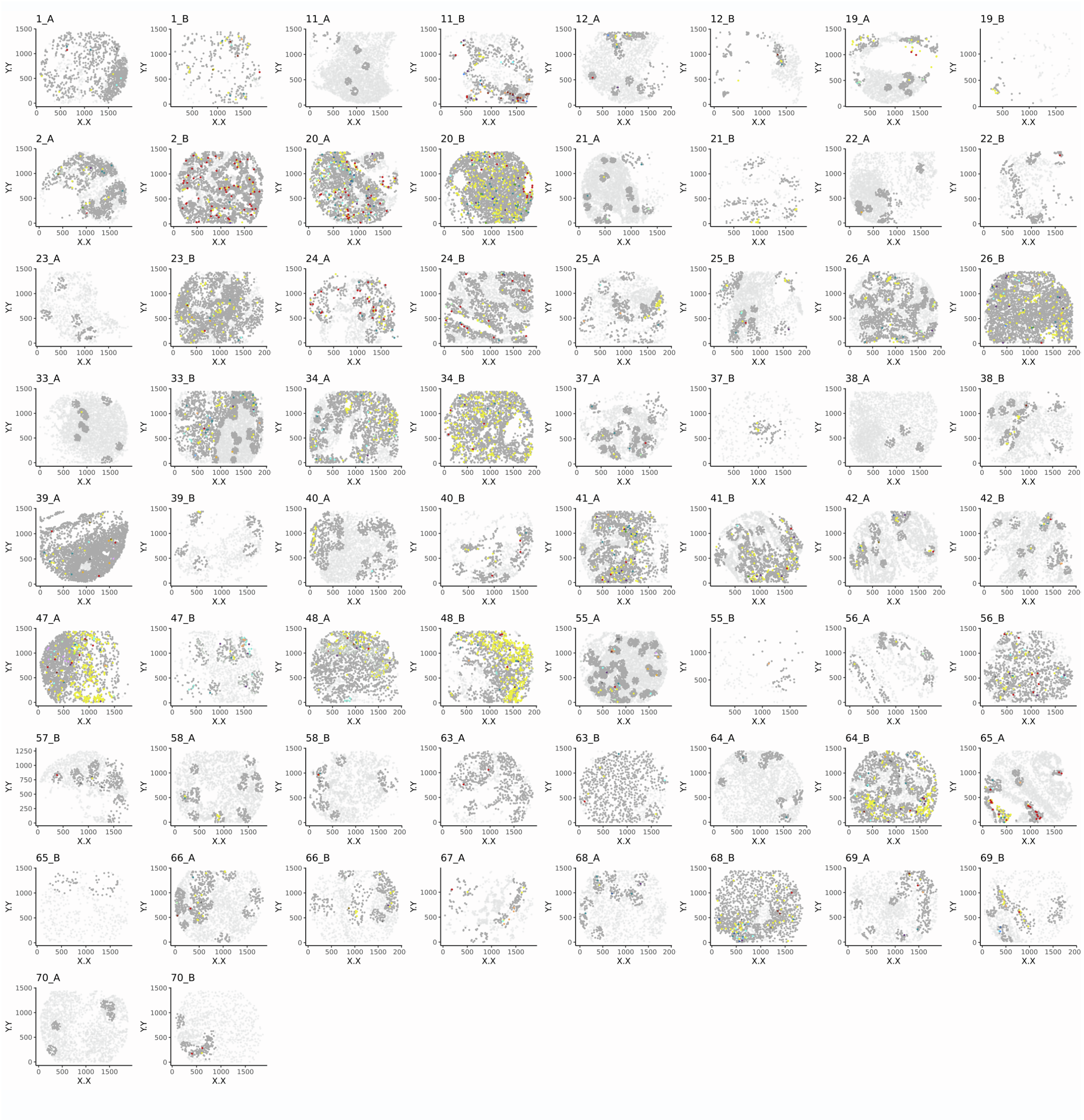

Fig. S14.

Selected cells (colored by cell type) using S[3]-CIMA anchor based spatial enrichment analysis with granulocyte as the anchor mapped back to the corresponding patient CODEX images in the **CLR** group.

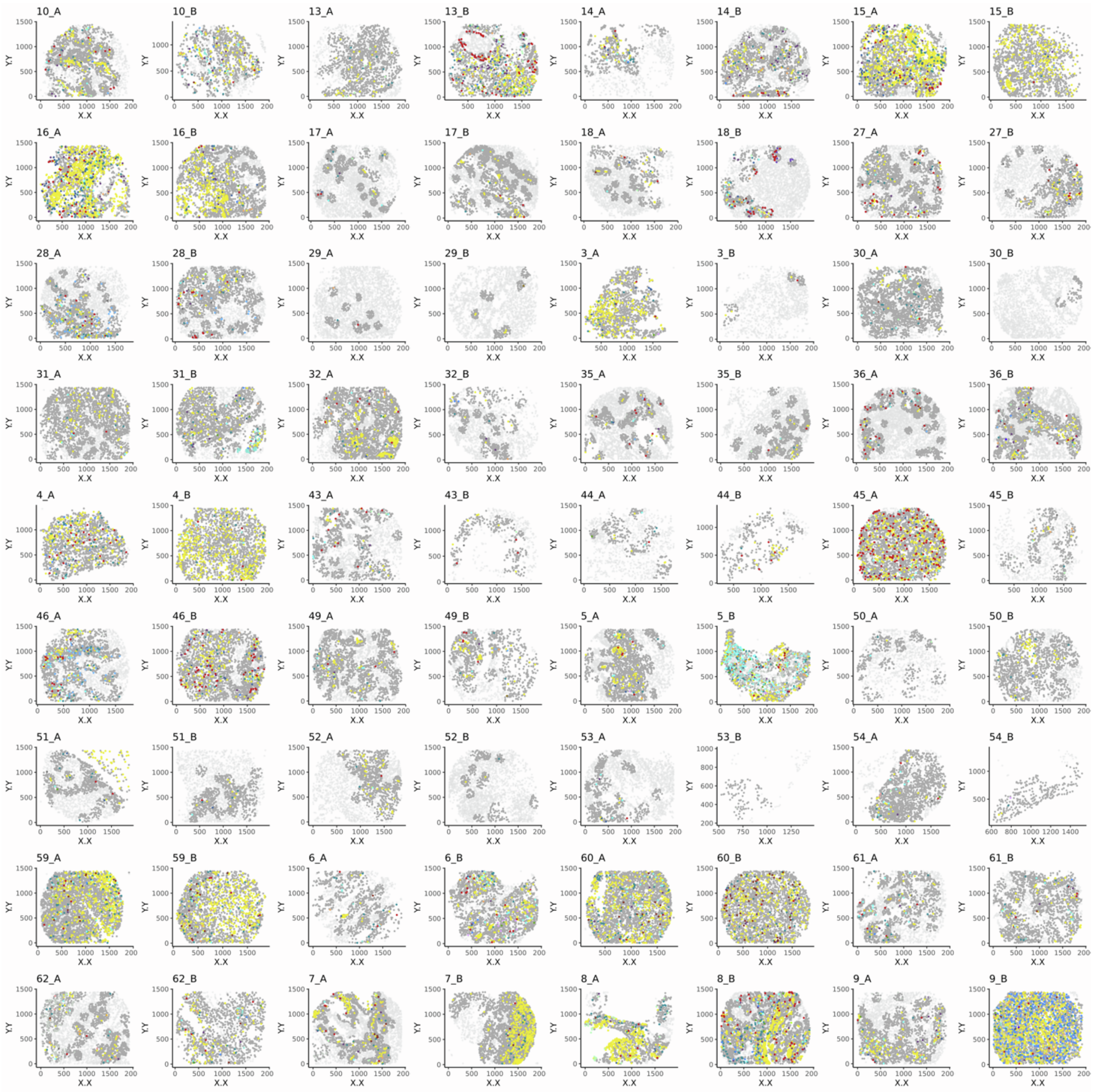

Fig. S15.

Selected cells (colored by cell type) using S^3-CIMA anchor based spatial enrichment analysis with granulocyte as the anchor mapped back to the corresponding patient CODEX images in the **DII** group.

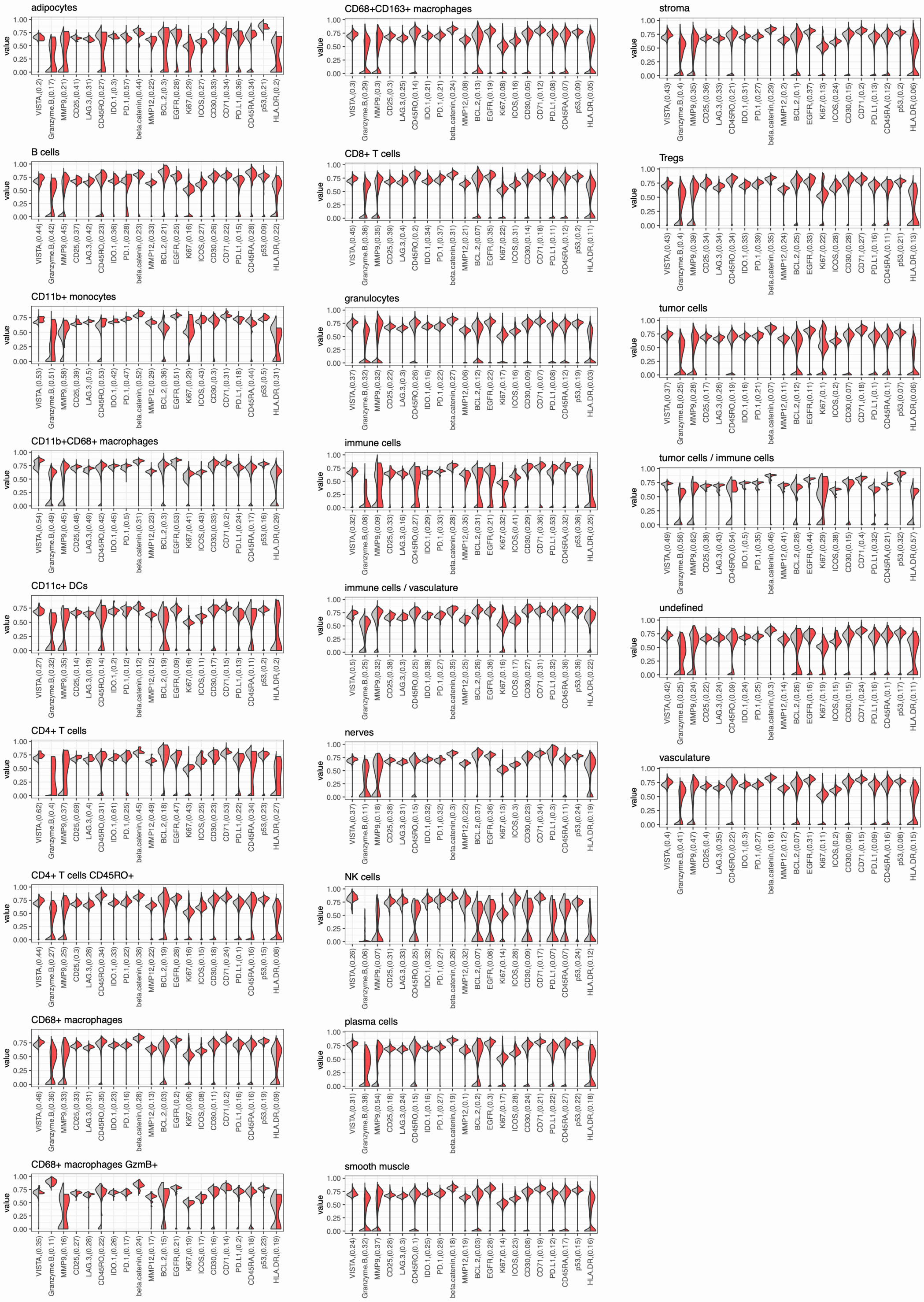

Fig. S16.

S[3]-CIMA local enrichment analysis at k =30 granulocyte as the anchor, Density of functional marker expression showing differential abundance (KS two-sample test) between the selected and non-selected cells per cell types.

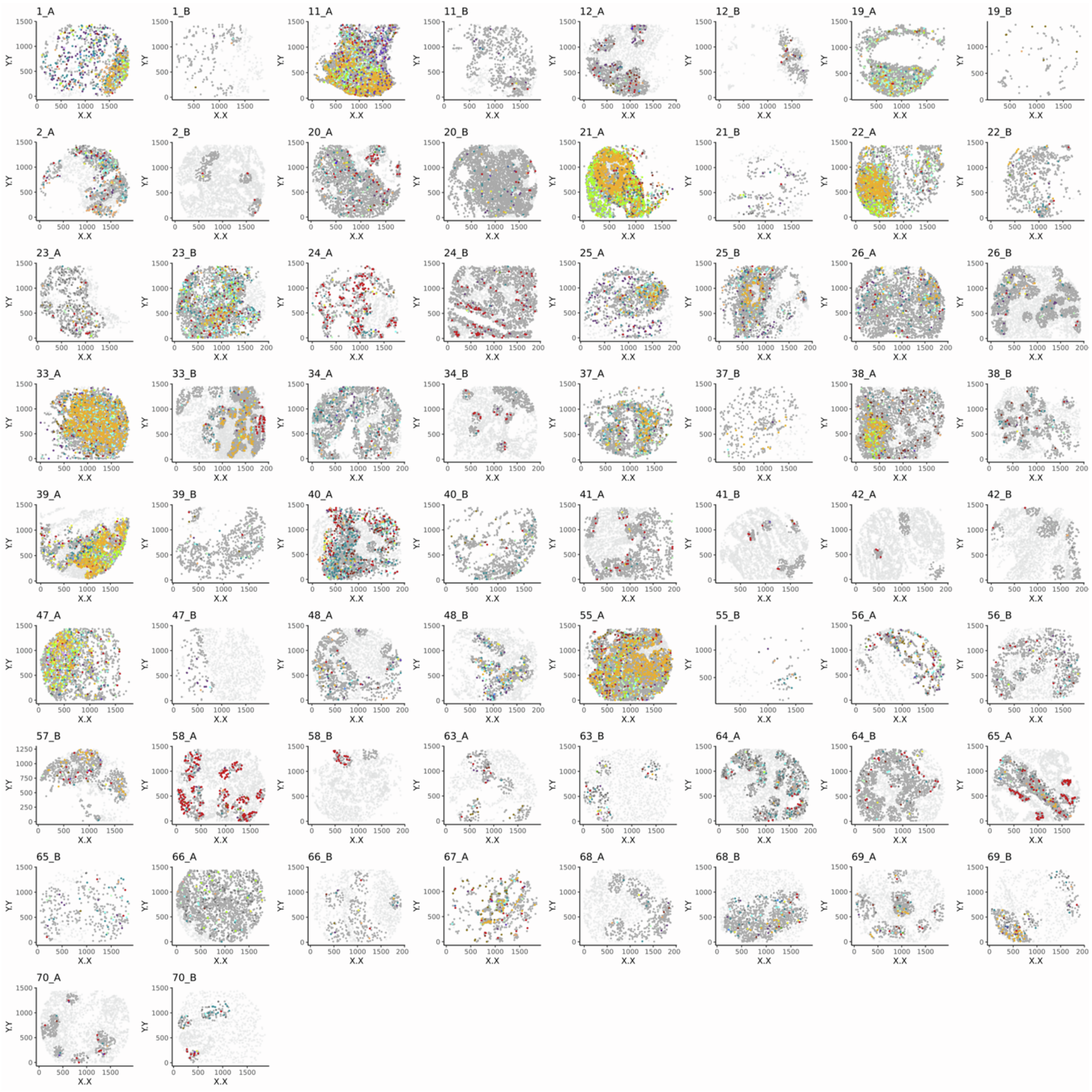

Fig. S17.

Selected cells (colored by cell type) using S^3-CIMA anchor based spatial enrichment analysis with CD4+ T cells CD45RO+ as the anchor mapped back to the corresponding patient CODEX images in the **CLR** group.

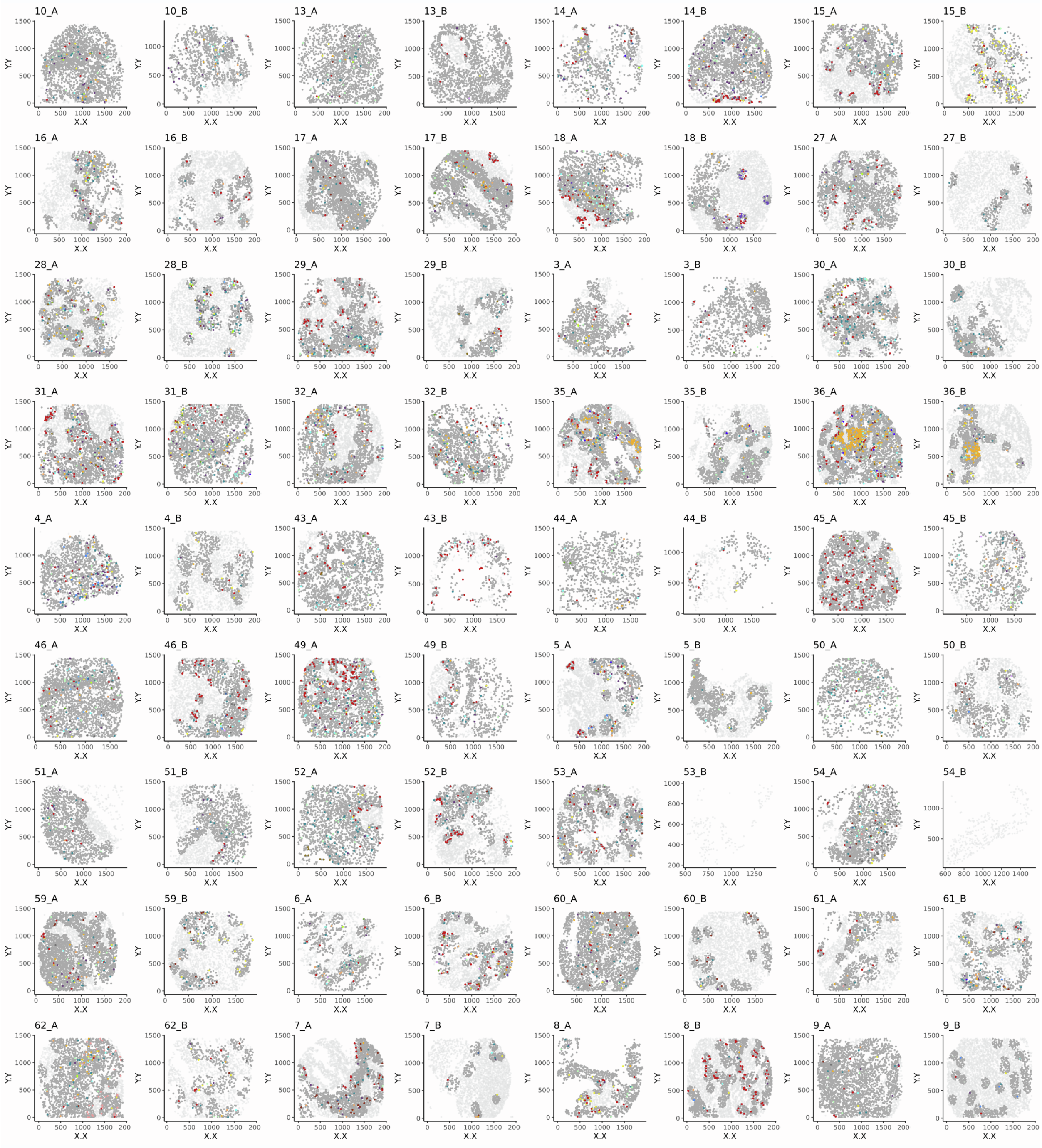

Fig. S18.

Selected cells (colored by cell type) using S^3-CIMA anchor based spatial enrichment analysis with CD4+ T cells CD45RO+ as the anchor mapped back to the corresponding patient CODEX images in the **DII** group.

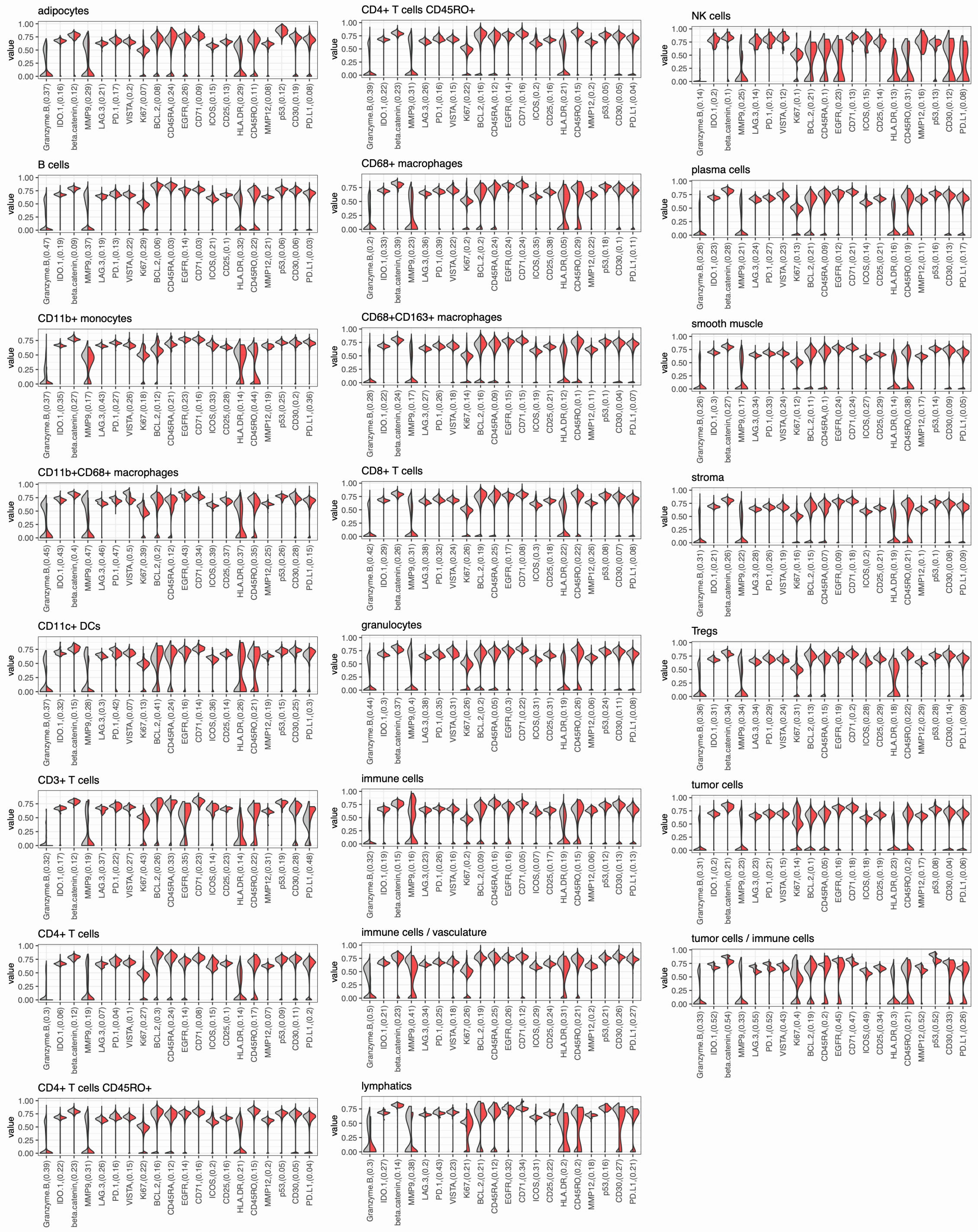

Fig. S19.

S^3-CIMA local enrichment analysis at k =40, CD4+ T cells CD45RO+ as the anchor, Density of functional marker expression showing differential abundance (KS two-sample test) between the selected and non-selected cells per cell types.

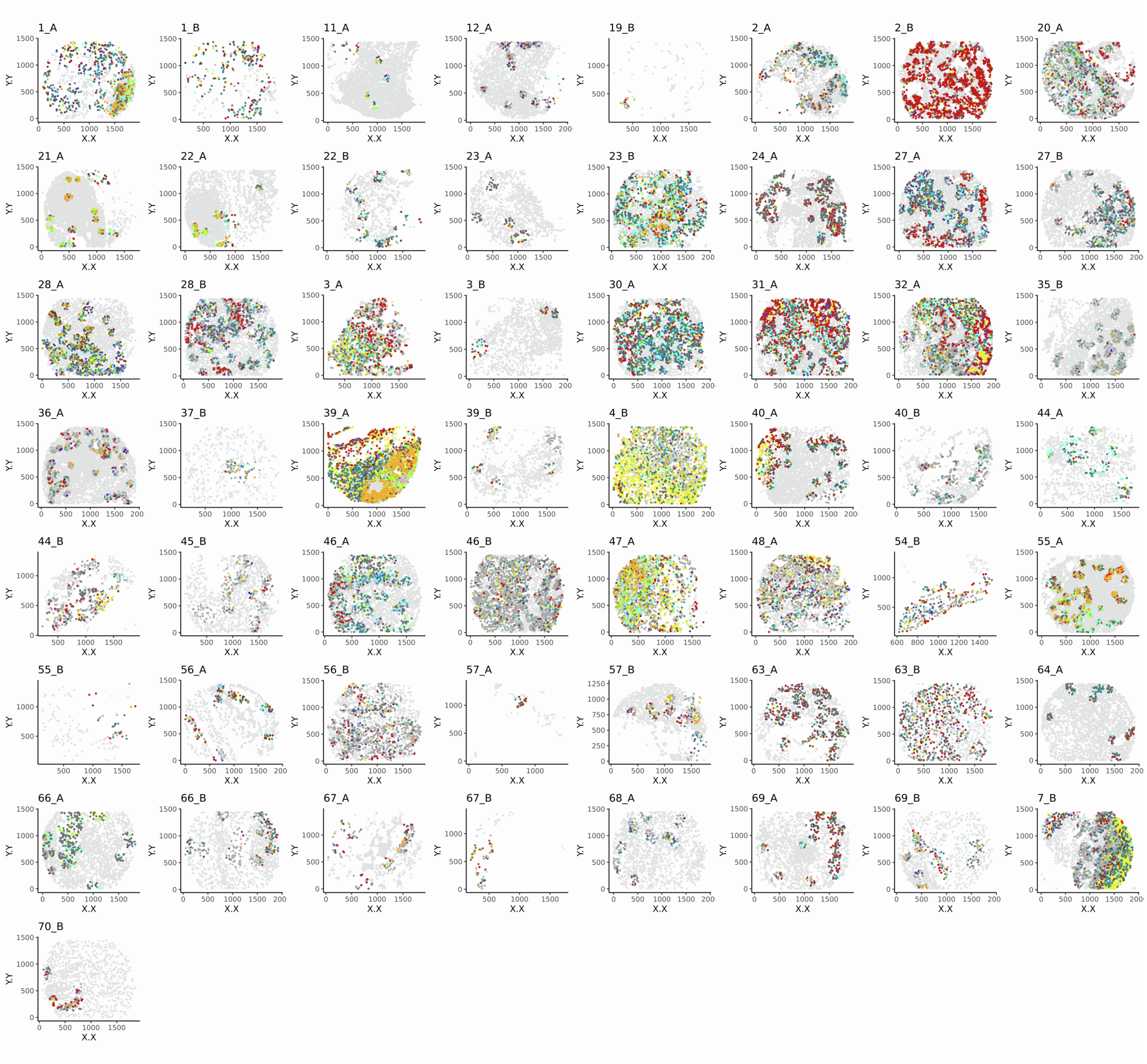

Fig. S20.

Selected cells (colored by cell type) using S^3-CIMA functional spatial enrichment analysis with granulocytes as the anchor and EGFR marker expression as the phenotype mapped back to the corresponding patient CODEX images in the **EGFR low** group. (Filter 1).

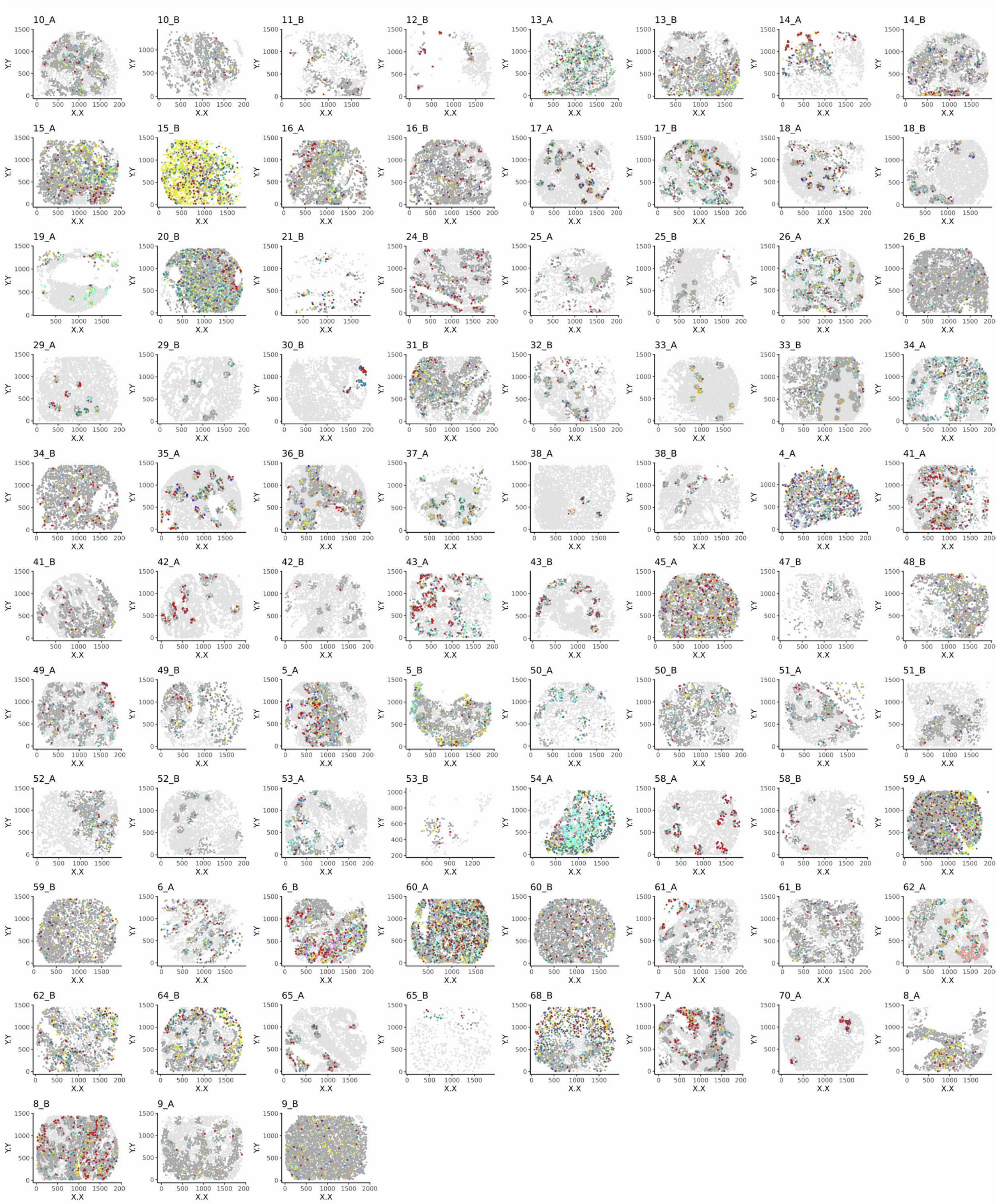

Fig. S21.

Selected cells (colored by cell type) using S^3-CIMA functional spatial enrichment analysis with granulocytes as the anchor and EGFR marker expression as the phenotype mapped back to the corresponding patient CODEX images in the **EGFR high** group. (Filter 1).

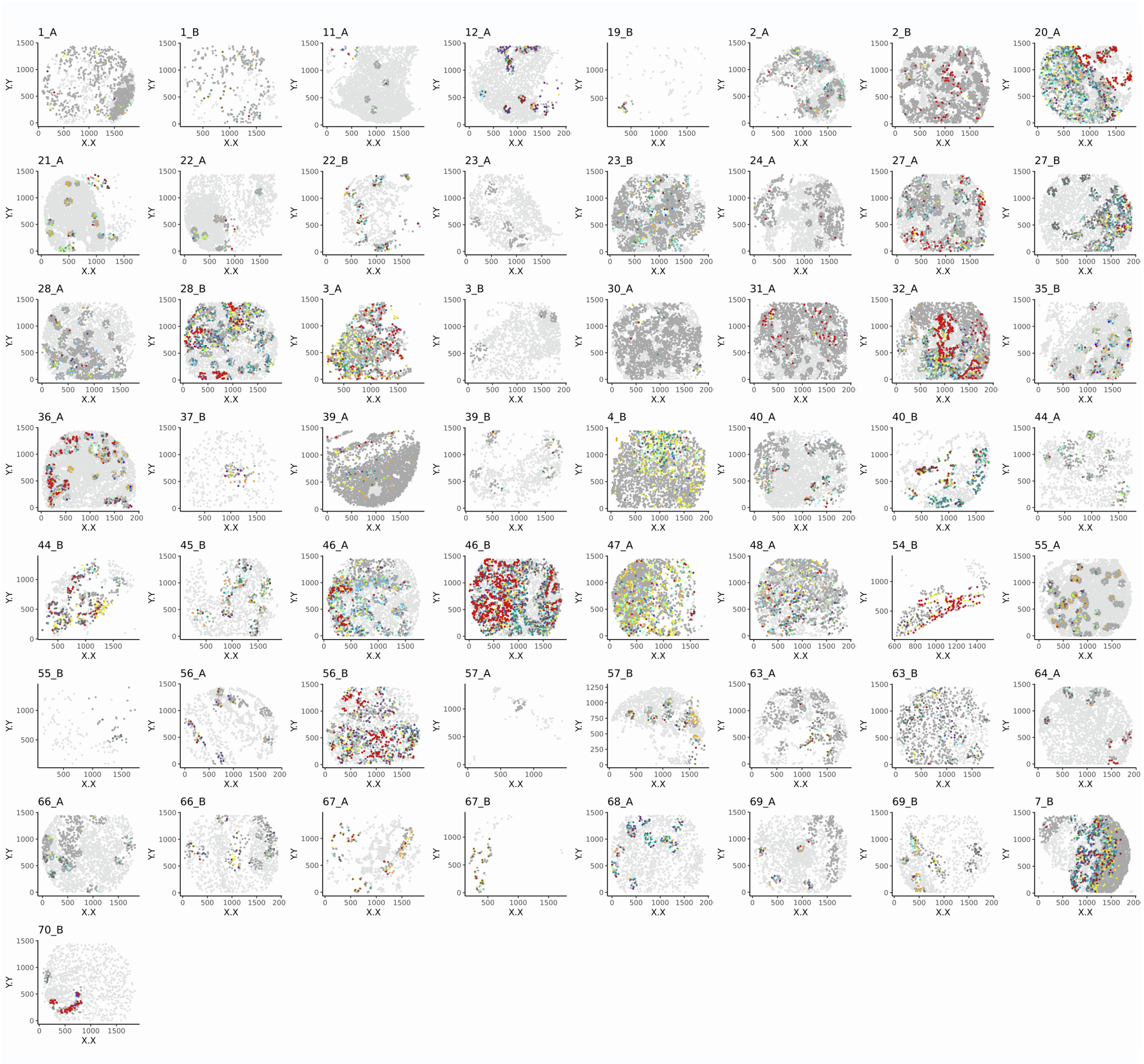

Fig. S22.

Selected cells (colored by cell type) using S^3-CIMA functional spatial enrichment analysis with granulocytes as the anchor and EGFR marker expression as the phenotype mapped back to the corresponding patient CODEX images in the **EGFR low** group. (Filter 2) .

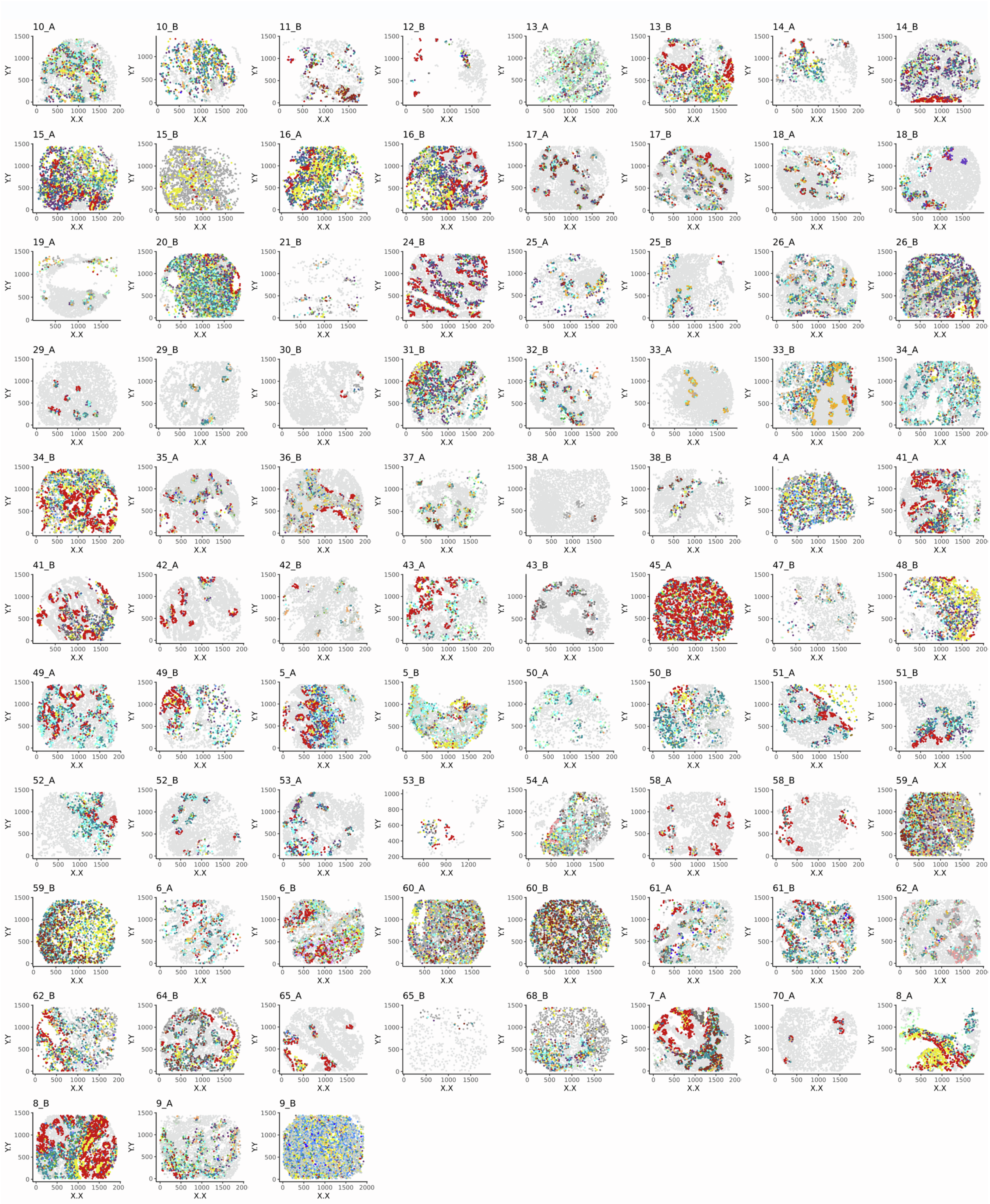

Fig. S23.

Selected cells (colored by cell type) using S^3-CIMA functional spatial enrichment analysis with granulocytes as the anchor and EGFR marker expression as the phenotype mapped back to the corresponding patient CODEX images in the **EGFR high** group. (Filter 2).

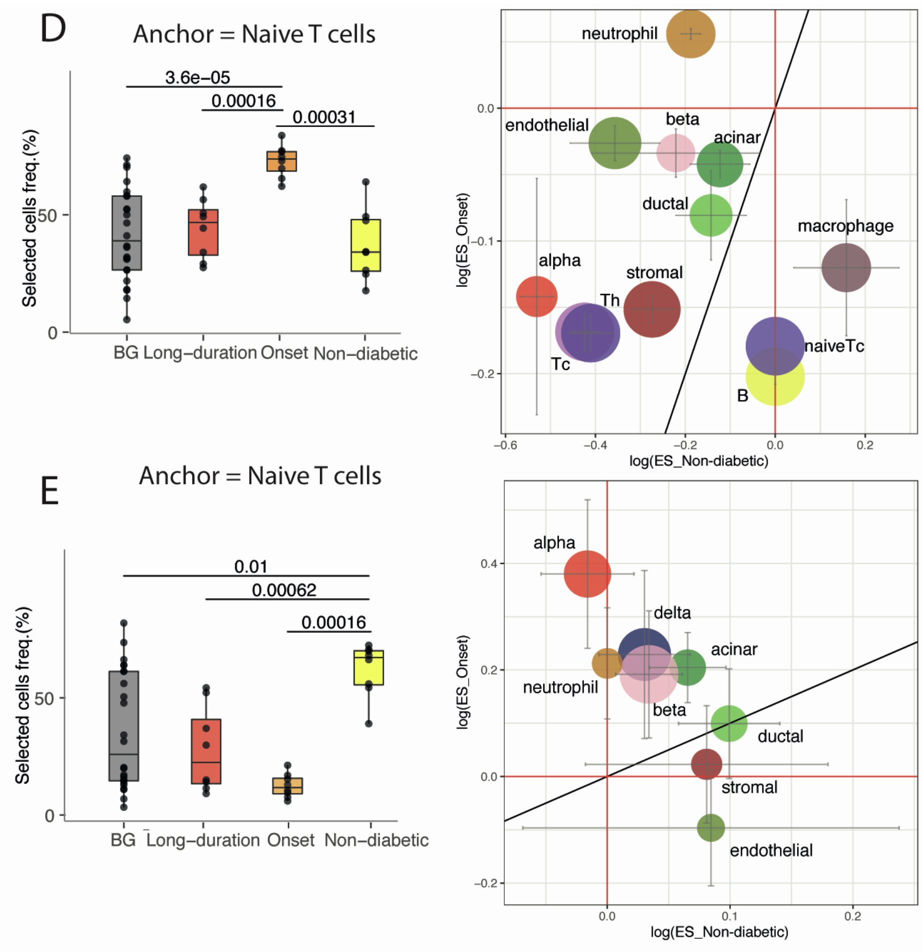

Fig. S24.
S^3-CIMA anchor based spatial enrichment analysis with Naïve T cells as the anchor.

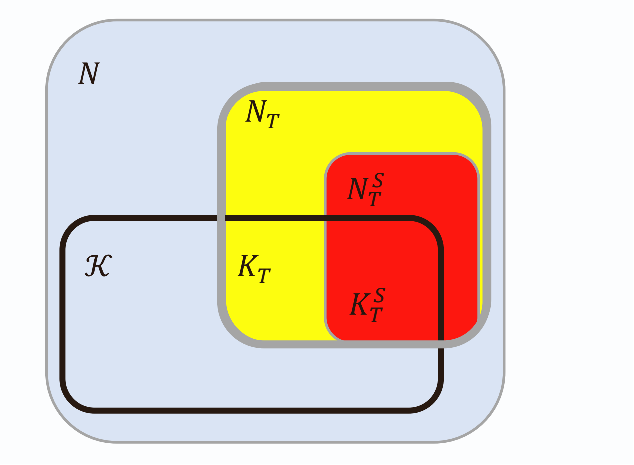

Fig. S25.

The enrichment score (ES) quantifies the association or exclusion of selected cells of specific cell type in the spatial proximity of the anchor cell. The variables N is the number of all cells in the image, K is the number of all cells in the nearest neighborhood of the anchor cell, N_T is the number of all cells of cell type T in the image, K_T is the number of cell type T in the nearest neighborhood of the anchor cell, N_T^S is the number of all selected cells of type T and K_T^S is the number of selected cell type T in the nearest neighborhood of the anchor cell.

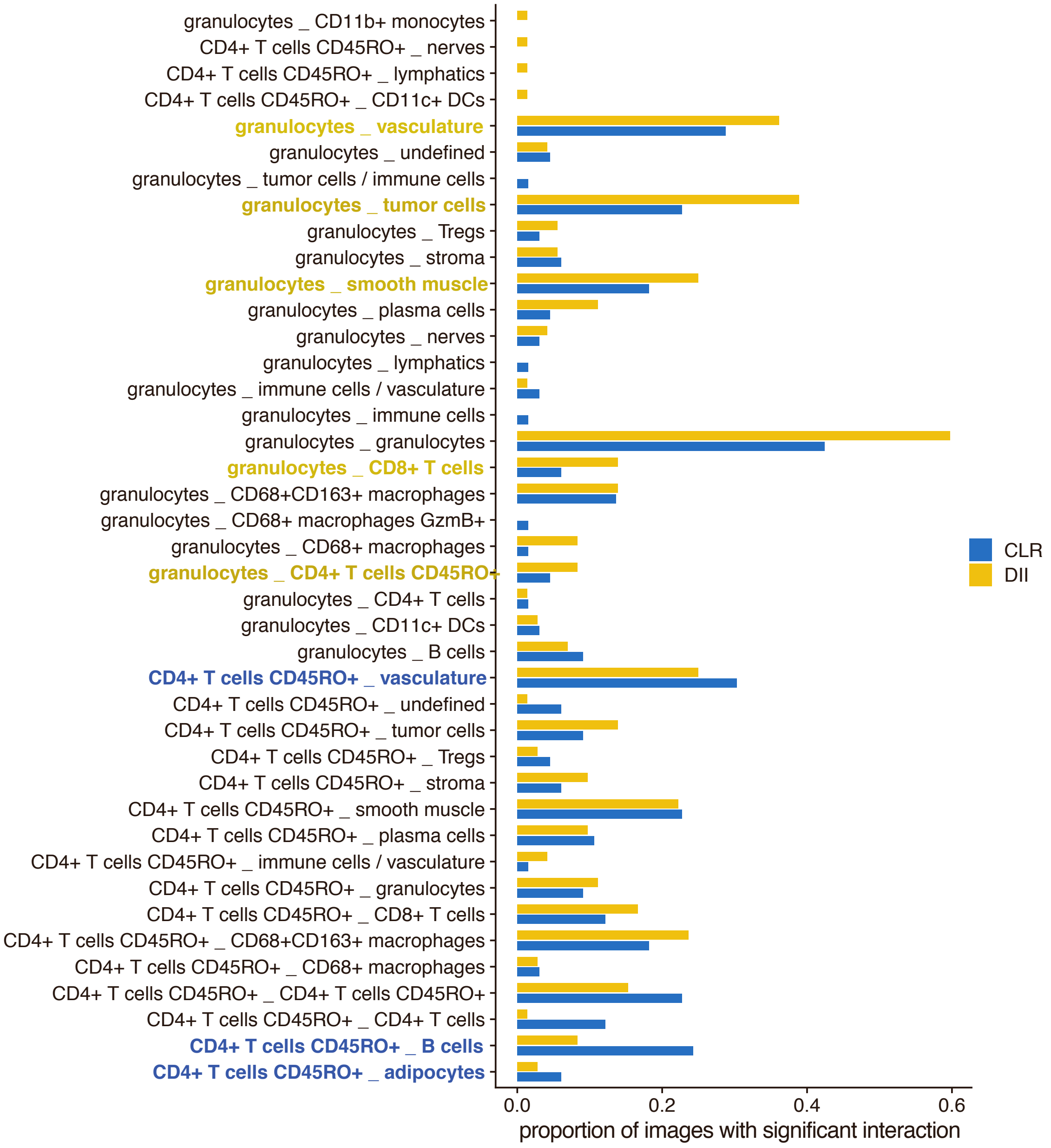

Fig. S26.
We applied the unsupervised histoCAT analysis to the data of the CRC cohort. The method enables unsupervised spatial interrogation of cell–cell interactions. The neighborhood analysis in histoCAT examines if a certain cell type is located significantly closer to another cell type in each tissue image than expected by chance using two individual one-tailed permutation tests. For each specific cell–cell interaction, we calculated the proportion of tissue images in which that interaction is significant per survival group and highlighted an interaction if the proportion is high in one of the groups.

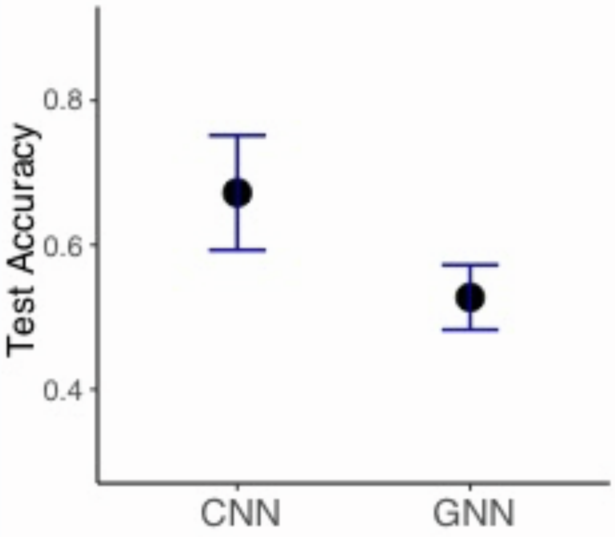

Fig. S27.
Comparing the prediction accuracy between the Graph Neural Networks (GNN) and the CNN model for global spatial enrichment analysis. The GNN model consists of a ReLu activation function, a pooling layer, and a classification output. It takes into account the marker expression of the cells in each multi-cell input to construct the neighborhood graph. To compare its performance with the CNN model, we used an identical multi-cell input of the global spatial enrichment analysis of S3-CIMA (i.e., k-NN cells of random anchor cells) to construct the neighborhood graph, while maintaining the same training and test sets. The standard GNN yielded lower classification accuracy.
